# Decoding chromatin states by proteomic profiling of nucleosome readers

**DOI:** 10.1038/s41586-024-07141-5

**Published:** 2024-03-06

**Authors:** Saulius Lukauskas, Andrey Tvardovskiy, Nhuong V. Nguyen, Mara Stadler, Peter Faull, Tina Ravnsborg, Bihter Özdemir Aygenli, Scarlett Dornauer, Helen Flynn, Rik G. H. Lindeboom, Teresa K. Barth, Kevin Brockers, Stefanie M. Hauck, Michiel Vermeulen, Ambrosius P. Snijders, Christian L. Müller, Peter A. DiMaggio, Ole N. Jensen, Robert Schneider, Till Bartke

**Affiliations:** 1 Institute of Functional Epigenetics, Helmholtz Zentrum München, Neuherberg, Germany; 2 MRC Laboratory of Medical Sciences (LMS), London, UK; 3 Department of Chemical Engineering, Imperial College London, London, UK; 4 Institute of Clinical Sciences (ICS), Faculty of Medicine, Imperial College London, London, UK; 5 Institute of Computational Biology, Helmholtz Zentrum München, Neuherberg, Germany; 6 Department of Statistics, Ludwig Maximilian University Munich, Munich, Germany; 7 Proteomic Sciences Technology Platform, The Francis Crick Institute, London, UK; 8 VILLUM Center for Bioanalytical Sciences and Department of Biochemistry and Molecular Biology, University of Southern Denmark, Odense, Denmark; 9 Department of Molecular Biology, Faculty of Science, Radboud Institute for Molecular Life Sciences, Oncode Institute, Radboud University Nijmegen, Nijmegen, The Netherlands; 10 The Netherlands Cancer Institute, Amsterdam, The Netherlands; 11 Metabolomics and Proteomics Core, Helmholtz Zentrum München, Munich, Germany; 12 Center for Computational Mathematics, Flatiron Institute, New York, NY USA; 13 Faculty of Biology, Ludwig Maximilian University Munich, Martinsried, Germany; 14 German Center for Diabetes Research (DZD), Neuherberg, Germany; 15 Present Address: Northwestern Proteomics Core Facility, Northwestern University, Chicago, IL USA; 16 Present Address: Clinical Protein Analysis Unit (ClinZfP), Biomedical Center (BMC), Faculty of Medicine, Ludwig Maximilian University Munich, Martinsried, Germany

**Keywords:** Protein-protein interaction networks, Protein databases, Proteome informatics, Histone post-translational modifications, Epigenetics

## Abstract

DNA and histone modifications combine into characteristic patterns that demarcate functional regions of the genome^1,2^. While many ‘readers’ of individual modifications have been described^3–5^, how chromatin states comprising composite modification signatures, histone variants and internucleosomal linker DNA are interpreted is a major open question. Here we use a multidimensional proteomics strategy to systematically examine the interaction of around 2,000 nuclear proteins with over 80 modified dinucleosomes representing promoter, enhancer and heterochromatin states. By deconvoluting complex nucleosome-binding profiles into networks of co-regulated proteins and distinct nucleosomal features driving protein recruitment or exclusion, we show comprehensively how chromatin states are decoded by chromatin readers. We find highly distinctive binding responses to different features, many factors that recognize multiple features, and that nucleosomal modifications and linker DNA operate largely independently in regulating protein binding to chromatin. Our online resource, the Modification Atlas of Regulation by Chromatin States (MARCS), provides in-depth analysis tools to engage with our results and advance the discovery of fundamental principles of genome regulation by chromatin states.

## Abstract (web summary)

A multidimensional proteomics analysis of the interactions between around 2,000 nuclear proteins and over 80 modified dinucleosomes representing promoter, enhancer and heterochromatin states provides insights into how chromatin states are decoded by chromatin readers.

## Main

Almost all genetic material of eukaryotic cells is stored in the nucleus in the form of chromatin, a nucleoprotein complex comprising DNA, histones and other structural and regulatory factors. DNA and histones carry chemical modifications that have central roles in chromatin regulation by either directly affecting chromatin structure or by recruiting reader proteins that mediate downstream events through specialized binding domains^4,6^. Chromatin modifications rarely occur in isolation but exist in specific combinations on histones or nucleosomes, often also involving histone variants^7–12^. As these combinations are highly correlated and predictable^13,14^, they form the basis for the definitions of ‘chromatin states’ that are used to annotate functional regions in the genome such as enhancers, promoters, gene bodies and heterochromatin^1,2^.

Most chromatin regulators contain several modification-binding domains, indicating that recognizing multiple modifications is an integral function of many nuclear proteins^15^. However, although readers of individual modifications are often well understood^3–5^, only few factors recognizing multiple modifications are known^16–24^. Thus, how complex combinatorial modification patterns underlying chromatin states are interpreted is largely unclear.

To obtain a comprehensive understanding of how chromatin readers decode different chromatin states, we have implemented a multidimensional mass spectrometry (MS)-based chromatin profiling strategy combining large-scale nucleosome affinity purification^25^ and chromatin immunoprecipitation (ChIP)–MS approaches with computational methods for the integrative analysis of high volumes of proteomics and next-generation sequencing (NGS) data. We performed over 80 affinity purification experiments with semisynthetic dinucleosomes containing modification signatures and DNA linkers representing promoter, enhancer or heterochromatin states^1,10,26^, and identified close to 2,000 nucleosome-interacting proteins, including transcription, replication, remodelling and DNA repair factors. Systematically quantifying their binding to the different modification states enabled the discovery of co-regulated proteins and complex chromatin modification read-outs driven by particular nucleosomal features, thereby revealing basic principles of how chromatin readers decode the chromatin landscape.

To make our data easily accessible, we have developed computational tools to analyse and visualize the nucleosome-binding data and we have implemented them in the interactive online resource MARCS (https://marcs.helmholtz-munich.de/). Our results bridge the gap between chromatin states and chromatin readers, and we anticipate that MARCS will become a valuable resource to drive future chromatin research forward as numerous other observations emerge.

## Proteomic profiling of chromatin readers

**Fig. 1:**
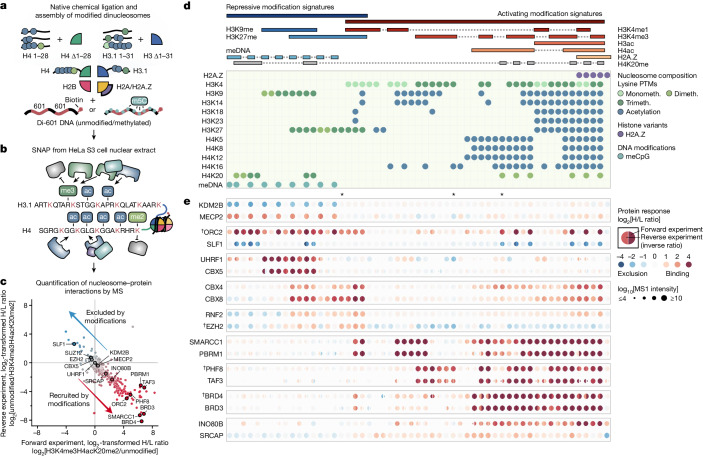
Large-scale identification of chromatin readers by SILAC dinucleosome affinity purifications. **a**, Generation of modified dinucleosomes. Modified histones H3.1 and H4 were prepared by native chemical ligations of N-terminal tail peptides (H3, amino acids 1–31; H4, amino acids 1–28) to truncated histone cores (H3.1Δ1–31T32C or H4Δ1–28I29C, respectively). Note that this introduces H3T32C and H4I29C mutations that might affect protein binding to nearby modifications. Ligated histones were refolded into octamers and assembled into dinucleosomes using a biotinylated DNA containing two nucleosome-positioning sequences (di-601)^47^. For some experiments, CpG-methylated DNA (m5C) or H2A.Z were used. **b**, SNAP purifications. Modified nucleosomes were immobilized on streptavidin beads and incubated with nuclear extracts from HeLa S3 cells grown in isotopically light (R_0_K_0_) or heavy (R_10_K_8_) SILAC medium. **c**, Protein responses to modified nucleosomes. For each SNAP experiment, bound proteins were identified and quantified using MS, and the forward (*x* axis) and reverse (*y* axis) SILAC ratios (H/L ratio) were plotted on a logarithmic (log_2_) graph. **d**, A library of modified dinucleosomes. A header specifies the modification status of each nucleosome. Nucleosomes are arranged in columns, with the respective modifications displayed in rows. Modifications of specific lysine residues in histone H3 and H4 and the presence of DNA methylation (meCpG) or H2A.Z are colour coded as indicated. Nucleosomes are ordered to imitate clustering by increasingly active chromatin states. Monometh., monomethylation; PTMs, post-translational modifications. **e**, Visualization of protein binding responses to the 55 modified dinucleosomes profiled by SNAP. The log_2_[H/L] ratios for each protein in each SNAP experiment are shown as circles, with the right half representing the forward and the left half the reverse log_2_[H/L ratio]. Recruitment (red) and exclusion (blue) are indicated. The reverse H/L ratio was inverted to display both ratios on the same scale. Circle sizes denote the total MS1 peak intensities on a log_10_ scale. The asterisks indicate experiments that are shown in Extended Data Fig. 1b–d. The dagger symbols (†) indicate proteins that are highlighted in Extended Data Fig. 1b–e.

**Extended Data Fig. 1:**
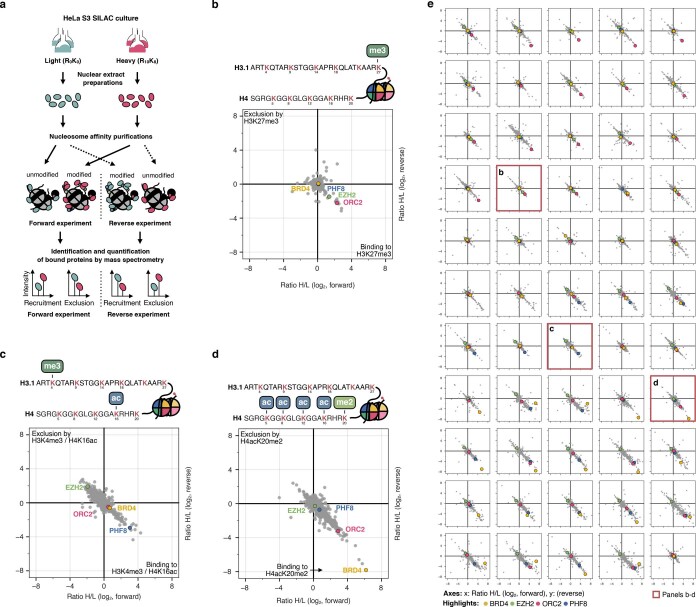
SNAP experiments reveal differential responses of chromatin readers to nucleosomal modification signatures. **a**, SILAC Nucleosome Affinity Purifications (SNAP). For SNAP experiments modified nucleosomes were immobilized on streptavidin beads and incubated with nuclear extracts from HeLa S3 cells grown in isotopically light (R_0_K_0_) or heavy (R_10_K_8_) SILAC media. In ‘forward’ experiments heavy extracts were incubated with modified and light extracts with unmodified nucleosomes, while in ‘reverse’ experiments the extracts were exchanged. Bound proteins were eluted using an on-bead digestion protocol and identified and quantified by mass spectrometry. For each SNAP experiment the SILAC ratios Heavy/Light (Ratio H/L) of the forward and reverse experiment of the identified proteins were measured and plotted in a logarithmic (log_2_) graph (see Fig. 1c and panels **b-d**). The H/L ratios indicate binding preferences to the modified or the unmodified nucleosomes and allow the unbiased identification of proteins that are either recruited or excluded by the modifications, in addition to proteins that bind nucleosomes but do not show a strong response to the modifications. **b**, Exemplary SNAP experiment with H3K27me3-modified di-nucleosomes. The results show that the ORC subunit ORC2 and the PRC2 subunit EZH2 are recruited by the H3K27me3 modification as previously reported^25,74^. **c**, SNAP experiment with H3K4me3- and H4K16ac-modified di-nucleosomes. This modification pattern recruits the H3K4me3 reader PHF8^32^ but excludes EZH2 through loss of PRC2 binding to the N-terminus of histone H3^33^. **d**, SNAP experiment with di-nucleosomes combining di-methylation of lysine 20 and acetylation of lysines 5, 8, 12, and 16 on histone H4 (H4acK20me2). This nucleosome strongly recruits BRD4 through its interaction with H4ac via its bromodomains^75^ as well as the ORC subunit ORC2 through recognition of H4K20me2 via ORC1^76^. **e**. Results for SNAPs with the entire library of 55 modified di-nucleosomes. Tracking the signals of BRD4, EZH2, ORC2, and PHF8 as highlighted in **b-d** allows interrogation of their responses to the different modification signatures. The order of SNAP experiments corresponds to the order of di-nucleosomes shown in Fig. 1d.

To systematically profile the interactomes of chromatin modifications in the nucleosomal context, we performed SILAC nucleosome affinity purification (SNAP)^25^. We assembled nucleosomes from biotinylated DNA and histone octamers containing site-specifically modified histones H3.1 and H4 prepared by native chemical ligation^27^ (Fig. 1a) and used them in forward and reverse SILAC nucleosome pull-down experiments in HeLa S3 cell nuclear extracts (Fig. 1b and Extended Data Fig. 1a). The label swap enables unbiased identification of proteins that are reproducibly either recruited or excluded by the modification(s). Moreover, the SILAC heavy/light (H/L) ratios also indicate a relative strength of recruitment or exclusion of a protein by the modifications (Fig. 1c). After optimizing our SNAP methodology (Supplementary Information) for a large-scale comparison of interactomes of different chromatin states, we used single-end biotinylated dinucleosomes in all SNAP experiments.

To understand how distinct chromatin states marked by combinations of modifications are read by binding proteins, we created a library of nucleosomes incorporating biologically relevant modification signatures, including mono- and tri-methylation of lysine 4 of histone H3 (H3K4me1/3), di- and tri-methylation of lysines 9 and 27 of histone H3 (H3K9me2/3 and H3K27me2/3), di- and tri-methylation of lysine 20 of histone H4 (H4K20me2/3), varying degrees of acetylation of lysines (Kac), the histone variant H2A.Z or CpG-methylated DNA. This design of the nucleosome library enabled us to capture the interactomes of major repressive and activating chromatin states (Fig. 1d), including enhancer, promoter and different heterochromatin states. A detailed list of modified histones, octamers and nucleosomes and corresponding quality controls is provided in the Supplementary Information.

In total we performed SILAC-linked affinity purifications with 55 dinucleosomes. The forward and reverse experiments were generally very reproducible, and we achieved high detection coverage for most of the identified proteins. After correction for batch effects and imputation of missing values (Supplementary Information), we catalogued the responses of 1,915 proteins to the various modification states (Supplementary Table 1), covering a large part of the known chromatin proteome. Collectively, the SNAP experiments not only characterize protein binding to the nucleosomal modifications but also offer systematic insights into the behaviour of chromatin readers through analysis of the changes in the H/L ratios across the entire dataset.

## MARCS maps chromatin-binding responses

Comparing the log_2_-transformed H/L ratios of individual proteins across SNAP experiments revealed characteristic nucleosome-binding behaviours (Extended Data Fig. 1b–d). To facilitate the analysis and exploration of many SNAP experiments (Extended Data Fig. 1e), we implemented the interactive online visualization resource MARCS (https://marcs.helmholtz-munich.de).

**Extended Data Fig. 2:**
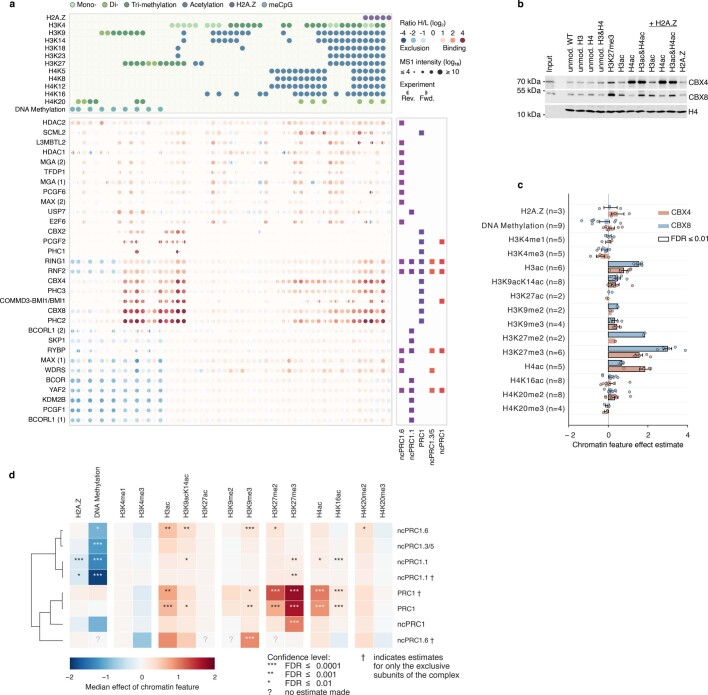
Feature effect estimates provide a breakdown of key modification determinants driving nuclear protein recruitment to chromatin. **a**, Heatmap visualization of the binding responses of PRC1 complexes to the 55 differentially modified di-nucleosomes used in the SNAP experiments. Note that subunits unique to different PRC1 sub-complexes demonstrate distinct sub-complex-specific binding behaviours, while core subunits shared between PRC1 sub-complexes show a superposition of such distinct binding behaviours. **b**, Differential enrichment of CBX4 and CBX8 in targeted pull-down experiments from HeLa S3 nuclear extracts using di-nucleosomes decorated by H3K27me3 or different combinations of H3ac, H4ac, and/or H2A.Z and evaluation by immunoblot. Note the enrichment of CBX4 in pull-downs with di-nucleosomes containing H4ac or H3K27me3 and enrichment of CBX8 with di-nucleosomes containing H3K27me3 or H3ac confirming the SNAP results. Experiments were independently repeated twice with similar results in both replicates. **c**, Chromatin feature effect estimates of the nucleosome binding response of the CBX4 and CBX8 subunits of the canonical PRC1 complex. The bars highlight the limma effect estimates (change in log_2_ ratio H/L) for each subunit for the 15 different chromatin features. N indicates the number of nucleosome pairs informative of the different chromatin features that were used to calculate the effect estimate (see also Extended Data Fig. 3a, b and Supplementary Table 3), where the points represent the mean change in the log_2_ ratio H/L per pull-down pair. The error bars represent the 95% CI of the effect estimates (limma). Statistically significant effects (limma, two-sided, Benjamini/Hochberg-adjusted FDR ≤ 0.01) are highlighted in black frames. Note the distinct binding profiles, where CBX4 recruitment to di-nucleosomes is stimulated predominantly by H4ac and to a lesser extent by H3K27me3, while CBX8 recruitment is stimulated by H3K27me2/3 and to a lesser extent by H3ac, directly reflecting the immunoblot validation shown in **b**. **d**, Heatmap depicting the chromatin feature effects of the nucleosome binding responses of different PRC1 sub-complexes. The median feature effect estimates across all complex subunits with protein response measurements for a given feature are displayed for the different complexes as indicated in the colour key. In order to disambiguate variant-specific responses, the feature effect estimates for only exclusive subunits of the different complexes are shown as separate rows in the data. Statistically significant associations are indicated with asterisks (CAMERA, Benjamini/Hochberg-adjusted FDR: * ≤ 0.01, ** ≤ 0.001, *** ≤ 0.0001). Cells where no statistical estimate could be made due to insufficient data are marked with “?”. See also Supplementary Table 8.

Figure 1d,e shows an exemplary set of heat maps generated using MARCS. The clustered heat map of all proteins is provided in Supplementary Table 2. Our data capture a broad range of responses by chromatin readers to repressive and activating modification states and thereby reveal two principle modes of interaction: simple responses to single modifications as exemplified by the recruitment of MECP2 or exclusion of KDM2B by DNA methylation (Fig. 1e); and complex binding patterns indicating binding to multiple modifications or synergistic responses as illustrated by the origin recognition complex (ORC) that shows recruitment to H3K9, H3K27 or H4K20 methylations, with further stimulation by DNA methylation (ORC2 in Fig. 1e). Importantly, while these examples constitute internal controls by consistently showing known and expected binding behaviours, our broad and unbiased profiling of chromatin states also enables the identification of interactions with modified nucleosomes in new contexts. For example, we find that the INO80 chromatin remodelling complex^28^ and polycomb repressive complex 1 (PRC1)^29^ are enriched on nucleosomes displaying active modification signatures, including acetylations of the histone H3 and H4 N-terminal tails (INO80B for INO80 in Fig. 1e; CBX4 and CBX8 for PRC1 in Fig. 1e and Extended Data Fig. 2a,b).

## Unbiased prediction of binding features

**Fig. 2:**
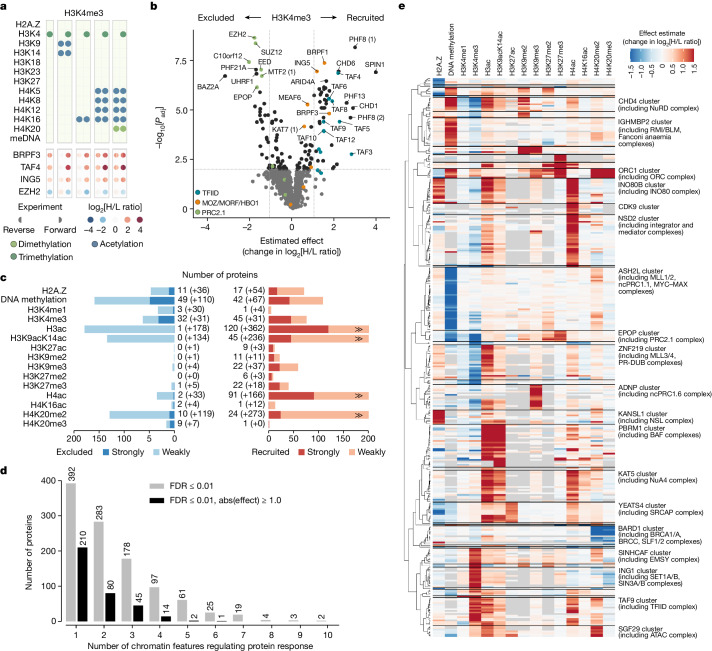
Feature effect estimates reveal binding responses of chromatin readers to different nucleosomal features. **a**, Nucleosomes informative of protein responses to H3K4me3. The four pairs of dinucleosomes that differ only by H3K4me3, alongside the self-informative H3K4me3 dinucleosome (top), and the binding responses of four representative proteins in the corresponding SNAP experiments (bottom) are shown. **b**, Feature effect estimates of proteins showing H3K4me3-dependent nucleosome binding. The change in the log_2_[H/L ratio] attributable to H3K4me3 (*x* axis) is plotted against the *P* value (limma, two-sided, Benjamini–Hochberg adjusted) on a −log_10_ scale (*y* axis). The vertical lines highlight an effect to fold change of 1, and the horizontal line signifies the FDR threshold of 0.01. Selected protein complexes are highlighted. Duplicate protein identifiers, for example, PHF8 (1), mark distinct UniProt IDs with the same gene name (Trembl versus SwissProt versions); for annotations, see Supplementary Table 1. **c**, The number of interactors responsive to different chromatin features. Owing to their frequent co-occurrence, blocks of acetylation, such as H3K9acK14ac, H3K9acK14acK18acK23acK27ac (H3ac) and H4K5acK8acK12acK16ac (H4ac) were treated as single features. Proteins with statistically significant (limma, FDR ≤ 0.01) effect estimates ≥ 1 classify as strongly recruited, or strongly excluded if their estimate is ≤−1. Changes in log_2_[H/L ratio] < 1 are considered to be weakly recruited or excluded. **d**, The number of chromatin features regulating protein binding responses. The grey bars tally the number of proteins with statistically significant feature effects (limma, FDR ≤ 0.01). The black bars additionally tally proteins with strong feature effects (absolute effect ≥ 1). **e**, Clustered heat map of feature effect estimates of proteins strongly responding to at least one feature as shown in **c**. Individual estimates are colour coded. Entries without an estimate due to insufficient data are marked in grey. Prototype proteins representing the binding response of each cluster are shown on the right. Notable protein complexes are highlighted.

**Extended Data Fig. 3:**
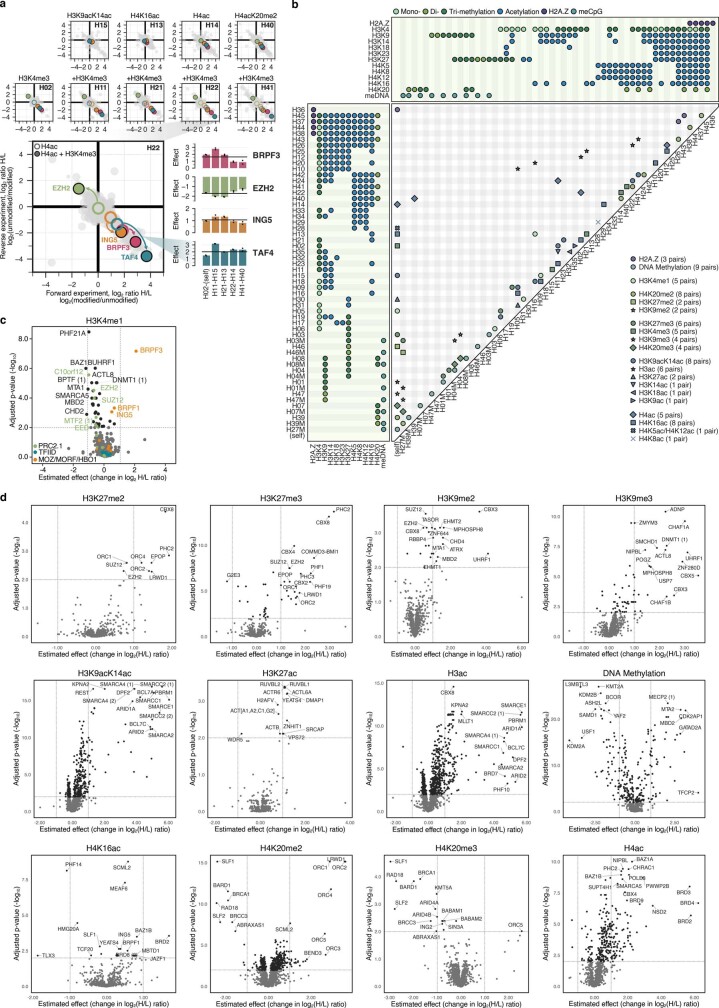
Effect estimates of protein responses to chromatin features. **a**, Comparisons of SNAP experiments performed to determine the effect of H3K4me3 on protein binding. SNAP experiments of nucleosome pairs informative of H3K4me3 are shown in the upper panel. Experiment H22 (H4ac+H3K4me3) is shown enlarged below. Protein positions of the four exemplary proteins highlighted in Fig. 2a are indicated in the scatter plots. Protein positions in the paired nucleosomes lacking the H3K4me3-modification are shown by empty circles in the scatterplots of the corresponding nucleosomes containing the H3K4me3-mark to highlight changes in position. Imputed values are plotted with smaller dots. The mean of the changes in the log_2_ H/L ratios of the forward and reverse experiment (n = 2 biologically independent pull-down experiments) are highlighted in the bar plots for the individual comparisons for each of the selected proteins as shown. The black lines indicate the feature effect estimates for H3K4me3 for the four proteins derived by limma based on all comparisons from all H3K4me3-informative pull-down pairs (see also Fig. 2b). SNAP experiment identifiers are listed in panel **b**, Supplementary Table 1, and the Supplementary Information. **b**, Matrix of pairs of di-nucleosomes which are informative of chromatin modification features. Pairs are identified as defined in the legend in the bottom right corner. The leftmost column and bottom row indicate nucleosomes which contain only the modification of interest and are therefore self-informative. SNAP experiment identifiers are listed in Supplementary Table 1 and Supplementary Information. Only features with two or more informative pairs of nucleosomes, and therefore an independent experimental replicate, were quantified for the feature effect estimates. **c**, Volcano plot of H3K4me1-responsive proteins. Data representation and labelling of selected protein complexes as in Fig. 2b. Duplicate protein identifiers with numbers in parentheses, e.g. DNMT1 (1), correspond to distinct UniProt IDs with the same gene name (i.e. Trembl vs. SwissProt versions), see also annotations in Supplementary Table 1. **d**, Volcano plots showing the effect estimates for the protein responses to the 12 (out of 15) chromatin features not highlighted in panel **c**, Fig. 2b, and Extended Data Fig. 5a. Data representation as in Fig. 2b. Selected proteins are highlighted.

**Extended Data Fig. 4:**
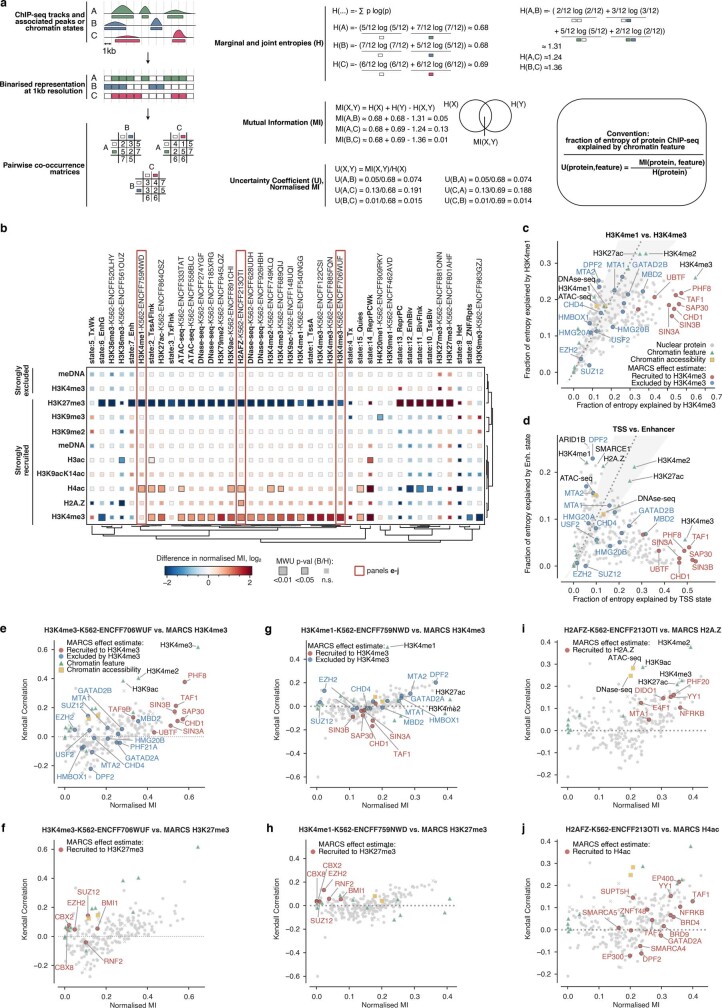
Integrative analysis of MARCS with ENCODE ChIP-seq datasets. **a**, Schematic representation of the integrative NGS dataset analysis. Briefly, the peak data for the datasets was binned at 1 kb resolution. For each pair of datasets, the pairwise co-occurrence matrix was recorded, tracking the number of bins in which the peaks overlap. The marginal and joint entropies, together with the mutual information (MI), were computed from the co-occurrence matrices. Note, as the mutual information measures the entropy shared by the two proteins (venn diagram) it can be normalized via the entropy of one of the two factors. Since in MARCS we are interested in the explanatory power of chromatin features on protein binding, by convention we always normalized by the entropy of the protein. The normalized mutual information estimates are therefore interpretable as the fraction of uncertainty in protein localization that can be explained by the feature. For details see online methods. **b**, Summary of the relationships between MARCS feature effect estimates and NGS datasets for the Tier 1 ENCODE K562 cell line. The ChIP-seq, ATAC-seq, and DNase-seq experiments from ENCODE^30^ are plotted in columns together with the chromatin state annotations from the NIH Roadmap^1^. The rows represent MARCS protein groups subdivided by their feature effect estimates, only groups with ≥5 proteins are shown. Each cell of the heatmap indicates two measurements that contrast the normalized MI (see **a**) for proteins that MARCS predicts to be strongly recruited or excluded by the feature to the normalized MI of other proteins (i.e. proteins neither *strongly* recruited nor *strongly* excluded by the feature, including proteins with no feature effect estimate at all). The colour indicates the difference between the mean log_2_ of the normalized MI estimates in the feature-associated group versus the mean of the log_2_ estimates of other proteins. The size and the border shading of the square indicates the statistical significance of the difference (Mann-Whitney U test, two-sided, Benjamini/Hochberg-adjusted). See the colour bar and the legend. Significant red colours indicate that a given chromatin feature ChIP-seq experiment is more predictive of ChIP-seqs of proteins associated with a given MARCS-feature than ChIP-seqs of an average protein. Significant blue colours indicate the opposite. The rows and columns were clustered hierarchically to highlight similarities. **c**, Integrative analysis of ENCODE NGS data for the K562 cell line in relation to H3K4me1 and H3K4me3 ChIP-seq peaks. The fraction of entropy of a protein or feature explained by the information about H3K4me3 and H3K4me1 peaks is plotted on the x and y axes, respectively. Larger values indicate stronger mutual information between the peak distributions. The dotted x = y line indicates where H3K4me1 and H3K4me3 have exactly the same explanatory power. The shaded area corresponds to ± 0.2 radians from this line. MARCS feature estimates for H3K4me3 are indicated in red (strong recruitment) or blue (strong exclusion). Proteins without strong recruitment or exclusion are shown in grey, no feature effect estimate is marked by “X”. **d**, Integrative analysis as in **c** performed for NIH Roadmap promoter (x axis) and enhancer (y axis) chromatin states. Note, that MARCS H3K4me3 readers again share higher mutual information with the promoter chromatin state than the enhancer state. Only a few BAF complex subunits (SMARCE1, ARID1B) show a weak preference for enhancers. Data representation is as in **c**. **e**, Integrative analysis of ENCODE NGS data for the K562 cell line in relation to one of the H3K4me3 ChIP-seq replicates (highlighted in **b**). Normalized MI (i.e. fraction of entropy of proteins/chromatin features explained by the H3K4me3 ChIP-seq) is plotted on the X axis, while the Kendall correlation coefficient of overlapping peak heights is plotted on the Y axis. Protein datasets are plotted in grey, while chromatin feature and accessibility datasets are plotted in green and yellow, respectively. Proteins strongly recruited to H3K4me3 based on their MARCS feature effect estimates are highlighted in red, and strongly excluded proteins are highlighted in blue. Note that proteins strongly recruited to H3K4me3 have, on average, higher normalized MI estimates than others (Mann-Whitney U test, two-sided, Benjamin/Hochberg-adjusted FDR < 0.01). **f**, Data in **e** plotted with proteins strongly recruited to H3K27me3 based on MARCS feature effect estimates highlighted in red. Note that these proteins have on average lower normalized MI estimates than others (Mann-Whitney U test, two-sided, Benjamin/Hochberg-adjusted FDR < 0.05). **g**, Integrative analysis of ENCODE NGS data for the K562 cell line in relation to one of the H3K4me1 ChIP-seq replicates (highlighted in **b**). Data presented as in **e**. Proteins strongly recruited to H3K4me3 based on MARCS feature effect estimates are highlighted in red, and strongly excluded are highlighted in blue. Note that there is no statistically significant difference between these proteins and other proteins (Mann-Whitney U test, two-sided, Benjamin/Hochberg-adjusted FDR < 0.05). **h**, Data in **g** plotted with proteins strongly recruited to H3K27me3 based on MARCS feature effect estimates highlighted in red. Note that these proteins have on average lower normalized MI estimates than others (Mann-Whitney U test, two-sided, Benjamin/Hochberg-adjusted FDR < 0.01). **i**, Integrative analysis of ENCODE NGS data for the K562 cell line in relation to the H2A.Z ChIP-seq (highlighted in **b**). Data presented as in **e**. Proteins strongly recruited to H2A.Z based on MARCS feature effect estimates are highlighted in red. Note that these proteins have on average higher normalized MI estimates than others (Mann-Whitney U test, two-sided, Benjamin/Hochberg-adjusted FDR < 0.05). **j**, Data in **i** plotted with proteins strongly recruited to H4ac based on MARCS feature effect estimates highlighted in red. Note that these proteins have on average higher normalized MI estimates than others (Mann-Whitney U test, two-sided, Benjamin/Hochberg-adjusted FDR < 0.01).

**Extended Data Fig. 5:**
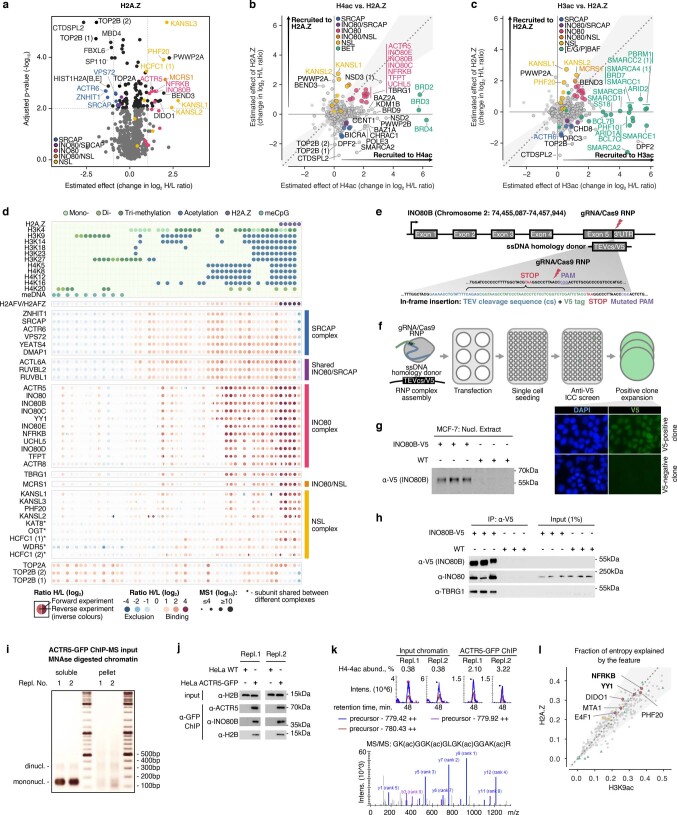
The INO80 complex interacts with TBRG1 and recognizes a multivalent H3ac/H4ac-H2A.Z modification signature. **a**, Volcano plot of H2A.Z-responsive proteins. Data representation as in Fig. 2b. NSL, SRCAP, and INO80 complex subunits are highlighted. NSL subunits are significantly enriched (CAMERA, Benjamini/Hochberg-adjusted FDR ≤ 0.01, see Supplementary Table 8) while TOP2B is negatively regulated by H2A.Z. **b**, Breakdown of protein responses to H4ac and H2A.Z. Data representation as in Fig. 3a. Selected protein complexes are highlighted. Note that the INO80 complex responds to both H4ac and H2A.Z. **c**, Breakdown of protein responses to H3ac and H2A.Z. Data representation as in Fig. 3a. Note that the INO80 complex responds to both H3ac and H2A.Z. **d**, Heatmap visualization of the binding responses of SRCAP, INO80, and NSL complex subunits to the 55 modified di-nucleosomes. The complexes respond to multiple chromatin modification states which are strongly modulated by H2A.Z. Note that the nucleosome response profile of H2A.Z itself (H2AFV/H2AFZ) is similar to the SRCAP complex except in the five nucleosomes with recombinant H2A.Z, consistent with SRCAP’s role in H2A.Z loading^77^. The TBRG1 binding profile follows that of INO80 subunits. The ACTL6A/RUVBL1/RUVBL2 module is shared between INO80, SRCAP, and other complexes, its binding pattern indicates preferential localization to the SRCAP complex. The NSL complex is enriched by H2A.Z-containing nucleosomes, albeit some of the subunits (marked with asterisk) show divergent binding properties due to their preferential localization in other complexes. H2A.Z also differentially regulates the interaction of the two DNA Topoisomerase II isoforms α and β (TOP2A and TOP2B) with nucleosomes. While TOP2A binds H2A.Z-containing nucleosomes, TOP2B binding is clearly hindered by H2A.Z. Proteins labelled twice with enumerated labels e.g. (1) and (2) correspond to multiple Uniprot identifiers mapped to the same gene name (e.g. SwissProt and TrEMBL identifiers), see also annotations in Supplementary Table 1. **e**, Schematic representation of the endogenous INO80B tagging strategy in MCF-7 cells. A gRNA was designed to cut in the 3′ UTR of the INO80B gene close to the stop codon (position −11). A single-stranded (ss) DNA oligonucleotide containing a TEV protease cleavage sequence (TEVcs) followed by the V5-tag sequence prior to the stop codon was used as homology donor. **f**, Workflow of the clonal MCF-7 cell line generation. Ribonucleoprotein (RNP) complexes were assembled from a two-piece gRNA (crRNA:tracrRNA duplex) and Cas9 protein and mixed with the ssDNA template. Cells were transfected with the RNP/ssDNA mixture and after 48 h seeded in 96 well plates with one cell per well. V5-positive clones were selected using immunocytochemistry (ICC) with anti-V5 antibodies. Note that the localization of the anti-V5-staining is nuclear as evidenced by the overlap with the DNA (DAPI) staining. Positive clones were expanded, characterized (see panel **g**), and used for further experiments. **g**, Immunoblot validation of the INO80B-V5 tagging. Nuclear extracts from three independently isolated V5-positive clonal MCF-7 cell lines used for the n = 3 INO80B-V5 IP-MS experiments shown in Fig. 5b,c were resolved by SDS PAGE and probed with anti-V5 antibodies to verify the endogenous tagging of INO80B with the V5 tag. Nuclear extracts from three independently isolated V5-negative cell lines (WT) were used as controls. **h**, TBRG1 co-purifies with the INO80 complex. Immunoblot analyses of n = 3 independent biological co-IP experiments of endogenously V5-tagged INO80B (INO80B-V5) using nuclear extracts prepared from the three clonal MCF-7 knock-in cell lines shown in panel **g**. INO80B was immunoprecipitated via the C-terminal V5-tag. TBRG1 co-purifies with INO80B along with the INO80 core subunit. The panel shows all three replicates, see Fig. 5b,c for the mass spectrometric quantification. WT indicates the three V5-negative MCF-7 cell lines shown in panel **g** that were used as negative controls for the V5 immunoprecipitation. **i**, Ethidium bromide-stained agarose gel showing DNA isolated from two independently prepared MNAse-digested HeLa chromatin samples used as input for the n = 2 replicates of the native ChIP-MS analysis of mono-nucleosomes co-purified with the GFP-tagged INO80 subunit ACTR5 shown in panel **k** and Fig. 5f. Shown are the soluble fraction used as input for the ChIP-MS and the undigested chromatin remaining in the pellet for both replicates of the preparation. Both replicates yielded similar results. **j**, Immunoblot analysis of native anti-GFP ChIPs using MNAse digested chromatin from WT HeLa cells and HeLa cells expressing the GFP-tagged INO80 subunit ACTR5. Purified proteins and co-purified mono-nucleosomes were released from GFP-Trap beads using 3 C protease, resolved by SDS-PAGE, transferred to nitrocellulose membranes and probed with specified antibodies. Note that ACTR5-GFP co-purifies the INO80 complex and histone proteins (indicated by co-IP of INO80B and H2B), which are absent in the control purifications from WT HeLa cells, verifying high specificity of the ChIP procedure. Experiments were repeated in n = 2 biologically independent replicates with similar results. The panel shows the results from both replicates. **k**, Extracted ion chromatograms for the H4K5acK8acK12acK16ac histone peptide (H4-4ac) in the input and ACTR5-GFP (INO80) ChIP-MS nucleosome co-purification samples (top panel), and representative annotated MS2 spectrum of the H4K5acK8acK12acK16ac peptide (bottom panel). The top panel shows the results for the H4-4ac peptide in both replicates of the ACTR5-GFP ChIP-MS displayed in Fig. 5f. **l**, Integrative analysis of ENCODE^30^ NGS data for the K562 cell line in relation to H3K9ac and H2A.Z genomic distributions. Data representation as in Extended Data Fig. 4c. Proteins strongly recruited by H2A.Z based on their MARCS feature estimates are highlighted in red, INO80 subunits are highlighted in bold. INO80 subunits are among the top-scoring proteins whose genomic distribution can be explained by both H2A.Z and H3K9ac.

Inspection of the heat maps further revealed that many proteins exhibit broad nucleosome binding responses that cannot be explained by one single feature, that is, a particular histone modification, DNA methylation or the H2A.Z variant alone. To describe such complex binding behaviours, we deconvoluted the SNAP binding profiles into individual nucleosomal features driving these associations. We achieved this by comparing log_2_[H/L ratio] values between related nucleosomes that differ by only one single feature. For example, four pairs of dinucleosomes are informative of the effect of H3K4me3 on protein binding (Fig. 2a). A consistent increase or decrease in the log_2_[H/L ratio] across these nucleosome pairs can be attributed only to H3K4me3, irrespective of other modifications that the chromatin reader may recognize. Repeatedly sampling this effect across multiple nucleosome pairs, in addition to the H3K4me3 dinucleosome-purification experiment itself (Extended Data Fig. 3a), enables statistical evaluation and calculation of a ‘feature effect estimate’ expressed as the H3K4me3-dependent change in the log_2_[H/L ratio] for a particular protein (Fig. 2b). This way, we were able to resolve the responses of chromatin readers to 15 different modification features resulting from 82 pairs of nucleosomes (Fig. 2b, Extended Data Figs. 3b–d and 5a and Supplementary Table 3). The feature effect estimates enable us to quantitatively describe the chromatin-binding behaviours of several hundred proteins and provide a breakdown of complex binding profiles into a set of key features that either positively or negatively regulate their association with the modified nucleosomes (Extended Data Fig. 2c,d). We have implemented this decomposition of binding profiles into ‘chromatin feature motifs’ in the MARCS online resource. Importantly, an integrative analysis of public ENCODE^30^ ChIP followed by sequencing (ChIP–seq) datasets covering a subset of identified nucleosome-interacting proteins and relevant chromatin features demonstrates that the binding behaviours observed in our in vitro dinucleosome system recapitulate the binding behaviours found in cellular chromatin (Extended Data Fig. 4a–j and Supplementary Table 4).

Notably, the number of proteins responding to each of the 15 features is highly variable, with euchromatic features such as H3ac or H4ac recruiting or excluding many more proteins than heterochromatic ones such as H3K9me2/3 or H3K27me2/3 (Fig. 2c). However, this might be biased by the extract preparation method, which preferentially releases euchromatic proteins. Furthermore, many proteins are regulated by more than one feature (Fig. 2d,e) indicating that they either respond to multiple modifications independently or recognize composite modification signatures. Clustering of individual protein binding behaviours revealed that they can be grouped into 40 major binding responses, largely defined by multisubunit protein complexes (Fig. 2e and Supplementary Table 5). For example, multiple factors such as the INO80, MLL3/4, NuA4 or TFIID complexes show highly specific responses to the different ‘promoter state’ features H3K4me3, H3ac, H4ac and H2A.Z. Whereas binding of, for example, the INO80 remodeller^28^ is stimulated by H2A.Z in addition to H3 and H4 acetylation (Extended Data Fig. 5a–c), the NuA4 histone acetyltransferase complex responds similarly to H3 and H4 acetylation, but not H2A.Z (Fig. 2e). This complex regulation of INO80 by a H3ac/H4ac–H2A.Z axis was not directly apparent from the original SNAP data (Extended Data Fig. 5d), illustrating how the feature effect estimates can be used to decode nucleosome-binding determinants across entire chromatin states.

## Absence of distinctive H3K4me1 readers

**Fig. 3:**
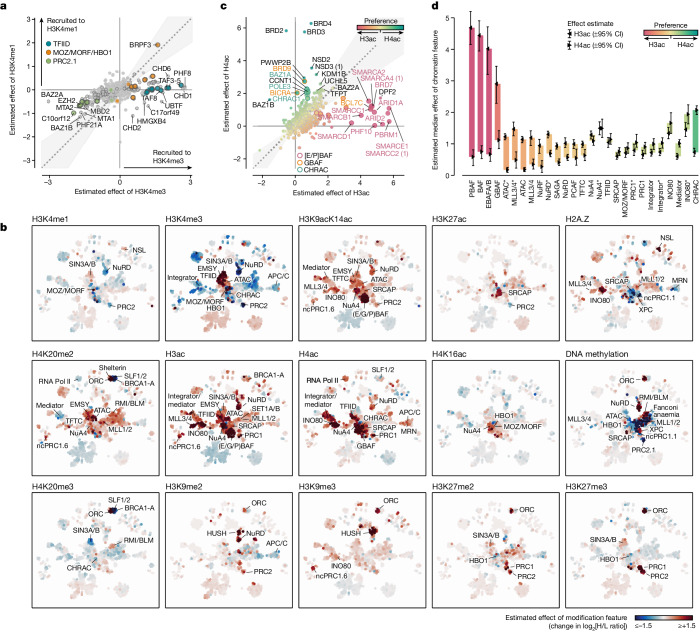
Differential binding of proteins to H3K4 methylation and H3/H4 acetylation states. **a**, Comparison of H3K4me3- versus H3K4me1-responsive proteins. H3K4me3- or H3K4me1-dependent changes in the log_2_[H/L ratio] are plotted on the *x* and *y* axes, respectively. Proteins with statistically significant estimates (limma, two-sided, Benjamini–Hochberg-adjusted FDR ≤ 0.01) are circled with a grey border. The grey area marks ±0.2 radians away from the *x* = *y* line. Selected protein complexes are highlighted. While H3K4me1 recruits only BRPF3 but no other interactors, it still excludes, for example, the PRC2 complex, albeit not as strongly as H3K4me3. **b**, CLR-predicted network overlayed with chromatin feature effects. The heat maps reveal the degree and specificity of protein recruitment or exclusion by the different features. Protein complexes with statistically significant regulation (CAMERA, FDR ≤ 0.01, median effect ≥ 0.3; Supplementary Table 8) were annotated for each feature after manual curation. A zoomable version is provided in the MARCS resource. **c**, Comparison of proteins responding to H3 versus H4 acetylation. Changes in the log_2_[H/L ratio] attributable to H3ac or H4ac are plotted on the *x* and *y* axes, respectively. Data representation as in **a**. Proteins are coloured by the difference between their H3ac and H4ac responses. BAF and CHRAC complex subunits are highlighted with coloured borders and labels. **d**, The preference of protein complexes for H3 or H4 acetylation. Markers indicate the median effect of the H3ac versus the H4ac feature across all complex subunits with protein response measurements (the number of measurements per complex/feature is shown in Supplementary Fig. 1). The error bars represent the empirical 95% confidence interval (CI) of this median effect estimated from 100,000 random samples of subunit effects, accounting for their variance. The coloured bars highlight the difference between these median estimates for H3ac and H4ac. Complexes are ordered from H3ac to H4ac preference. The asterisks denote estimates for exclusive complex subunits.

**Extended Data Fig. 6:**
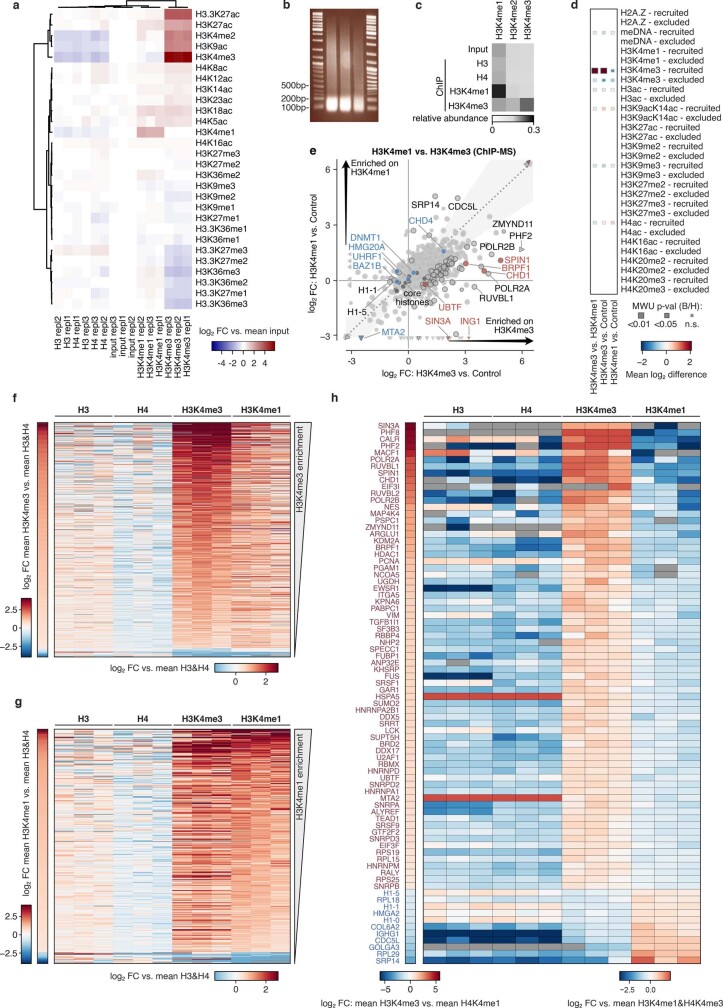
ChIP-MS profiling of H3K4me1- and H3K4me3-associated chromatin proteins in IMR-90 cells. **a**, Clustered heatmap of log_2_ FC (fold change) values for the relative abundances of histone PTMs measured by LC-MS in methyl state-specific anti-H3K4me1 and anti-H3K4me3 ChIP experiments and control anti-H3 and anti-H4 nucleosome purifications (each performed in n = 3 biologically independent experiments) as compared to the mean of three input chromatin samples (see **b**). Note, that in order to improve the identification of H3K4 methylation state-specific chromatin-associated proteins, the anti-H3 and anti-H4 control ChIPs were performed using the same inputs that had first been used for H3K4me1 and H3K4me3 ChIPs, and were therefore partially depleted in these modifications and proteins associated with H3K4 methylated nucleosomes (see online methods for details). **b**, Ethidium bromide-stained agarose gel showing DNA isolated from n = 3 independently prepared dual-crosslinked IMR-90 chromatin samples solubilised and fragmented by sonication that were used as inputs for the three replicates of the anti-H3K4me1 and anti-H3K4me3 ChIP-MS experiments shown in panels **a** and **e-h**. Note that most DNA fragments range between 100-200 bp in size, corresponding to mono-nucleosomes. **c**, Mean relative abundances of different H3K4 methylation states in ChIP purifications and in the input chromatin from n = 3 independent experiments (see panels **a** and **b**). **d**, Comparison of ChIP-MS profiling of H3K4me1- and H3K4me3-associated proteins with MARCS feature effect estimates. The heatmap depicts the log_2_ difference in the imputed ChIP-MS log_2_ FC estimates (H3K4me3 vs. H3K4me1, H3K4me3 vs. control, or H3K4me1 vs. control) for proteins strongly recruited or excluded by a given MARCS feature to the imputed log_2_ FC estimates of all other proteins detected in both MARCS and ChIP-MS data. Note that proteins that are predicted to be recruited by H3K4me3 in MARCS are statistically enriched in H3K4me3 but not in H3K4me1 ChIP purifications. **e**, ChIP-MS analysis of proteins associated with H3K4me1- and H3K4me3-modified chromatin in crosslinked IMR-90 cells. Log_2_ FC in normalized protein abundances over mean H3 and H4 ChIP controls for H3K4me3 and H3K4me1 ChIPs (n = 3 biologically independent experiments each) are plotted on the x and y axes, respectively. Differentially abundant proteins (H3K4me1 vs. H3K4me3; limma, two-sided, Benjamini/Hochberg-adjusted FDR ≤ 0.05) are circled with grey border. The area ± 0.2 radians away from the dotted x = y line is shaded in grey. Proteins strongly recruited or excluded by H3K4me3 in MARCS data are displayed in red and blue respectively, and core histone proteins (normalization controls) in dark grey. Smaller datapoints indicate response estimates based on single data points. Triangles indicate points outside of the data axes. Note that the vast majority of differentially abundant proteins preferentially associate with H3K4me3-modified chromatin while only few proteins show preferential association with H3K4me1. **f**, Heatmap of log_2_ FC in the normalized protein abundances for the specified ChIP-MS experiments as compared to the mean of the control anti-H3 and anti-H4 ChIPs, ordered from most to least enriched in the H3K4me3 ChIP. The column on the left shows the log_2_ FC in the mean normalized protein abundances in H3K4me3 vs. mean anti-H3 and anti-H4 control ChIPs. **g**, Heatmap of log_2_ FC in the normalized protein abundances for the specified ChIP-MS experiments as compared to the mean of the control anti-H3 and anti-H4 ChIPs, ordered from most to least enriched in H3K4me1 ChIP. The column on the left shows the log_2_ FC in the mean normalized protein abundances in H3K4me1 vs. mean anti-H3 and anti-H4 control ChIPs. **h**, Heatmap depicting differentially abundant proteins (H3K4me3 ChIP vs. H3K4me1 ChIP) ordered by the log_2_(H3K4me3/H3K4me1) FC estimate from the most enriched in H3K4me3 ChIP to most enriched in H3K4me1 ChIP. Proteins that are more abundant in the H3K4me3 ChIP as compared to the H3K4me1 ChIP are marked in red, while proteins more abundant in H3K4me1 ChIP are marked in blue (left colour axis). Log_2_ FC in the normalized protein abundance for the specified ChIP experiments vs. mean of H3K4me1 and H4K4me3 ChIPs is plotted in the heatmap on the right. Note that the vast majority of differentially abundant proteins are specifically enriched in the H3K4me3 ChIP while only few proteins are enriched in the H3K4me1 ChIP.

Another notable result from the feature effect analysis was the differential binding of proteins to H3K4 methylations (Fig. 3a). For the promoter mark H3K4me3, we identified 45 strongly recruited proteins (positive effect to log_2_[H/L ratio] ≥ 1 at a false-discovery rate (FDR) of 1%), including known H3K4me3 readers such as TFIID^31^ and PHF8^32^, and 31 strongly excluded proteins (Fig. 2b and Supplementary Table 3), such as polycomb repressive complex 2 (PRC2)^33^. By contrast, the enhancer mark H3K4me1 enriched only one protein, BRPF3 (Extended Data Fig. 3c). Consistent with these findings, our integrative ChIP–seq data analysis revealed no proteins showing strong association with H3K4me1, while many proteins preferentially localized to H3K4me3-marked genomic loci (Extended Data Fig. 4c,d). This was further supported by a label-free quantitative ChIP–MS analysis of H3K4me1- and H3K4me3-enriched mononucleosomes (Extended Data Fig. 6a–c). Although many proteins were significantly enriched in both H3K4me1 and H3K4me3 ChIPs compared with bulk nucleosome purifications, the vast majority of these proteins preferentially associated with H3K4me3- but not H3K4me1-modified chromatin (Extended Data Fig. 6d–h and Supplementary Table 6). This suggests the absence of a distinctive H3K4me1 interactome, supporting the notion that H3K4me1 is not a main driver of protein recruitment to enhancer chromatin states.

## MARCS recovers protein interaction networks

**Extended Data Fig. 7:**
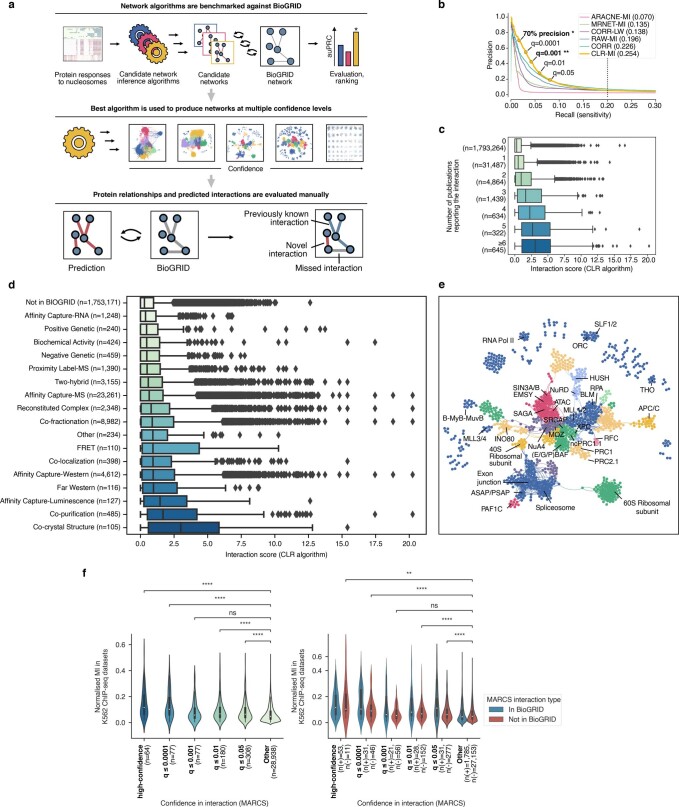
Network training procedure and inferred network. **a**, Schematic of the network inference process. A set of candidate protein interaction networks was generated using published network inference algorithms. The networks were evaluated against BioGRID^34^ as a reference database of known interactions. The best performing network algorithm was selected based on the highest partial area under PRC curve (auPRC, see Supplementary Information). Network estimates at different confidence levels were generated and investigated for chromatin interactions. **b**, Partial PRC curves of the estimated protein-protein interaction networks. Six different network algorithms were trained and tested against BioGRID as a reference set of known protein interactions. Performance of the network inference was benchmarked by scoring the number of recovered BioGRID interactions. As our experiment was not expected to recover the whole of BioGRID, the networks were evaluated by partial area under precision and recall curve (auPRC) at a 20% sensitivity threshold. At this threshold the CLR network algorithm, which uses mutual information (MI), produced the network with the highest area under the curve. Five parameter thresholds were selected to generate networks at increasing stringency, out of which q = 0.001 (marked ** in the plot) forms the basis of Fig. 3b and panel **e**, and a high-confidence network at 70% precision (marked *) is displayed in Extended Data Fig. 8. **c**, Estimated interaction scores broken down by number of publications reporting the interaction in BioGRID. Data is depicted as standard boxplots, with the boxes ranging from the first (Q1) to the third (Q3) quartile and the median (Q2) indicated. The lower whiskers are at the lowest datum above Q1 – 1.5 x (Q3 – Q1), and the upper whiskers at the highest datum below Q3 + 1.5 x (Q3 – Q1). Data beyond whiskers are considered outliers and plotted as individual data points. N indicates the number of pairwise interactions between proteins (i.e. potential edges in the network) reported in BioGRID in each of the different publication-count categories. Note that interactions reported in the literature more frequently receive higher median interaction scores (see also Supplementary Table 7). **d**, Estimated interaction scores broken down by experimental method by which they were identified. Data depicted as in panel **c**. N indicates the number of pairwise interactions between proteins (i.e. potential edges in the network) reported in BioGRID for each specified experimental method (see also Supplementary Table 7). **e**, Network generated from the SNAP binding data using the CLR algorithm at a stringency threshold of q = 0.001. Key chromatin regulatory complexes form clusters in the network, see also Supplementary Table 8. A zoomable version is provided in the MARCS online interface. **f**, Integrative analysis of the MARCS network protein-protein interaction (PPI) predictions and ENCODE ChIP-seq datasets for the K562 cell line. The predicted interactions of proteins within the MARCS PPI network for which ChIP-seq data was available were stratified into bins of increasing confidence (x axis). For each of the stratified interactions, the distribution of symmetrically normalized MI coefficient estimates (see online methods) are shown in the violin plots (Y axis). The boxplots inside the violins are depicted as in panel **c**, but without any outliers shown. N indicates the number of pairwise interactions between proteins in the different confidence categories, with n(+) in the right panel indicating predicted interactions reported in BioGRID (blue) and n(−) indicating predicted interactions not reported in BioGRID (red). Note that as the confidence from MARCS increases, the normalized MI estimate increases as well (left panel, q ≤ 0.05 vs. Other: p-value = 4.037 × 10^−7^/U-statistic = 5.229 × 10^6^, q ≤ 0.01 vs. Other: p-value = 4.167 × 10^−7^/U-statistic = 3.193 × 10^6^, q ≤ 0.001 vs. Other: p-value = 1.161 × 10^−1^/U-statistic =1.260 × 10^6^, q ≤ 0.0001 vs. Other: p-value = 3.932 × 10^−10^/U-statistic = 1.584 × 10^6^, high-confidence vs. Other: p-value = 7.875 × 10^−13^/U-statistic = 1.414 × 10^6^, Mann-Whitney U test, one-sided, Bonferroni-corrected), validating MARCS results. In addition to this, the similar conclusion holds when considering only the interactions that were not known at the network training time (right panel, red - ‘Not in BioGRID’ category, q ≤ 0.05 vs. Other: p-value = 3.868 × 10^−5^/U-statistic = 4.327 × 10^6^, q ≤ 0.01 vs. Other: p-value = 5.843 × 10^−5^/U-statistic 2.474 × 10^6^, q ≤ 0.001 vs. Other: p-value = 7.096 × 10^−1^/U-statistic = 8.232 × 10^5^, q ≤ 0.0001 vs. Other: p-value = 9.474 × 10^−5^/U-statistic = 8.437 × 10^5^, high-confidence vs. Other: p-value = 2.621 × 10^−3^/U-statistic = 2.346 × 10^5^, Mann-Whitney U test, one-sided, Bonferroni-corrected). In both panels ****, ***, **, * indicate p ≤ 0.0001, p ≤ 0.001, p ≤ 0.01, and p ≤ 0.05 (respectively), ns = not significant.

Closer analysis of binding profiles of protein complexes indicated that their subunits showed highly similar binding behaviours (for example, the H2A.Z-responsive INO80, SRCAP and NSL complexes; Extended Data Fig. 5d), underscoring that their native compositions remained intact during the affinity purifications. This prompted us to reconstruct a network of proteins co-regulated by similar chromatin states and use this to predict protein–protein interactions. To this end, we trained and tested several network inference algorithms (Extended Data Fig. 7a) against BioGRID^34^. In this analysis, the context-likelihood of relatedness (CLR) algorithm^35,36^ performed best based on the highest area under the precision-recall curve (Extended Data Fig. 7b). CLR also scored interactions reported by multiple publications and validated by co-crystal structures and co-purifications highest (Extended Data Fig. 7c,d), confirming the reliability of the predicted network.

**Extended Data Fig. 8:**
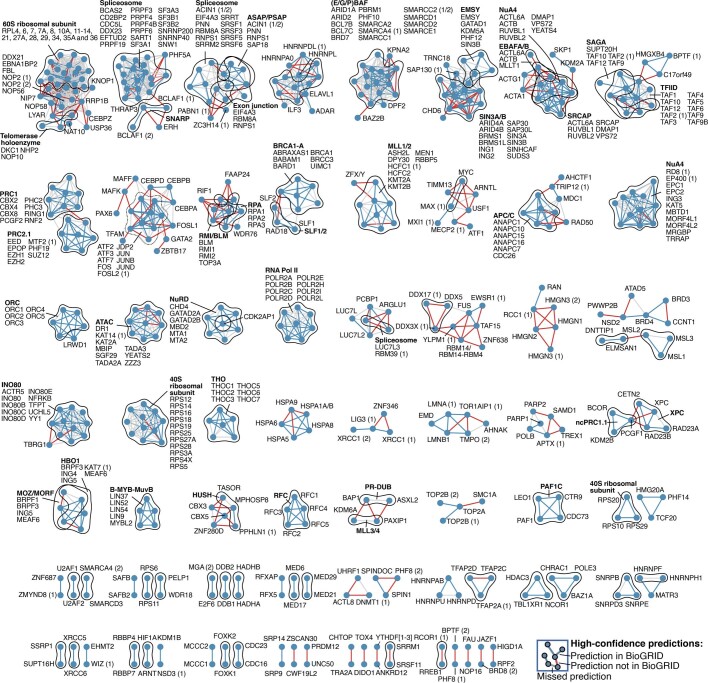
High-confidence protein interaction predictions from MARCS data. A plot of high-confidence protein interactions predicted by our network using the CLR-MI algorithm at an increased stringency of 70% precision. In this subnetwork, 30% of predicted edges were not previously deposited to BioGRID and therefore constitute potential novel interactions. Since increased precision is met with reduced recall, the network was augmented with edges linking interactions deposited to BioGRID but not recovered at this threshold (i.e. false negatives). Blue edges highlight predicted and known interactions reported in BioGRID. Potentially novel interactions predicted by our network that are not in BioGRID (at the time of network training) are highlighted in red, while interactions reported in BioGRID that did not pass the high-confidence threshold (i.e. false negatives) are indicated by grey lines. These annotations were added in the interest of organizing the network into connected sub-modules so the context of predictions can be interpreted more readily. Subunits of known protein complexes are circled and annotated with the complex name in bold letters.

Within the resulting network (Supplementary Table 7), key chromatin regulatory complexes formed clusters (Extended Data Fig. 7e) that, at increased stringencies, resolved into separate complexes and high-confidence binary interactions (Extended Data Fig. 8). Importantly, the normalized mutual information (MI) estimates between pairs of proteins in our integrative ChIP–seq analysis increased in line with increasing confidence of the predicted interactions (Extended Data Fig. 7f), indicating that the CLR-predicted network correctly enriches in vivo chromatin interactions. We leverage the identified local protein interactions to implement similarity predictions in the MARCS resource and augment these with a curated list of protein complexes (Supplementary Table 8), incorporating information from other resources such as EpiFactors^37^ and the Complex Portal^38^.

The CLR algorithm, being based on MI, treats mutually exclusive interactions similarly to correlated ones. Overlaying the chromatin feature effect estimates for each protein onto the network reveals how their arrangement into tight subnetworks is driven by the chromatin modification responses (Fig. 3b). Among other regulations, these data reveal differential binding of many factors to H3 and H4 acetylations, as different subnetworks show distinct binding responses to H3K27ac, H4K16ac, and the combined H3K9acK14ac, H3ac and H4ac features, suggesting a finely orchestrated regulation of active chromatin states by differential acetylation. Whereas, for example, the CHRAC chromatin remodelling complex shows preferential binding to H4ac, BAF (SWI/SNF) remodellers show a strong preference for H3ac (Fig. 3c,d), mainly driven by H3K9acK14ac (Fig. 3b). Furthermore, while many proteins respond to multiple acetylations in the H3 and H4 tails, only few factors respond to H3K27ac or H4K16ac alone (Fig. 3b). This breakdown of the SNAP data into local interaction networks of co-regulated proteins and their responses to specific chromatin features provides important insights into how chromatin states are decoded by chromatin readers.

## Modifications and linkers act independently

**Fig. 4:**
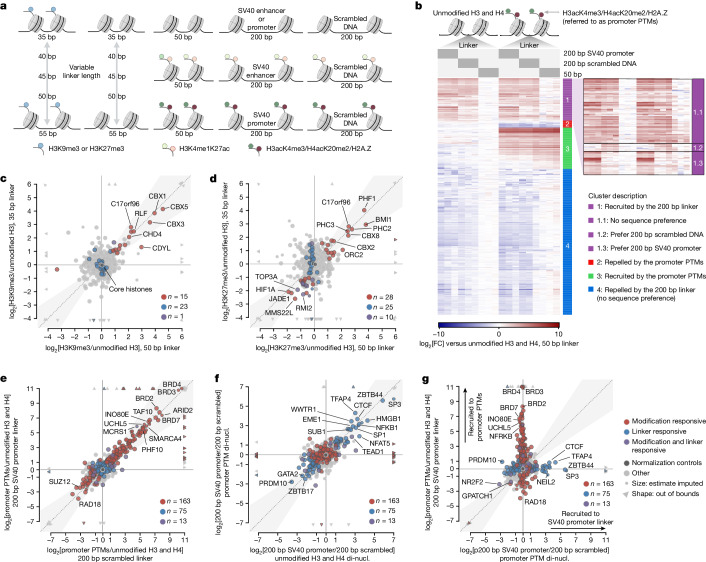
Nucleosomal modifications and linker DNA constitute orthogonal routes of protein engagement with chromatin. **a**, Schematic of dinucleosomes used in label-free MS-based pull-downs for evaluating the effect of linker DNA length and sequence on protein binding to active (right) and repressive (left) chromatin states. **b**, Clustered heat map depicting protein binding responses to dinucleosomes incorporating different combinations of 200 bp scrambled DNA or SV40 promoter sequence-based linkers and promoter PTMs (H3K4me3K9acK14acK18acK23acK27ac in combination with H4K5acK8acK12acK16acK20me2 and H2A.Z). Data are shown as the log_2_-transformed fold change (log_2_[FC]) in the normalized protein abundances compared with unmodified dinucleosomes with a 50 bp linker. **c**, Comparison of H3K9me3-binding responses on dinucleosomes with 35 bp and 50 bp linkers. Proteins responding to H3K9me3, linker length or both were determined using limma statistics and are highlighted in red, blue or purple, respectively. Only binding responses fulfilling the following two criteria are depicted: (1) log_2_[FC] > 1 or log_2_[FC] < −1 compared with unmodified dinucleosomes with 50 bp linker; (2) Benjamini–Hochberg-adjusted *P* ≤ 0.05. The *x* = *y* line indicates where binding responses to H3K9me3 dinucleosomes incorporating 35 bp and 50 bp linkers are identical. The grey area marks ±0.2 radians away from the *x* = *y* line. Core histones (normalization controls) are indicated in dark grey. The smaller datapoints indicate response estimates based on single data points. The triangles indicate points outside the data axes. **d**, Comparison of H3K27me3-binding responses on dinucleosomes with 35 bp and 50 bp linkers. Data representation in **d**–**g** is as described in **c**. **e**, Comparison of protein binding responses to promoter PTMs on dinucleosomes with 200 bp scrambled DNA and SV40-promoter-sequence-based linkers. **f**, Comparison of sequence-specific protein binding responses to the SV40 promoter linker in unmodified dinucleosomes (di-nucl.) and dinucleosomes decorated with promoter PTMs. **g**, Comparison of protein binding responses to SV40 promoter linker and promoter PTMs.

**Extended Data Fig. 9:**
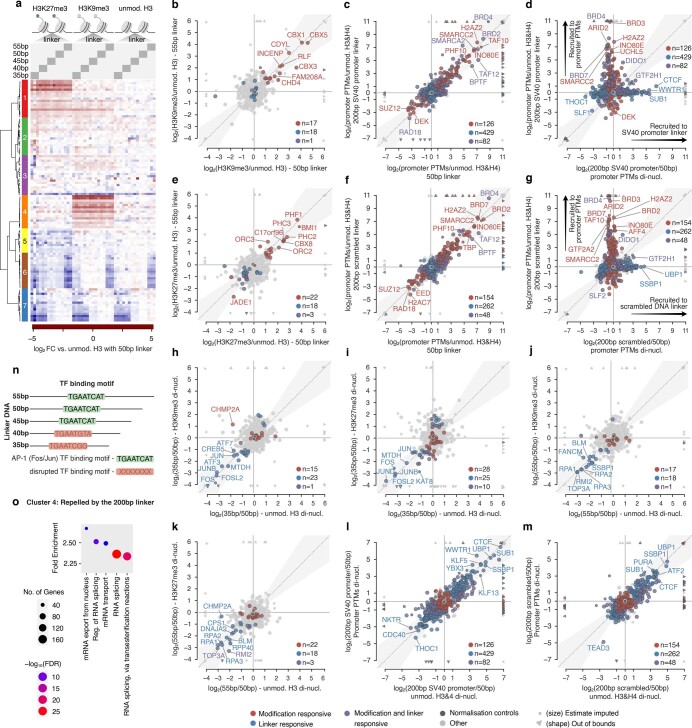
Probing the effect of di-nucleosome linker DNA on protein engagement with heterochromatin and promoter chromatin states. **a**, Clustered heatmap depicting protein binding responses to di-nucleosomes incorporating different combinations of H3K9me3 or H3K27me3 and linker lengths ranging from 35 bp to 55 bp with 5 bp increments. Data shown as log_2_ FC in the normalized protein abundances compared to unmodified di-nucleosomes with 50 bp control linker. Clusters 1 and 4 mark the H3K27me3- and H3K9me3-responsive proteins, respectively; these proteins are insensitive to variations in the linker length (see panels **b** and **e**). Clusters 6 and 7 mark proteins that respond with diminished binding to variations of the linker, independent of the modifications on the flanking nucleosomes. See also panels **h-k** and **n**. **b**, Comparison of protein binding responses to H3K9me3 on di-nucleosomes with 50 bp and 55 bp linkers. Data representation in **b-m** as in Fig. 4c. **c**, Comparison of protein binding responses to promoter PTMs present on di-nucleosomes with 50 bp and 200 bp SV40 promoter sequence-based linkers. **d**, Comparison of protein binding responses to promoter PTMs and the 200 bp SV40 promoter linker. Note that the binding responses mediated by promoter PTMs and the 200 bp SV40 promoter linker are largely independent of each other, as most proteins responding to one of the two features do not respond to the other and vice versa. **e**, Comparison of protein binding responses to H3K27me3 on di-nucleosomes with 50 bp and 55 bp linkers. **f**, Comparison of protein binding responses to promoter PTMs present on di-nucleosomes with 50 bp and 200 bp scrambled DNA sequence-based linkers. **g**, Comparison of protein binding responses to promoter PTMs and the 200 bp scrambled DNA linker. Note that the binding responses mediated by promoter PTMs and the 200 bp scrambled DNA linker are largely independent of each other, as most proteins responding to one of the two features do not respond to the other and vice versa. **h**, Comparison of protein binding responses to the 35 bp linker in relation to the 50 bp linker in unmodified and H3K9me3-decorated di-nucleosomes. See also panel **n**. **i**, Comparison of protein binding responses to the 35 bp linker in relation to the 50 bp linker in unmodified and H3K27me3-decorated di-nucleosomes. See also panel **n**. **j**, Comparison of protein binding responses to the 55 bp linker in relation to the 50 bp linker in unmodified and H3K9me3-decorated di-nucleosomes. **k**, Comparison of protein binding responses to the 55 bp linker in relation to the 50 bp linker in unmodified and H3K27me3-decorated di-nucleosomes. **l**, Comparison of protein binding responses to the 200 bp SV40 promoter linker in relation to the 50 bp linker in unmodified di-nucleosomes and di-nucleosomes decorated with promoter PTMs. **m**, Comparison of protein binding responses to the 200 bp scrambled DNA linker in relation to the 50 bp linker in unmodified di-nucleosomes and di-nucleosomes decorated with promoter PTMs. **n**, Schematic representation of the di-nucleosome linker DNAs ranging from 35 bp to 55 bp that were used in Fig. 4c,d and panels **b**, **e**, and **h**-**k**. Note that due to the design of the 5 bp increments in the linker DNA the 55 bp, 50 bp, and 45 bp linkers contain an AP-1 binding motif that is disrupted in the 40 bp and 35 bp linkers (see also Supplementary Information), resulting in impaired binding of several AP-1 family TFs, including FOS, FOSL2, JUN and JUNB to di-nucleosomes with the 40 bp and 35 bp linker DNAs. These are identified as cluster 7 in panel **a** and in the comparisons in panels **h-i**. **o**, Gene ontology enrichment analysis of proteins showing impaired binding to di-nucleosomes incorporating 200 bp long linker DNAs (200 bp SV40 promoter and scrambled DNA linkers). See cluster 4 in Fig. 4b.

**Extended Data Fig. 10:**
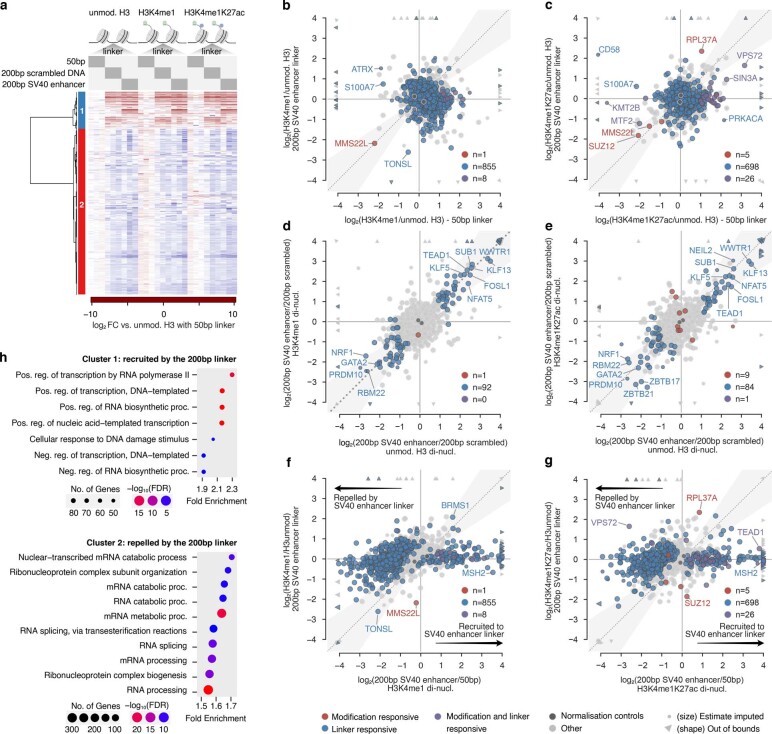
Probing the effect of linker DNA on protein engagement with the enhancer chromatin state. **a**, Clustered heatmap depicting protein binding responses to di-nucleosomes incorporating different combinations of 200 bp scrambled DNA or SV40 enhancer sequence-based linkers and enhancer-associated PTMs (H3K4me1 and H3K4me1K27ac). Data shown as log_2_ FC in the normalized protein abundances compared to unmodified di-nucleosomes with a 50 bp linker. **b**, Comparison of protein binding responses to H3K4me1 on di-nucleosomes with 50 bp and 200 bp SV40 enhancer sequence-based linkers. Data representation in **b-g** as in Fig. 4c. Note that only one protein shows a statistically significant binding response to H3K4me1 regardless of which linker is used, indicating that H3K4me1 has limited regulatory potential even when placed on nucleosomes flanking an NDR containing an enhancer DNA sequence. **c**, Comparison of protein binding responses to H3K4me1K27ac on di-nucleosomes with 50 bp and 200 bp SV40 enhancer linkers. Note that binding of only few proteins is stimulated by H3K4me1K27ac regardless of which linker is used, indicating that H3K4me1K27ac has limited potential in mediating protein recruitment to chromatin even when placed on nucleosomes flanking an NDR containing an enhancer DNA sequence. **d**, Comparison of sequence-specific protein binding responses to the 200 bp SV40 enhancer linker in unmodified di-nucleosomes and di-nucleosome decorated with H3K4me1. **e**, Comparison of sequence-specific protein binding responses to the 200 bp SV40 enhancer linker in unmodified di-nucleosomes and di-nucleosome decorated with H3K4me1K27ac. **f**, Comparison of protein binding responses to 200 bp SV40 enhancer linker and H3K4me1. Note that proteins responsive to the SV40 enhancer linker show no major regulation by H3K4me1, and vice versa. **g**, Comparison of protein binding responses to 200 bp SV40 enhancer linker and H3K4me1K27ac. Note that proteins responsive to the SV40 enhancer linker show no major regulation by H3K4me1K27ac, and vice versa. **h**, Gene ontology enrichment analysis of proteins that show either enhanced or impaired binding to di-nucleosomes with the 200 bp linker (clusters 1 and 2 in panel **a**).

Apart from covalent modifications, characteristic features of chromatin states also include linker DNA length, typically ranging from 35–55 bp in most chromatin domains^39^ to over 200 bp in nucleosome-depleted regions (NDRs). To investigate the effects of linker DNA on chromatin recognition by nuclear proteins, we performed an additional set of affinity purifications using dinucleosomes incorporating different DNA linkers (Fig. 4a and Supplementary Information). Notably, the binding of heterochromatin as well as active promoter modification readers was generally not affected by variations in linker length nor linker sequence (Fig. 4b–e, Extended Data Fig. 9a–g and Supplementary Table 9), highlighting the robustness of the protein binding responses captured in MARCS. Likewise, the binding of sequence-specific transcription factors recognizing DNA motifs in the 200 bp long SV40 promoter linker was insensitive to the active promoter modifications on the adjacent nucleosomes (Fig. 4f,g and Extended Data Fig. 9d,g). Similarly, incorporating a 200 bp long SV40 enhancer linker had no prominent effect on H3K4me1 and H3K4me1K27ac enhancer state readout (Extended Data Fig. 10a–c and Supplementary Table 9), and transcription factor recognition of the SV40 enhancer sequence was not affected by the H3 modifications (Extended Data Fig. 10d,e). Nucleosomal modifications and DNA linkers therefore appear to act largely independently in recruiting proteins to chromatin. Notably, many proteins, including multiple spliceosome subunits, showed diminished binding when increasing the linker length from 50 to 200 bp, regardless of the linker sequence or modification status of the adjacent nucleosomes (Fig. 4b and Extended Data Figs. 9l,m,o and 10a,f–h), underscoring the regulatory potential of nucleosome spacing on chromatin engagement irrespective of the underlying modification landscape.

## Multivalent chromatin engagement by INO80

**Fig. 5:**
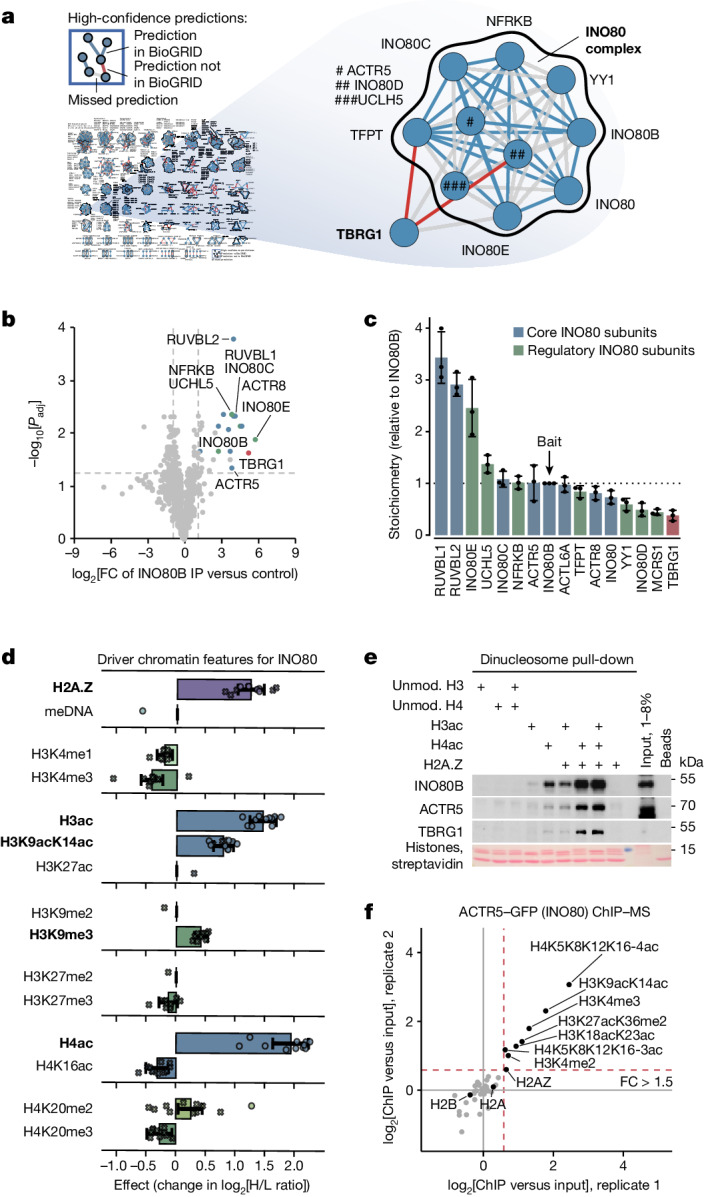
The INO80 complex recognizes a multivalent nucleosome-modification signature. **a**, CLR-predicted TBRG1–INO80 interaction. TBRG1–INO80 interactions were reported in several screens^48–50^ and deposited at BioGRID but never validated. **b**, TBRG1 interacts with INO80. Volcano plot of proteins that are significantly enriched (*t*-test, two-sided, Benjamini–Hochberg-adjusted FDR ≤ 0.05) in *n* = 3 biologically independent INO80B-V5 immunoprecipitations (Extended Data Fig. 5h) followed by label-free MS. **c**, Composition of the INO80 complex. The relative stoichiometries between TBRG1 and INO80 were calculated using quantitative MS data from the INO80B-V5 immunoprecipitation experiments shown in **b**. *n* = 3. Data are the mean ± s.d. of the stoichiometry values. **d**, Features driving the INO80 nucleosome-binding response. Individual effect estimates (change in log_2_[H/L ratio]) for INO80-exclusive subunits are shown as dots (estimate significantly non-zero, limma, two-sided, Benjamini–Hochberg-adjusted FDR ≤ 0.01) or crosses (estimate not statistically significant). The bars highlight the median effect across all complex subunits with protein response measurements (*n* = 11, except for DNA methylation, H3K27ac, H3K9me2 and H3K27me2, for which *n* = 1 and no estimate was derived). The error bars represent the empirical 95% CI of this median effect estimated from 100,000 random samples of subunit effects, accounting for their variance. The bold font indicates features with enrichments greater than expected by chance (CAMERA, Benjamini–Hochberg-adjusted FDR ≤ 0.01; Supplementary Table 8). **e**, Targeted dinucleosome pull-downs confirm INO80 binding to nucleosomes containing hyperacetylated H3 (H3ac), H4 (H4ac) and/or H2A.Z. Binding was detected by immunoblotting against INO80B and ACTR5. TBRG1 follows the INO80-binding pattern. The HeLa S3 cell nuclear extract used was a mixture of three independent preparations. Different amounts of the mixed extract were loaded as inputs for the different immunoblots. Experiments were independently repeated three times with similar results. Unmod., unmodified. **f**, Quantitative label-free LC–MS-based analysis of histone modifications and H2A.Z in mononucleosomes co-purified with ACTR5 from MNase-digested HeLa cell chromatin. The relative PTM or H2A.Z abundance over input chromatin is plotted as the log_2_[FC] for *n* = 2 independent biological experiments.

Our combined analyses can be used to identify chromatin binding behaviours and nuclear regulators with unknown functions. As a proof of principle, we selected INO80, an ATP-dependent nucleosome remodeller and exchange factor for the histone variant H2A.Z that is involved in transcription, replication and DNA repair^28^, for which several interesting observations emerged from our data (Extended Data Fig. 5d). First, our high-confidence CLR network predicted an interaction with transforming growth factor beta regulator 1 (TBRG1), a putative tumour suppressor and p53 activator^40^ (Fig. 5a and Extended Data Fig. 8). Consequently, we were able to co-purify TBRG1 together with INO80 in co-immunoprecipitation (co-IP) experiments from INO80B-V5 knock-in cell lines (Fig. 5b and Extended Data Fig. 5e–h). Label-free MS-based estimation of the TBRG1:INO80B ratio indicated that TBRG1 is present in the complex at substoichiometric levels comparable to the regulatory subunits MCRS1, INO80D and YY1 (Fig. 5c).

Second, while the INO80 complex was unresponsive to variations in the linker DNA (Fig. 4e–g and Extended Data Fig. 9c,d,f,g), our feature effect estimates predicted binding to a multivalent nucleosomal modification signature consisting of acetylations in the H3 and H4 N-terminal tails and the histone variant H2A.Z (Fig. 5d and Extended Data Fig. 5b,c). Confirming our prediction, we found in targeted pull-downs (Fig. 5e) that H3ac had a small positive effect on INO80 recruitment, which was more pronounced in the case of H4ac. Notably, while no effect of H2A.Z alone was detectable by western blotting, the presence of H2A.Z greatly enhanced INO80 binding when combined with H4ac, and to a lesser extent with H3ac (Fig. 5e). Consistent with the in vitro results, mononucleosomes co-purified with INO80 from micrococcal nuclease (MNase)-digested HeLa chromatin through the subunit ACTR5 were enriched in H4ac and H3ac as well as H2A.Z (Fig. 5f and Extended Data Fig. 5i–k). These results confirm that the INO80 remodelling complex indeed binds to nucleosomes decorated by the predicted multivalent chromatin modification signature in human cells and suggest a role of histone acetylation and H2A.Z in stimulating INO80 recruitment to specific genomic loci (Extended Data Fig. 5l).

These independent experimental validations highlight the reliability of our analyses and predictions, and underscore the value of our data to identify previously undescribed protein interactions and complex binding events involving the concerted interplay between multiple chromatin modification features.

## Discussion

Here we have combined large-scale quantitative nucleosome affinity purification approaches and computational analysis methods to understand how chromatin states are read and interpreted by nuclear machineries. Our approach has enabled us to delineate direct effects of composite modification signatures of promoter, enhancer and heterochromatin states on chromatin engagement by several hundred chromatin readers and to uncover interconnected networks of nuclear proteins targeting similar chromatin states. Deconvoluting the responses of chromatin factors to 15 different modification features unravels how complex modification signatures are sensed by chromatin-binding proteins. Combining these responses to individual modification features into modification response profiles, akin to DNA-binding-motif logos of transcription factors^41^, enables the comprehensive prediction of chromatin regulators that recognize complex modification patterns. Similarly, it enables the systematic identification of nucleosomal features modulating the binding of various nuclear proteins to their genomic target loci. Predicted responses to multiple features point towards a synergistic interplay between the components, as we show for the INO80 remodeller (Fig. 5e,f).

While an interplay between distinct nucleosomal modifications is clearly visible for many proteins, it generally seems not to involve linker DNA as we observe no apparent synergy even between active modifications and NDRs often coupled in vivo. However, this might reflect the static nature of the interactions in our pull-downs, in which the absence of ATP and the presence of HDAC inhibitors prevent enzymatic activities that are known to be involved in highly dynamic regulatory circuits, such as nucleosome remodelling and rapid histone acetylation turnover. In the case of multistep enzymatic processes, such as chromatin remodelling by INO80, the reported interactions might therefore reflect particular intermediate states of a dynamic reaction cycle, probably representing one of the first engagement steps of the complex with chromatin. Likewise, although we saw no prominent effects of different linkers on protein binding to modifications and vice versa, a dynamic interplay between the two cannot be excluded. The testable transcription-factor-binding sites in the linkers were located distant from the nucleosome-bound DNA regions, and histone modifications were unlikely to directly modulate their accessibility. In the presence of ATP, nucleosomal modifications can potentially modulate chromatin remodelling activities that could in turn expose nucleosomal DNA sequences, therefore facilitating, for example, the binding of pioneer transcription factors^42^ thereby enabling the establishment or maintenance of NDRs.

Notably, modifications that are characteristic of distinct chromatin states vary greatly in their regulatory potential, as promoter-associated H3K4me3 and hyperacetylated H3 and H4 tails affect the binding of many nuclear factors, while enhancer-associated H3K4me1 and H3K27ac appear largely inert in targeting proteins to chromatin. Consistent with previous findings^43,44^, this suggests that modifications found at enhancers may act, for example, by preventing the binding of repressive factors to the underlying regulatory loci^45^, rather than by directly recruiting proteins.

Our study unifies two complementary views of chromatin—the modification-centric view that defines chromatin states based on chromatin marks^1,2^, and the protein-centric view that defines the chromatin states by their protein constituents^46^. By combining both aspects, our experiments reveal major principles of how complex modification patterns define and regulate functional chromatin states. Our data are easily accessible through the interactive online resource MARCS (https://marcs.helmholtz-munich.de) with the aim to serve as a platform for both hypothesis generation and validation, and thereby act as a catalyst for future chromatin research. We encourage researchers to thoroughly explore the data as there are many discoveries to be made.

## Methods

### Experimental procedures

#### Preparation of recombinant canonical histones

Recombinant human canonical histone proteins were expressed in *Escherichia coli* BL21(DE3)-CodonPlus-RIL cells (Agilent Technologies) from pET21b(+) (Novagen) vectors and purified by denaturing gel filtration and ion-exchange chromatography as previously described^25,51^.

#### Preparation of recombinant histone H2A.Z

A codon-optimized sequence encoding human H2A.Z (H2AFZ, UniProtKB: P0C0S5) was purchased from GenScript and cloned into the NdeI/XhoI sites of the pET24a(+) vector (Novagen). H2A.Z was then expressed in *E. coli* BL21(DE3)-CodonPlus-RIL cells (Agilent Technologies) and purified as previously described for canonical H2A^25^.

#### Preparation of truncated histones for native chemical ligations

Truncated human H3Δ1–31T32C protein for ligations of modified histone H3 was expressed in *E. coli* BL21(DE3)-CodonPlus-RIL cells (Agilent Technologies) and purified as previously described^52^. Truncated human H4Δ1–28I29C protein for ligations of modified histone H4 was expressed from pET24b(+) vectors (Novagen) in *E. coli* BL21(DE3)-CodonPlus-RIL cells (Agilent Technologies). The insoluble protein was extracted from inclusion bodies with unfolding buffer (20 mM Tris (pH 7.5), 7 M guanidine hydrochloride, and 100 mM dithiothreitol (DTT)) for 1 h at room temperature, and the cleared supernatant was loaded onto a Sephacryl S-200 gel filtration column (Cytiva) in SAU-1000 buffer (20 mM sodium acetate (pH 5.2), 7 M urea, 1 M NaCl, and 1 mM ethylenediaminetetraacetic acid (EDTA)) without any reducing agents. Positive fractions were combined and further purified by reversed-phase chromatography. Truncated H3Δ1–31T32C was purified over a Resource RPC column (Cytiva) using a gradient of 0–65% B (buffer A: 0.1% trifluoroacetic acid in water; B: 90% acetonitrile, 0.1% trifluoroacetic acid) over 20 column volumes. Truncated H4Δ1–28I29C was purified over a PerkinElmer Aquapore RP-300 (C8) column (250 mm × 4.6 mm inner diameter) using a gradient of 0–65% B (buffer A: 0.1% trifluoroacetic acid in water; B: 90% acetonitrile, 0.1% trifluoroacetic acid) over 20 column volumes. The fractions containing pure H3Δ1–31T32C or H4Δ1–28I29C were pooled and lyophilized.

#### Preparation of modified histone H3 and histone H4 by native chemical ligation

For the preparation of modified histone H3, N-terminal H3 peptides (amino acids 1–31) were ligated to truncated H3Δ1–31T32C and, for the preparation of modified histone H4, N-terminal H4 peptides (amino acids 1–28) were ligated to truncated H4Δ1–28I29C using native chemical ligation. All peptides contained a C-terminal benzyl thioester. All histone H4 peptides were N-terminally acetylated. Ligations were performed in 550 μl of degassed ligation buffer (200 mM KPO_4_, 2 mM EDTA, 6 M guanidine hydrochloride) containing 1 mg of modified/unmodified histone tail thioester peptide (purchased from Cambridge Peptides or Almac Sciences), 4 mg of truncated histone, 20 mg 4-mercaptophenylacetic acid and 25 mg Tris(2-carboxyethyl)phosphine as reducing agent at a pH of 7.5. The reactions were incubated overnight at 40 °C and quenched by addition of 60 μl 1 M DTT and 700 μl 0.5% acetic acid. After precipitation clearance by centrifugation, the ligation reactions were directly loaded and purified onto a reversed-phase chromatography column (PerkinElmer Aquapore RP-300 (C8) 250 mm × 4.6 mm inner diameter). Modified histone H3 was purified using a gradient of 45–55% B (buffer A: 0.1% trifluoroacetic acid in water; B: 90% acetonitrile, 0.1% trifluoroacetic acid) over 10 column volumes. Modified histone H4 was purified using a gradient of 35–45% B (buffer A: 0.1% trifluoroacetic acid in water; B: 90% acetonitrile, 0.1% trifluoroacetic acid) over 10 column volumes. Positive fractions containing ligated full-length histone H3 or histone H4 were then combined and lyophilized.

#### Nucleosome assembly

Histone octamers were refolded from the purified histones and assembled into nucleosomes with biotinylated DNA through salt deposition dialysis as previously described^25,51^. Biotinylated nucleosomal DNAs containing either one (mononucleosomes) or two 601 nucleosome-positioning sequences^47^ separated by a 50-base-pair (bp) linker (dinucleosomes), or four 601 nucleosome-positioning sequences (tetranucleosomes), were prepared as described previously^25^. CpG-methylated DNA was prepared using the M.SssI methyltransferase and complete methylation was confirmed by restriction digest (Supplementary Information). Dinucleosomes and tetranucleosomes were assembled in the presence of mouse mammary tumour virus A (MMTVA) competitor DNA (prepared in the same way as 601 DNA) and a slight excess of octamers as described for longer chromatin arrays to ensure saturation of the 601 repeats^53^. The reconstituted nucleosomes were then immobilized on streptavidin Sepharose High Performance beads (Cytiva) through the biotinylated DNA, washed to remove MMTVA competitor DNA and MMTVA nucleosomes (in the case of dinucleosomes and tetranucleosomes), and directly used for SILAC or label-free nucleosome affinity purifications. Correct assembly and immobilization of nucleosomes was verified by native polyacrylamide gel electrophoresis (Supplementary Information). Nucleosomes for pull-downs in which only modifications on histone H3 were tested were assembled with octamers containing recombinant histone H4 purified from *E. coli* instead of ligated H4. Likewise, nucleosomes for pull-downs in which only modifications on histone H4 were tested contained recombinant H3 and not ligated histone H3. Matched unmodified control nucleosomes were assembled with unmodified ligated H3 and recombinant H4, or recombinant H3 and unmodified ligated H4 accordingly. Nucleosomes containing only CpG methylation (H27M) were assembled with ligated unmodified H3 and recombinant H4, and nucleosomes containing only H2A.Z (H36) and no other modifications were assembled with recombinant (and therefore unmodified) H3 and H4 produced in *E. coli*.

#### Generation of 601 dinucleosomes incorporating different linker DNAs

Plasmid constructs for the preparation of biotinylated 601 dinucleosome DNAs containing different linker lengths (35 bp, 40 bp, 45 bp, 50 bp and 55 bp linkers) between the two 601 nucleosome-positioning sequences were generated by annealing forward and reverse primers of corresponding length and ligating them into pUC19-di601_NcoI/NheI_5xGal4 (pTB891, gene synthesis by Genscript) digested with NcoI and NheI restriction enzymes (Thermo Fisher Scientific), thereby exchanging the ‘5×Gal4 linker’ against the different linker fragments. Plasmid constructs for the preparation of biotinylated 601 dinucleosome DNAs containing 200 bp linkers consisting of either the SV40 enhancer or the SV40 promoter were generated by PCR amplification of the SV40 enhancer and promoter sequences from pGL3-control (Promega) and cloning the resulting fragments into the vector backbone of pUC19-di601_NcoI/NheI_5xGal4 through NcoI and NheI, thereby exchanging the ‘5×Gal4 linker’ against the 200 bp SV40 enhancer or promoter sequences. For all of the constructs, the dinucleosome sequences were then amplified from one copy to eight copies per plasmid as described previously^25,51^.

The biotinylated 601 dinucleosome DNAs containing 200 bp linkers with randomized DNA sequences were generated from a library of single-stranded 200 bp scrambled linker oligonucleotides (custom synthesis by Biolegio) containing 192 bp of randomized DNA sequence flanked by 5′ NcoI and 3′ NheI restriction sites and 5′ bGHR and 3′ pCIfor primer-binding sites. The single-stranded oligo was converted to double-stranded DNA by annealing it to the pCIfor primer (Sigma-Aldrich) and performing a primer extension of pCIfor. The primer extensions were performed using Taq DNA polymerase in a 96-well plate format with 96 × 50 µl reactions. Each 50 µl reaction contained 1 µg of the 200 bp scrambled linker oligonucleotide (250 nM), 340 ng pCIfor primer (1 µM, fourfold molar excess over the 200 bp scrambled linker oligonucleotide), 200 µM dNTPs and 2.5 U Taq polymerase (New England Biolabs) in 1× ThermoPol buffer (New England Biolabs). Using a thermocycler, the oligonucleotides were denatured for 5 min at 95 °C, annealed for 1 min at 58 °C and the primer extension reaction was then allowed to proceed for 5 min at 68 °C. The reactions were pooled and the remaining single-stranded DNA was removed by direct addition of 2,000 U of exonuclease I (New England Biolabs) per ml reaction volume and incubation for 30 min at 37 °C. The resulting double-stranded DNA was purified using the QIAquick PCR purification kit (Qiagen) according to the manufacturer’s instructions (20× columns, total yield of 75 µg in 1 ml buffer EB). The double-stranded 200 bp scrambled linker DNAs were digested with NcoI and NheI (Thermo Fisher Scientific) using 5 µl of FastDigest enzyme per µg DNA, concentrated using the QIAquick PCR purification kit (10× columns, total elution volume of 500 µl buffer EB) and separated by 2.5% agarose gel electrophoresis. The 200 bp band containing the scrambled linker fragments was excised from the gel and purified using the QIAquick gel extraction kit (Qiagen) according to the manufacturer’s instructions (eight columns, total yield of 11.64 µg in 300 µl buffer EB). The purified NcoI/NheI-digested 200 bp scrambled linker fragments were subsequently ligated into the NcoI/NheI-digested, dephosphorylated (Quick CIP, New England Biolabs) and agarose-gel-purified vector backbone of pUC19-di601_NcoI/NheI_5×Gal4, thereby exchanging the ‘5×Gal4 linker’ against the library of 200 bp scrambled linker fragments. Ligations were assembled using 50 µg of NcoI/NheI-linearized pUC19-di601 vector backbone, 11.64 µg of NcoI/NheI-digested 200 bp scrambled linker inserts (approximately 3.5-fold molar excess of inserts over the 3 kb vector backbone) and 200 µl (400,000 cohesive end units) of T4 DNA Ligase (New England Biolabs) in a total volume of 4 ml of 1× T4 DNA ligase reaction buffer, and incubated overnight at 16 °C. After the ligation, ATP was added to the reaction to a final concentration of 1 mM and unligated linear DNA was digested by addition of 1,000 U of exonuclease V (New England Biolabs) and incubation for 50 min at 37 °C. Circular plasmid DNA that was protected from the exonuclease V digestion was then purified and concentrated using the QIAquick PCR purification kit (10 columns, elution in 30 µl buffer EB per column). The total yield of ligated circular plasmid DNA was 6.5 µg in 280 µl. The ligated plasmids represent a library of pUC19 vectors in which each vector contains one copy of a 601 dinucleosome DNA each incorporating a different 200 bp linker of random sequence between the two 601 nucleosome-positioning sequences. The plasmid library was amplified by electroporation into 10-beta electrocompetent *E. coli* cells (New England Biolabs) according to the manufacturer’s instructions using 2 µl (47 ng) of library DNA and 25 µl of competent cells per electroporation. Cells were recovered in 1 ml of outgrowth medium and selected on 24.5 cm^2^ BioAssay LB_Amp_-agar plates (Corning). Serial dilutions were plated to determine the transformation efficiency and complexity of the library. In total, >10^8^ independent clones were obtained from 24 electroporations. The colonies were gently scraped off the plates in liquid LB medium and plasmid DNA was isolated using the NucleoBond PC 10000 Giga-prep kit (Macherey-Nagel). The total yield of plasmid DNA from 24 plates was 16 mg. In total, 20 clones were picked from a high-dilution plate and sequenced to verify the correct length and random composition of the 200 bp linker sequences.

For preparing the different biotinylated dinucleosome DNAs the pUC19 601 dinucleosome plasmid constructs were first digested with EcoRV, ethanol-precipitated and then further digested with EcoRI (New England Biolabs) to liberate the dinucleosome DNAs. After another ethanol precipitation, the EcoRI overhangs were filled in with dATP and biotin-11-dUTP (Yorkshire Bioscience) using Klenow (3′→5′ exo^−^) polymerase (New England Biolabs). The biotinylated dinucleosome DNAs were again concentrated by ethanol precipitation, separated from the pUC19 vector DNA by preparative agarose gel electrophoresis and then purified from the excised gel slices using the NucleoSpin gel extraction Maxi kit (Macherey-Nagel). Biotinylation and the purity of the dinucleosome DNAs were verified by depletion with streptavidin Sepharose High Performance beads (Cytiva) and agarose gel electrophoresis of the inputs and supernatants (Supplementary Information). Dinucleosomes were then assembled in the presence of MMTVA competitor DNA as described above.

#### Eukaryotic tissue culture

HeLa S3 cells (ATCC, CCL-2.2) cells were obtained from the Cancer Research UK Clare Hall Laboratories Cell Services Facility and maintained in suspension culture at 37 °C under 5% CO_2_ in RPMI 1640 medium. HeLa S3 cells were authenticated by morphology on the basis of their ability to grow both in suspension culture and as round spherical cells in adhesion culture. A HeLa Kyoto BAC cell line expressing the C-terminal localization and affinity purification (LAP)-tagged INO80 subunit ACTR5^48^ was a gift from M. Mann (Max Planck Institute of Biochemistry). Cells were cultured at 37 °C under 5% CO_2_ in Dulbecco’s modified Eagle’s medium (DMEM) containing 4.5 mg ml^−1^ glucose, 10% fetal calf serum, 1% penicillin–streptomycin and 1% l-glutamine and validated by immunoprecipitation and immunoblotting against the tagged ACTR5. MCF-7 cells (ATCC, HTB-22) were obtained from the Cell Services Facility of the IGBMC. Cells were cultured at 37 °C under 5% CO_2_ in DMEM containing 4.5 mg ml^−1^ glucose, 10% fetal calf serum, 1 mM sodium pyruvate, 1% penicillin–streptomycin and 1% l-glutamine and authenticated by morphology and by regularly testing the induction of oestrogen-responsive genes by quantitative PCR with gene-specific primers or global RNA-sequencing after 17β-estradiol treatment. IMR90 human fibroblasts were purchased directly from ATCC (CCL-186) and cultured at 37 °C under 5% CO_2_ in DMEM containing 4.5 mg ml^−1^ glucose, 10% fetal calf serum, 1 mM sodium pyruvate, 1% penicillin–streptomycin and 1% l-glutamine. Cells were authenticated by morphology and only maintained for a limited number of passages. All of the cell lines were tested and were mycoplasma free.

#### SNAP

SILAC-labelled nuclear extracts were prepared from HeLa S3 cells as previously described^25^. The isotopically light (R_0_K_0_) or heavy (R_10_K_8_) nuclear extracts were mixes of three independently prepared nuclear extracts. For each pull-down, nucleosomes corresponding to 12.5 μg of octamer were immobilized on 10 μl streptavidin Sepharose High Performance beads (Cytiva) in the final reconstitution buffer (10 mM Tris (pH 7.5), 250 mM KCl, 1 mM EDTA and 1 mM DTT; supplemented with 0.1% NP-40) and then rotated with 0.5 mg HeLa S3 SILAC-labelled nuclear extract in 1 ml of SNAP buffer (20 mM HEPES (pH 7.9), 150 mM NaCl, 0.2 mM EDTA, 10% glycerol) supplemented with 0.1% NP-40, 1 mM DTT and protease inhibitor cocktail (Roche) for 4 h at 4 °C. Nucleosome pull-downs with acetylated histones and the corresponding unmodified control pull-downs were supplemented with HDAC inhibitors (5 mM sodium butyrate (Sigma-Aldrich, B5887) and 250 nM TSA (Sigma-Aldrich, T1952)) to prevent removal of the acetyl modifications. After two washes with 1 ml SNAP buffer + 0.1% NP-40 and then two washes with 1 ml SNAP buffer without NP-40, the beads from both SILAC pull-downs (modified and unmodified control nucleosome) were pooled. The supernatant was completely removed, and bound proteins were eluted by on-bead digestion (see below).

#### Label-free nucleosome affinity purifications

Nuclear extracts were prepared from HeLa S3 cells as previously described^25^ except that cells were cultured with 10% regular fetal calf serum and no isotopically labelled amino acids were used. Unlabelled nuclear extracts were a mix of three independently prepared nuclear extracts. Nucleosome pull-downs were performed in the same manner as described above for SNAP, except for the bead washing and protein elution steps, which were performed as follows: after incubation with nuclear extracts, beads with immobilized nucleosomes were washed three times with 1 ml SNAP buffer + 0.1% NP-40, the supernatant was completely removed and bound proteins were eluted by boiling the beads in 50 µl Laemmli sample buffer containing 1% SDS at 95 °C for 5 min. A 20 µl protein aliquot was then digested with trypsin using a filter-aided sample preparation (FASP) protocol and analysed using liquid chromatography–mass spectrometry (LC–MS) as described below.

#### Cross-linking ChIP for MS analysis

IMR90 human fibroblasts were cultured as described above. Cells were washed three times with PBS and cross-linked on the plate with 1.25 µM ethylene glycol bis(succinimidyl succinate) (EGS) and 0.75 µM disuccinimidyl glutarate in PBS for 30 min at room temperature. After the first cross-linking reaction, cells were washed twice with PBS and cross-linked with 1% formaldehyde in PBS at room temperature for 10 min. Cross-linking reactions were quenched by the addition of glycine solution in PBS to a final concentration of 125 mM and incubation at room temperature for 5 min. Cells were then washed three times with ice-cold PBS, collected by scraping and pelleted by centrifugation (1,000*g*, 5 min, 4 °C). Cells were lysed in a hypotonic buffer (10 mM Tris (pH 7.6), 5 mM NaCl, 1.5 mM MgCl_2_) supplemented with 0.1% NP-40, protease inhibitor cocktail (Roche), 10 mM sodium butyrate and 1 mM DTT using a Dounce homogenizer as described previously^25^. Nuclei were pelleted by centrifugation (3,000*g*, 5 min, 4 °C), washed in hypotonic buffer supplemented with 300 mM NaCl and pelleted again (3,000*g*, 5 min, 4 °C). Nuclei were resuspended in nuclear lysis buffer (15 mM Tris (pH 7.6), 10% glycerol, 1% SDS) and incubated for 5 min on ice. Chromatin was pelleted by centrifugation (5,000*g*, 5 min, 4 °C), washed in chromatin wash buffer (15 mM Tris (pH 7.6), 300 mM NaCl, 1.5 mM MgCl_2_, 0.5% NP-40, 0.5% Triton X-100), pelleted again (5,000*g*, 5 min, 4 °C) and resuspended in ChIP buffer (20 mM Tris (pH 7.6), 150 mM NaCl, 2 mM EDTA, 1% Triton X-100, 0.01% SDS) supplemented with protease inhibitor cocktail (Roche) and 10 mM sodium butyrate. DNA was fragmented to an average size of 150–300 bp by sonication (Qsonica, Q800R2, 70% amp, 10 s off, 10 s on, 40 min active sonication time, 4 °C). Chromatin debris was pelleted by centrifugation (16,000*g*, 10 min, 4 °C). Then, 25 µl of supernatant was used for DNA purification to check the average DNA fragment size and another 25 µl supernatant aliquot was transferred to a fresh tube, de-cross-linked as described below, and stored at 4 °C until it was later used as the input sample for histone PTM analysis to define the average levels of core histone PTMs in bulk chromatin. For DNA purification, the sample was mixed 1:1 with 2× de-cross-linking buffer (20 mM Tris (pH 7.6), 600 mM NaCl, 2% SDS) and incubated at 65 °C overnight. The next day, proteinase K was added and the mixture was incubated at 37 °C for 2 h. DNA was purified using the QIAquick PCR purification kit (Qiagen) and eluted in RNase/DNase-free water. RNase A was added and the mixture was incubated at 37 °C for 1 h. DNA was resolved on an agarose gel and visualized with ethidium bromide. Approximately 0.2 mg chromatin (as measured by DNA content) was used for each ChIP reaction with the following antibodies: anti-H3K4me1 (Abcam, ab8895), anti-H3K4me3 (Millipore, 17-614), anti-H3 (Active motif, 39163), anti-H4 (Abcam, ab31830). For H3K4me3 ChIP reactions, 0.6 mg chromatin was used. To boost the identification of H3K4 methylation-state-specific protein interactors, H3 and H4 ChIPs were performed using chromatin inputs partially depleted in H3K4me1- and H3K4me3-modified nucleosomes and co-bound protein factors. Specifically, H3K4me1 and H3K4me3 ChIPs were performed first, then the chromatin inputs used for the H3K4me1 and H3K4me3 ChIPs were combined and subsequently used for H3 and H4 ChIPs. This aimed to shift the composition of the bulk chromatin-associated proteome measured in H3 and H4 control ChIPs towards regions devoid of H3K4me1 and H3K4me3. The antibody–chromatin mixture was incubated overnight on a rotation wheel (25 rpm) at 4 °C. Antibodies were captured using a 1:1 mixture of protein A and protein G Dynabeads (Thermo Fisher Scientific) for 2 h at 4 °C while rotating on a rotation wheel (25 rpm); 40 µl of bead mixture was used per ChIP sample. Beads were washed three times with ice-cold ChIP buffer and twice with ice-cold ChIP buffer supplemented with NaCl to a final concentration of 500 mM. Antibodies and co-bound chromatin were eluted by boiling the beads in 30 µl of Laemmli sample buffer containing 1% SDS and supplemented with 300 mM NaCl for 10 min at 95 °C. The eluate was transferred to a fresh tube and incubated in a thermomixer at 65 °C and 500 rpm for 12 h. For the histone PTM proteomic analysis, eluted proteins as well as the input samples (see above) were resolved on a 4–20% polyacrylamide gel (Novex WedgeWell Tris-Glycin-Minigel, Invitrogen), histone bands were excised, in-gel derivatized, digested with trypsin and processed for LC–MS analysis as described below. For the identification and quantification of co-purified chromatin proteins, a 10 µl aliquot of the eluted proteins in Laemmli sample buffer was processed for trypsin digestion using a FASP protocol and analysed using LC–MS as described below.

#### Native chromatin immunoprecipitations for MS analysis

The HeLa Kyoto BAC cell line expressing the C-terminal LAP-tagged INO80 subunit ACTR5^48^ was cultured as described above. Cells were collected by trypsinization and were washed three times with ice-cold PBS. Nuclei were isolated using a Dounce homogenizer under hypotonic conditions in the presence of 0.1% NP-40 as described previously^25^. Nuclei were resuspended in ice-cold MNase digestion buffer (10 mM Tris (pH 7.6), 15 mM NaCl, 60 mM KCl, 0.1% NP-40) supplemented with protease inhibitor cocktail (Roche) and 10 mM sodium butyrate, and MNase was added at a proportion of 150 U per approximately 20 × 10^6^ nuclei. The nucleus suspension was transferred to a thermomixer and, after 2 min incubation at 37 °C and 400 rpm, CaCl_2_ was added to a final concentration of 1.5 mM and the mixture was incubated at 37 °C for another 6 min. The MNase digestion was stopped by the addition of EDTA to a final concentration of 10 mM. The mixture was then diluted 1:1 with ice-cold 2× SNAP buffer (30 mM HEPES (pH 7.8), 300 mM NaCl, 0.1% NP-40, 20% glycerol, 0.4 mM EDTA) supplemented with protease inhibitor cocktail (Roche) and 10 mM sodium butyrate. The samples were rotated on a rotation wheel for 45 min at 4 °C and further incubated in a thermomixer at 4 °C and 1,000 rpm for another 15 min. Nuclear debris was pelleted by centrifugation (16,000*g*, 10 min, 4 °C). The resulting supernatants were transferred to fresh 1.5 ml low-protein-binding Eppendorf tubes and used for the purification of nucleosomes bound to the INO80 complex as described below. To determine the efficiency of the MNase digestion, the pellets containing the insoluble chromatin fraction were resuspended in 1× supernatant volume of SNAP buffer, supplemented with proteinase K, and incubated at 37 °C overnight. In parallel, 25 µl aliquots of the supernatants were transferred to fresh tubes, supplemented with proteinase K and incubated at 37 °C overnight. After proteins were digested with proteinase K, DNA was extracted using the QIAquick PCR purification kit (Qiagen) and eluted in RNase/DNase-free water. RNase A was added, and the mixtures were incubated at 37 °C for 1 h. The DNA was then resolved on an agarose gel and visualized with ethidium bromide. For each sample, another 25 µl aliquot of the supernatant was transferred to a fresh tube and subsequently used as the input sample to define average histone modification levels on bulk chromatin. For the purification of nucleosomes bound to the INO80 complex, 25 µl of GFP-Trap Agarose beads (ChromoTek) were added to MNase-digested supernatants and the mixture was incubated on a rotation wheel (25 rpm) overnight at 4 °C. The beads were pelleted by centrifugation (250*g*, 3 min, 4 °C), followed by two washes with ice-cold SNAP buffer and one wash with SNAP buffer supplemented with NaCl to the final concentration of 200 mM. The supernatant was completely discarded and the beads were resuspended in 40 µl of SNAP buffer supplemented with 1 µg of 3C protease (Sigma-Aldrich). The mixture was then incubated for 8 h at 4 °C. The beads were pelleted by centrifugation, and the supernatant was transferred to a fresh tube, mixed with Laemmli sample buffer and boiled at 95 °C for 5 min. To identify histone PTMs of INO80-bound nucleosomes the immunopurified proteins and input samples were resolved on a 4–20% polyacrylamide gel (Novex WedgeWell Tris-Glycin-Minigel, Invitrogen), histone bands were excised, in-gel derivatized, digested with trypsin and analysed using LC–MS as described below.

#### CRISPR–Cas9-mediated endogenous protein tagging

The core INO80 complex subunit INO80B was endogenously tagged at its C-terminus with a V5 epitope in the MCF-7 cell line using the tagging strategy described previously^54^. Specifically, 1 day before transfection, MCF-7 cells were seeded onto 24-well plates at approximately 1.0 × 10^5^ cells per well in 500 µl of low-glucose DMEM medium supplemented with 10% FBS, 1 mM glutamine and 100 μg ml^−1^ penicillin–streptomycin. On the day of transfection, 25 µl of Opti-MEM medium was added to a 1.5 ml sterile Eppendorf tube, followed by the addition of 1,250 ng of TrueCut Cas9 Protein v2 nuclease (Invitrogen) and 240 ng of two-piece gRNA (crRNA:tracrRNA duplex) generated by annealing crRNA (IDT) and tracrRNA (IDT) according to the manufacturer’s instructions. After mixing briefly by vortexing, 1 µl Cas9 Plus reagent was added to the solution containing Cas9 protein and gRNA. The mixture was incubated at 25 °C for 5 min to allow the formation of Cas9 ribonucleoprotein particles (RNPs). For co-delivery of homology donor DNA, 800 ng of single-stranded DNA oligonucleotide (IDT) was added to the Cas9 RNPs at this point. Meanwhile, 25 µl Opti-MEM medium was added to a separate sterile Eppendorf tube, followed by the addition of 1.5 µl of Lipofectamine CRISPRMAX. After briefly vortexing, the Lipofectamine CRISPRMAX solution was incubated at 25 °C for approximately 5 min. After incubation, the Cas9 RNPs were then added to the Lipofectamine CRISPRMAX solution. The mixture was incubated at 25 °C for 10–15 min to form Cas9 RNPs and Lipofectamine CRISPRMAX complexes and then added to the cells. At 48 h after transfection, the cells were collected by trypsination and seeded in 96-well plates at 1 cell per well. After reaching 60–80% confluency, the cells were trypsinized and split 1:1 into two 96-well plates where the first plate was used for immunofluorescence screening with monoclonal mouse anti-V5 primary antibodies (eBioscience, TCM5 14-6796-82, 1:250) and Alexa-Fluor-488-coupled anti-mouse IgGs as secondary antibodies (Jackson ImmunoResearch Laboratories, 715-545-150, 1:333), and the second plate was used for the subsequent expansion and further testing of V5-positive clones. The immunofluorescence screen for V5-positive clones was performed as previously described^54^.

#### Co-IP

Approximately 1.0 × 10^7^ MCF-7 WT or INO80B-V5 cells were used for nuclear extract preparations as described previously^25^. The nuclear extract was diluted with IP buffer (20 mM HEPES (pH 7.9), 50 mM NaCl, 0.2 mM EDTA, 5% glycerol, 0.1% NP-40, 1 mM DTT and protease inhibitor cocktail (Roche)) to a final protein concentration of around 1 µg µl^−1^ and a NaCl concentration of 160 mM and subsequently cleared by centrifugation at 20,000*g* for 10 min at 4 °C. Then, 1 ml of cleared nuclear extract was mixed with 5 μl of anti-V5 antibodies (Abcam, ab15828) and incubated on a rotating wheel over night at 4 °C. The next day, 20 µl of a 1:1 mixture of protein A and protein G Dynabeads (Invitrogen) were added to the sample followed by 1 h incubation on a rotation wheel at 4 °C. Magnetic beads were washed three times with the IP buffer containing 150 mM NaCl. Co-immunoprecipitated proteins were eluted from the beads by boiling in 20 µl of Laemmli sample buffer for 5 min at 95 °C. Eluted proteins were subsequently used for immunoblotting and LC–MS experiments (IP–MS). For LC–MS analysis, proteins were digested with trypsin using a FASP protocol as described below.

#### Protein detection by immunoblotting

Proteins were separated by SDS–PAGE and blotted onto nitrocellulose membranes (0.45 µm, Thermo Fisher Scientific) using a Bio-Rad PROTEAN mini-gel and blotting system. Antibodies were diluted in TBST + 5% milk (25 mM Tris (pH 7.5), 137 mM NaCl, 2.7 mM KCl, 0.2% Tween-20, 5% non-fat dry milk). The following primary antibodies were used for immunoblots: anti-V5 tag (eBioscience, TCM5 14-6796-82, 1:1,000), anti-INO80 (Abcam, ab118787, 1:2,000), anti-INO80B (Santa Cruz (E-3), sc-390009, 1:1,000), anti-ACTR5 (GeneTex, GTX80453, 1:1,000), anti-TBRG1 (Santa Cruz (D-9), sc-515620, 1:1,000), anti-H3K4me3 (Millipore, 17-614, 1:2,000), anti-H4 (Abcam, ab31830, 1 µg ml^−1^), anti-H4ac (pan-acetyl) (Active Motif, 39967, 1:1,000), anti-CBX4 (Cell Signaling Technology, E6L7X 30559, 1:1,000), anti-CBX8 (Santa Cruz (C-3), sc-374332, 1:1,000), anti-H2B (Abcam, ab1790, 1:1,000), anti-H2A.Z (Abcam, ab4174, 1:1,000). Immunoblot images were acquired by CCD camera using the Bio-Rad ChemiDoc Touch Imaging System running Image Lab Touch Software (v.2.3.0.07).

### MS methods

#### Sample preparation for MS

##### On-bead digestion and peptide purification for SNAP samples

The beads were resuspended in 50 μl of elution buffer (2 M urea, 100 mM Tris (pH 7.5), 10 mM DTT) and incubated on a shaker (1,000 rpm) at 25 °C for 20 min. Iodoacetamide (Sigma-Aldrich, I1149) was added to a final concentration of 50 mM and the sample was incubated on a shaker (1,000 rpm) at 25 °C in the dark for 10 min. After digestion with 0.3 μg trypsin (Promega V5113) for 2 h on a thermo shaker (1,000 rpm) at 25 °C, the supernatant was transferred to a new tube and was further digested with 0.1 μg trypsin overnight at 25 °C. The digestion was stopped by adding 5.5 μl of 10% trifluoroacetic acid. Eluted peptides were purified on C18 stage-tips (Glygen 10-200 μl TopTips) according to the manufacturer’s instructions and dried using a SpeedVac.

##### FASP of label-free proteomics samples

Filter-aided sample preparation was performed as described previously^52^. In brief, 10–20 µl aliquots of protein mixtures in 1% SDS Laemmli sample buffer were diluted with 200 µl of 100 mM triethylammonium bicarbonate buffer (TEAB; pH 8.5). For protein reduction, 1 µl of 1 M DTT was added to each sample and the samples were incubated at 60 °C for 30 min. After cooling the samples to room temperature, 300 µl of freshly prepared UA buffer (8 M urea in 100 mM TEAB (pH 8.5)) was added to each sample. Proteins were alkylated by the addition of 10 µl of 300 mM iodacetamide solution and subsequent incubation for 30 min at room temperature in the dark. The samples were then concentrated to dryness in a 30 kDa cut-off centrifugal spin filter unit (Millipore), and washed three times with 200 µl UA buffer and twice with 200 µl of 50 mM TEAB (pH 8.5). Then, 40 µl of a 50 ng µl^−1^ trypsin solution in 50 mM TEAB (pH 8.5) was added to each sample and protein digestion was performed overnight at 37 °C. Peptides were centrifuged through the filter, and the collected flow through was acidified by the addition of trifluoroacetic acid to a final concentration of 0.5% (v/v). About 300 ng of the tryptic peptide mixtures was then used for LC–MS analysis as described below.

##### Histone sample preparation for proteomics analysis

Histone proteins were prepared for LC–MS analysis using a hybrid chemical derivatization protocol adopted for in-gel sample preparation. In brief, proteins were resolved on 4–20% polyacrylamide gels (Novex WedgeWell Tris-Glycin-Minigel, Invitrogen) followed by Coomassie staining. Histone protein bands were excised from the gel and destained in a destaining buffer (100 mM triethylammonium bicarbonate in 50% acetonitrile). After destaining, the gel pieces were dehydrated with 200 μl of 100% acetonitrile for 10 min at room temperature after which acetonitrile was discarded. Propionylation solution was prepared by mixing 50 mM TEAB (pH 8.5) and freshly prepared 1% (v/v) propionic anhydride solution in water at a 100:1 ratio. Immediately after preparation, 100 µl of propionylation solution was added to the dehydrated gel pieces followed by 10 min incubation at room temperature. The propionylation reaction was quenched by the addition of 10 μl of 80 mM hydroxylamine and subsequent incubation for 20 min at room temperature. The propionylation solution was discarded and gel pieces were dehydrated with 200 μl of 100% acetonitrile for 10 min at room temperature. After this, the acetonitrile solution was discarded and 20 μl of 50 ng µl^−1^ trypsin solution in 100 mM TEAB (pH 8.5) was added. Trypsin digestion was performed overnight at 37 °C. The next day, 50 μl of 100 mM TEAB (pH 8.5) solution was added to each sample followed by 30 min incubation in a thermo shaker (37 °C, 1,500 rpm). A 1% (v/v) solution of phenyl isocyanate in acetonitrile was freshly prepared and 15 μl added to each sample and incubated for 60 min at 37 °C. The samples were acidified by the addition of 24 μl 1% trifluoroacetic acid. Peptides were desalted with C18 spin columns (Thermo Fisher Scientific) according to the manufacturer’s instructions, dried in a speed-vac, resuspended in 50 μl 0.1% trifluoroacetic acid and subsequently used for LC–MS analysis.

#### LC–MS-based proteomics measurements

##### MS analysis of SNAP samples

SNAP samples were processed and analysed by LC–MS on a Q-Exactive mass spectrometer (Thermo Fisher Scientific) as described previously^55^. In brief, the samples were loaded at 8 μl min^−1^ onto a trap column (Thermo Fisher Scientific, Acclaim PepMap 100; 100 μm internal diameter, 2 cm length, C18 reversed-phase material, 5 μm diameter beads and 100 Å pore size) in 2% acetonitrile and 0.1% trifluoroacetic acid. Each of the samples was loaded twice, providing two technical replicates. Peptides were eluted on line to an analytical column (Thermo Fisher Scientific, Acclaim PepMap RSLC; 75 μm internal diameter, 25 cm length, C18 reversed-phase material, 2 μm diameter beads and 100 Å pore size) and separated using a flow rate of 250 nl min^−1^ and the following gradient conditions: initial 5 min with 4% buffer B; a 90 min gradient of 4–25% B; a 30 min gradient of 25–45% B; a 1 min gradient 45–90% B; and finally 15 min isocratic at 100% B before returning to the starting conditions for a 15 min equilibration (buffer A: 2% acetonitrile and 0.1% formic acid in water; B: 80% acetonitrile and 0.1% formic acid). The Q-Exactive instrument acquired full-scan survey spectra (*m*/*z* 300–1,650) at 70,000 resolution. An automatic gain control target value of 3 × 10^6^ and a maximum injection time of 20 ms were used. The top 10 most abundant multiply charged ions were selected in a data-dependent manner, fragmented by higher-energy collision-induced dissociation, and data were collected over the range 200–2,000 *m*/*z* at 17,500 resolution. An automatic gain control target value of 1 × 10^5^ with a maximum injection time of 120 ms was used. A dynamic exclusion time of 30 s was enabled.

##### MS analysis of label-free proteomics samples

LC–MS/MS analysis of label-free nucleosome pull-downs and ChIP–MS proteomics samples was performed on the Q-Exactive HF mass spectrometer (Thermo Fisher Scientific) coupled in-line to a nanoEasy LC (Thermo Fisher Scientific). The samples were loaded in solvent A (0.1% formic acid) on a two-column set-up consisting of a 3.5 cm, 100 µm inner diameter pre-column packed with Reprosil-Pur 120 C18-AQ (5 µm; Dr. Maisch) and an 18 cm, 75 µm inner diameter analytical column packed with Reprosil-Pur 120 C18-AQ (3 µm; Dr. Maisch). A gradient of solvent B (95% acetonitrile, 0.1% formic acid) was applied at a flow rate of 250 nl min^−1^ as follows: 3% to 25% B in 90 min; 25% to 45% B in 30 min; 45% to 100% B in 3 min; and 100% B in 8 min. MS was obtained at a resolution of 120,000 and MS/MS as top 15 at a resolution of 15,000 and with a dynamic exclusion of 30 s. The maximum injection time was set to 100 ms for both MS and MS/MS and only peptides of charge state 2, 3 and 4 were selected for MS/MS.

LC–MS/MS analysis of INO80-V5 IP–MS samples was performed on the Q-Exactive HF mass spectrometer (Thermo Fisher Scientific) coupled to a nano-RSLC (Ultimate 3000, Dionex). In brief, the samples were automatically loaded onto a nano trap column (300 µm inner diameter × 5 mm, packed with Acclaim PepMap100 C18, 5 µm, 100 Å; LC Packings) before separation by reversed-phase chromatography (HSS-T3 M-class column, 25 cm, Waters) in a 95 min nonlinear gradient from 3 to 40% acetonitrile in 0.1% formic acid at a flow rate of 250 nl min^−1^. Eluted peptides were analysed using the Q-Exactive HF mass spectrometer equipped with a nano-flex ionization source. Full scan MS spectra (*m*/*z* 300–1,500) and MS/MS fragment spectra were acquired in the Orbitrap with a resolution of 60,000 or 15,000, respectively, with maximum injection times of 50 ms each. Up to ten most intense ions were selected for higher-energy collisional dissociation fragmentation depending on signal intensity. Dynamic exclusion was set for 30 s.

##### MS analysis of histone samples

For LC–MS analysis of modified histone proteins, the acidified histone peptide digests were analysed on the Q-Exactive HF mass spectrometer (Thermo Fisher Scientific) coupled in-line to a nanoEasy LC (Thermo Fisher Scientific). In brief, the samples were automatically loaded onto an in-house packed 2 cm 100 µm inner diameter C18 pre-column with buffer A (0.1% formic acid) and then eluted and separated on an in-house packed Reprosil-Pur 120 C18-AQ (3 µm; Dr. Maisch) analytical column (20 cm × 75 µm inner diameter) using a 35 min linear gradient from 0% to 40% buffer B (90% acetonitrile, 0.1% formic acid). Full scan MS spectra (*m*/*z* 300–1,000) and MS/MS fragment spectra were acquired in the Orbitrap with a resolution of 120,000 or 15,000, respectively, with maximum injection times of 50 ms each. Up to the 20 most intense ions were selected for higher-energy collisional dissociation fragmentation depending on signal intensity. Dynamic exclusion was disabled.

#### MS RAW data search and quantification

##### Analysis of SNAP MS data

Protein abundances were quantified from the Q-Exactive raw data files using MaxQuant (v.1.5.2.8)^56^ against the UniProt UP000005640 canonical proteome (downloaded in September 2016) using 2-plex labelling (Arg0/Lys0 and Arg10/Lys8). The search was performed allowing for fixed carbamidomethyl modification of cysteine residues and variable oxidation of methionine residues and acetylation of amino termini. The minimum peptide length was set to 7. All raw files resulting from the forward and reverse pull-downs, including technical replicates for each nucleosome tested, were processed together using the ‘match between runs’ feature. H/L ratios were computed in advanced ratio computation mode, with the minimal ratio and peptide count set to 1. The corresponding mqpar.xml file is deposited along with the proteomics data. Initial trial experiments with mono-, di- and tetra-nucleosomes (Supplementary Information) were quantified separately by MaxQuant v.1.5.1.0 against the December 2015 version of UniProt proteome with more stringent settings requiring at least two peptides for ratio estimation.

##### Analysis of label-free MS data

Protein identification and quantification was performed using Proteome Discoverer v.2.5 (Thermo Fisher Scientific). Data were searched against the human Swiss-Prot database using Mascot^57^ as the search engine, with a precursor mass tolerance of 5 ppm and a fragment mass tolerance of 0.05 Da. Two missed cleavages were allowed for trypsin and carbamidomethylation of cysteine was set as a static modification, while oxidation of methionine was set as dynamic. Label-free quantification was achieved as match between runs by using the Minora Feature Detector, the Feature Mapper and the Precursor Ions Quantifier. The maximum retention time shift for chromatographic alignment was set to 2 min and the retention time tolerance for mapping features was set to 1 min. Peptide quantification was performed as the peak area normalized to the total peptide amount and protein quantification as the average of the top three unique peptides.

##### Analysis of histone MS data

For the identification and quantification of histone PTMs in ChIP–MS samples and the quality control of recombinantly produced modified histone proteins, MS raw data files were manually analysed using Skyline (v.20.1.0.31)^58^. In brief, a list of unmodified as well as differentially modified histone H3 and H4 peptides was manually compiled and used to evaluate the modification status of histones in each sample. All lysine residues not bearing acetylation or methylation were considered to be propionylated and all peptide N termini were considered to be modified with phenyl isocyanate. MS1 filtering was set to include 3 isotope peaks and the MS1 resolving power was set at 120,000. MS2 resolving power was set at 15,000. For each modified histone peptide, the relative abundance was estimated by dividing its peak area by the sum of the areas corresponding to all of the observed forms of that peptide (that is, all peptides sharing the same amino acid sequence). The relative abundance of histone variant H2A.Z was estimated by dividing the sum of peak areas of unique H2A.Z peptides (that is, only present in H2A.Z but not in any other H2A variants) by the sum of peak areas of all unique peptides corresponding to histones H2A, H2B and H2A.Z.

### Data postprocessing and bioinformatic analyses

#### Data postprocessing

##### Postprocessing of SNAP MS data

MaxQuant proteinGroups entries marked as ‘potential contaminant’, ‘reverse’ or ‘only identified by site’ were removed from the datasets analysed. The SILAC H/L ratios for each of the remaining entries were converted to a log_2_ scale. In initial trial experiments (Supplementary Information), the median and first and third quartiles log_2_[H/L ratio] values were estimated in all experiments individually, treating forward and reverse experiments separately. Proteins were assumed to be significantly enriched if they fell 1.5× the interquartile range away from first and third quartiles for both forward and reverse experiments, matching the box plots. The data for the main set of experiments were additionally annotated with up to date (as of 30 July 2019) metadata that were downloaded from the mygene.info API service^59^ based on the IDs in the ‘Majority Protein ID’ column. Protein identifiers were assigned readable counterparts on the basis of the associated gene names. Duplicate entries were enumerated in parentheses (for example, *SMARCA* (1) and *SMARCA* (2)), assigning lower numbers to entries with a higher MaxQuant score. Common prefixes of the gene names were collapsed (for example, *SMAD[2,3,9]*) for brevity. The principal direction of the data spread (that is, the direction of enrichment) in each of the pull-downs was estimated by determining the first principal component of the data in the top-left and bottom-right quadrants of the forward and reverse log_2_[H/L ratio] plot. The estimate was adjusted by re-evaluating the principal direction after removing outlier points ±2 s.d. away from the median in the second principal direction. Protein-specific variation in the second principal direction across pull-down experiments was adjusted to zero to correct systemic heavy and light cell population batch effects resulting from different abundances of proteins in the nuclear extracts from the H/L cell populations or different labelling efficiencies of proteins with the heavy-labelled amino acids. In cases in which either the forward or the reverse H/L ratio was measured for the protein (9.13% of ratio pairs), but not both, the missing ratio was imputed by projecting the measured ratio to the estimated principal enrichment line. In six cases (0.01%) in which the estimated H/L ratio was infinite as protein intensity could have been measured in the modified nucleosome, but not in the unmodified nucleosome, the ratio was imputed to the maximum ratio identified in the particular SNAP experiment. All other missing H/L ratios were imputed to zero (24.27%). Five proteins of which the forward and reverse H/L ratios were equal to zero in all of the experiments were removed. The resulting data for each of the pull-down experiments were then further rotated so the estimated principal direction of variation lays exactly on the ideal 45° diagonal, so the reverse ratio on average equals the negative of the forward one. For visualizations and computational analyses, the sign of the reverse experiment was flipped to be on the same scale as the forward one.

##### Postprocessing of cross-linked H3K4me1 and H3K4me3 ChIP–MS data

Protein abundances obtained from H3K4me1 and H3K4me3 cross-linking-ChIP–MS experiments were converted to log_2_ scale, treating zero abundances as missing data. The data were normalized to ten histone proteins observed in the data: *H2AC20*, *H2AC21*, *H2AW*, *H2AZ2*, *H2BC4*, *H2BU1*, *H3-2*, *H4C1*, *MACROH2A1* and *MACROH2A2*. Specifically, we calculated the average log_2_-transformed abundance for the histone proteins in each of the experiments, and calculated the residuals (that is, log_2_-transformed abundances minus the average (*M* value)) for the histone proteins. The data were normalized by subtracting the median of these residuals for each of the samples, so that the median *M* value of the normalized data for the histone proteins remains approximately zero across experiments. The normalized data were then further filtered to include only proteins that were detected in at least two replicates of at least one experiment.

We used limma^60^ to estimate the log_2_[FC] values between H3K4me3 and controls (H3 and H4), H3K4me1 and controls, and H3K4me3 and H3K4me1. Specifically, we used a zero-intercept means model encoding one parameter for each experiment (H3, H4, H3K4me1, H3K4me3), and analysed the contrasts between protein abundance in H3K4me1/3 experiments and the average abundance of H3 and H4 (for example, (H3 + H4)/2), as well as a contrast between H3K4me3 and H3K4me1. The analysis was run using the default parameters of limma (v.3.50.1), with the addition of ‘robust=True’ in the ‘eBayes’ step, hypothesis testing was performed using the default settings, assuming zero log_2_[FC] under the null hypothesis. *P* values were corrected using the Benjamini–Hochberg procedure, and significance was assumed at an FDR of 0.05.

In some cases, the contrasts could not be estimated due to missing data. This frequently happened when proteins were detected in one of the experiments, but not in controls (or vice versa). In these cases, we imputed such log_2_[FC] estimates with infinities (positive and negative). Moreover, whenever it was possible to estimate the H3 or H4 controls, but not both, we imputed the log_2_[FC] estimates using one of such controls only. The imputed estimates are clearly flagged in the data and figures. Estimates based only on single data points (that is, an observed abundance in one of the three replicates only) are flagged as well.

To be able to link the ChIP–MS data with MARCS feature effect estimates, we mapped the ChIP–MS proteins to their MARCS counterparts through their accession numbers and gene names. The cases in which one ChIP–MS protein mapped to multiple proteins in the MARCS dataset were resolved by assigning the feature effect estimate with the lowest *P*-value estimate across all of the matched identifiers.

To obtain association statistics, we performed a Mann–Whitney *U*-test, comparing the imputed ChIP–MS log_2_[FC] estimates of proteins strongly recruited to or excluded by a MARCS feature to the imputed log_2_[FC] estimates of other proteins detected in both MARCS and ChIP–MS data. Only the groups with at least five proteins were tested. For visualization purposes, we computed the mean log_2_[FC] estimates in each of the groups, and their respective differences. For this purpose, we assumed the infinities to be equal to the maximum finite log_2_[FC] plus a small number.

##### Postprocessing of variable-linker nucleosome pull-down data

Label-free MS quantification datasets for the short linker nucleosome, long linker SV40 promoter nucleosome and long linker SV40 enhancer nucleosome affinity-purification experiments were analysed independently. The protein abundances were converted to a log_2_ scale, treating zero intensities as missing values. The data were normalized using the abundances of HIST1H4A and HIST2H2BF histones (short linkers) or H4C1 and H2BC12 histones (long linkers) as described in the H3K4me1/3 cross-linking-ChIP–MS methods.

For each set of experiments, we used a zero-intercept means model in limma and hand-crafted contrasts to measure two types of effects on protein binding to dinucleosomes: (1) modification-specific effects, that is, the log_2_-transformed FC in protein abundance between modified nucleosome and unmodified nucleosome, given a specific linker of certain length, for example, log_2_[H3K27me3 with 50 bp linker] versus log_2_[unmodified with 50 bp linker], as well as (2) linker-specific effects, that is, the log_2_-transformed FC in protein abundance between two different linkers, given a certain nucleosome modification, for example, log_2_[H3K27me3 with 55 bp linker] versus log_2_[H3K27me3 with 50 bp linker]. Owing to the large number of missing values, the second replicate of the H3K27me3 experiment with 35 bp linker was excluded from the analysis. Only proteins that had at least two values in at least one condition were analysed.

The analysis was run using the default parameters of limma (v.3.50.1), using the ‘robust=True’ parameter in the ‘eBayes’ step. *P* values were corrected using the Benjamini–Hochberg procedure, assuming significance at an FDR of 0.05. In addition to this, significant estimates were considered to be ‘strong’ if the absolute log_2_[FC] was greater than 1.

As in the H3K4me1/3 cross-linking-ChIP–MS experiment, we imputed contrasts that could not be estimated from the data using the following heuristics: proteins detected in one of the conditions, but not the other, received either infinite enrichments or infinite depletions. Such imputed estimates were flagged in the data, together with estimates based on single data points.

To aid the data visualization, we divided the proteins into three groups on the basis of the effects of the modifications and linkers on dinucleosome binding in the different analyses: (1) modification-responsive proteins, that is, proteins that have a significant and strong response to a modification signature in at least one of the linkers visualized; (2) linker-responsive proteins, that is, proteins with a significant and strong response to the linker in either modified or unmodified nucleosomes; and (3) proteins that respond to both, that is, satisfy conditions (1) and (2) simultaneously.

##### Postprocessing of endogenous INO80B-V5 IP–MS data

For analysis of INO80B-V5 IP-MS data, only proteins identified based on three or more unique peptides were considered. The quantified MS1 protein abundances were normalized to the IGHG1 abundance. Differential enrichment analysis was performed using a two-tailed *t*-test. *P* values were adjusted for multiple comparisons using the Benjamini–Hochberg method. The protein stoichiometry was determined using MS1-based label-free quantification^61^. Specifically, protein abundances were calculated as the mean of MS1 intensities of all unique peptides identified for the protein. To assess the stoichiometry of INO80 complex subunits, the abundance of each subunit (mean of unique MS1 peptide intensities) was divided by the abundance of INO80B (mean of unique INO80B MS1 peptide intensities) used as a bait in co-IP complex purification experiments.

#### Decoupling of the effects of individual modification features (SNAP dataset)

Pairs of nucleosomes differing by a single modification only were identified by arranging the nucleosomes into a directed graph of which the edges track the difference by one modification, including self-informative nucleosomes that contain only one chromatin feature (for example, H3K4me3). H3K9acK14ac, full acetylation on histone H3 (H3K9acK14acK18acK23acK27ac), H4K5acK12ac and fully acetylated H4 (H4K5acK8acK12acK16ac) were treated as single modification. Only chromatin features that have two or more informative nucleosome pairs, and therefore an independent experimental replicate, were analysed. As each pull-down consists of a forward and reverse experiment, this results in at least four experimental measurements, enabling a robust statistical analysis. Moreover, a feature effect estimate was computed only for proteins that have at least one nucleosome pair with no imputed data.

The relationship between nucleosomes was modelled in limma using the following formula: ‘~ 0 + edge + ptm’. Here the ‘edge’ parameter tracks edges in the directional graph and ptm captures the direction of the edge and is set to one at the endpoint that contains the target feature and zero at the other. This expression allows the baseline effect of a nucleosome pair to be captured by the ‘edge’ parameter allowing the ‘ptm’ parameter to measure the change of the effect caused by the modification feature (that is, a PTM, histone H2A.Z or DNA methylation). Self-evident purifications were assigned no edge coefficient. Limma was run with robust empirical Bayes, with weights set to number of unique peptides detected plus one. Significance was assumed at Benjamini–Hochberg-adjusted FDR of 0.01.

Significant responses were additionally labelled as strong if their parameter estimates were greater than or equal to 1. For the proteins that respond strongly to at least one feature, the collective modification response profiles across all features were clustered. The clustering was performed using protoclust^62^ (v.1.6.3) under cosine distance. The dendrogram corresponds to Minimax Hierarchical Linkage. In cases in which no estimate for the effect could be made, for clustering purposes the values were imputed using three nearest neighbours (bnstruct package^63^). The resulting dendrogram was divided into 40 flat clusters that were annotated with their respective prototype protein in Fig. 2e and Supplementary Table 5.

The joint response of protein complexes to chromatin features was analysed using CAMERA^64^. Only complexes with 3–40 members (inclusive) were analysed. Significance was assumed at a Benjamini–Hochberg-adjusted FDR of 0.01. Whenever possible, the enrichment of both the whole protein complex, and the enrichment of only the exclusive subunits of the complex, not including subunits shared with other complexes, was tested. The median effect of chromatin features on protein complexes was estimated from 100,000 random samples from the effect distributions of individual subunits. The median, as well as the empirical 95% confidence interval (CI) is reported.

#### Network inference (SNAP dataset)

We used the network inference algorithms ARACNE, MRNET and CLR implemented in the minet package^36^ to infer the protein–protein interaction networks in an unsupervised manner, using only the 1,915 × 110 matrix of processed log_2_-transformed heavy/light ratios of identified proteins as the input. The algorithms were configured to use Miller-Madow (mi.mm) estimator for MI and the equal width discretization strategy with the bin number set to 10. In addition to the algorithms above, the performance of the MI metric on its own (without subjecting it to network algorithms) was also evaluated (network RAW-MI).

In addition to the MI-based methods above, we have benchmarked the networks defined by the interprotein correlation matrix computed both naively (CORR) or using Ledoit–Wolf shrinkage (CORR-LW)^65^. These networks were built by assuming the adjacency between the nodes to be equal to the corresponding entry in the correlation matrix. Negative values in the correlation matrix were avoided by adding one to each of the entries and dividing the result by two.

The inferred networks were evaluated against the BioGRID database^34^ (release 3.5.174) after training. BioGRID entries were linked with our identifiers through Entrez identifiers downloaded previously through the mygene.info API service^59^. Networks were evaluated by computing their precision (fraction of predicted edges in the network that were also in the BioGRID database) and recall (fraction of edges in BioGRID database that were predicted by the network) at multiple stringency levels. We used the scaled truncated area under precision and recall curve (auPRC) statistic^66^, which combines the multiple precision/recall estimates into a single score as our primary metric. As we did not anticipate a full recovery of BioGRID interactions by our networks and therefore wanted to trade higher precision for lower recall, we did not consider any threshold settings with a recall of greater than 0.2 for the evaluation of the algorithms. Interactions with histone proteins, as well as self-interactions (either homodimers in BioGRID or interactions between two proteins with the same gene name) were excluded from the evaluation.

To produce the inferred networks described in the paper, we noted that the scores of the CLR algorithm can be converted to *P* values by noting that for the CLR scoring function $s(i,j)=\sqrt{\max {(0,{z}_{i})}^{2}+\max {(0,{z}_{j})}^{2}}$ where *z*_*i*_ and *z*_*j*_ are assumed to follow standard normal distribution under the null hypothesis^35,36^, the *P* values under null can be expressed as $P(s(i,j)\ge x > 0)=\frac{1}{4}(2\times \text{erfc}(\frac{x}{\sqrt{2}})+{e}^{-{x}^{2}/2})$. Where erfc is the complementary error function. Adjusting those computed *P* values for multiple hypothesis testing using the Benjamini–Hochberg procedure (that is, converting them to a *q* value) enabled us to pick a set of intuitive thresholds to produce the networks presented in the paper.

Networks at different adjusted *q*-value thresholds were drawn using the Force Atlas 2 algorithm in gephi^67^ and adjusted manually. Only proteins with at least five non-zero values were drawn. Isolated nodes (connected components with size of 1) were not drawn. Network nodes were either coloured by communities (Louvain algorithm^68^ implemented in the Python-Louvain package) or overlaid by the colour-coded chromatin response estimates (see the ‘Decoupling of the effects of individual modification features (SNAP dataset)’ section above). In the network projection plots, the names of protein complexes were annotated manually on the basis of protein complexes that were significantly regulated by the chromatin modification (as reported by the CAMERA procedure), and had empirically estimated median effects of at least 0.3. Expert judgement was used to disambiguate complexes with a high number of shared subunits, as well as to determine which labels to exclude to reduce crowding. Protein complexes that did not form tight clusters in the network were not annotated.

An additional high confidence network was generated for protein interaction predictions by selecting a network threshold at which 70% precision was achieved. BioGRID interactions that were not predicted by the algorithm (false negatives) were added to the network plot. The network was visualized using cytoscape^69^. Network node labels and annotations were added to the network manually. Both high-confidence and standard network interaction predictions are provided in Supplementary Table 7.

#### Curation of protein complex list (SNAP dataset)

A curated protein complex list was seeded with complexes downloaded from the EBI complex portal version 19 July 2019 (ref. ^38^) and the EpiFactors database (obtained on 29 July 2019)^37^. Protein members of the complexes that were not detected in our experiments were filtered out. Only complexes with at least two protein subunits left after filtering were retained, merging protein complexes that became indistinguishable (that is, had the same subunits) after filtering. Protein complex annotations from the databases that were substantially similar (for example, variants of protein complexes defined by redundant adapter proteins) were merged together based on manual review. Missing annotations from the databases were added manually based on the review of the inferred protein network and corresponding literature. In some cases, the entries were also augmented with data from CORUM^70^ and UniProt^71^. Where possible, protein complexes were renamed manually to match the canonical designations. All sources of annotations were recorded and are available in Supplementary Table 8.

#### Integration of MARCS with ChIP–seq data

For joint MARCS and ChIP–seq analysis, the relevant ENCODE^30^ ChIP–seq, DNase-seq and ATAC–seq datasets for the K562 cell line were downloaded together with the chromatin state predictions from ROADMAP^1^. We next divided the hg38 reference genome, excluding blacklisted regions^72^ and chromosome Y, into a set of non-overlapping 1,000-bp-wide bins and marked the bins containing peaks from each of the NGS datasets. We have assumed each of the genomic bins to be independent and identically distributed and therefore modelled the presence or absence of a given peak as a Bernoulli event. For a given pair of NGS datasets, we therefore computed their joint distribution by counting the bins for which both datasets are co-present, co-absent and mutually exclusive (both ways). A pseudocount of 100 was added to avoid zeroes and to smooth the probability estimates. This joint distribution enabled us to compute the MI between two NGS datasets, which is equivalent to the Kullback–Leibler divergence from the joint distribution under independence. To obtain an interpretable statistic that measures the fraction of information about *A* that can be predicted by knowing *B*, the MI was divided by the Shannon entropy (*H*) of one of the two datasets: *U*(*A*,*B*) = MI(*A*,*B*)/*H*(*A*). We frequently refer to this ratio as fraction of entropy of *A* explained by *B* or, simply, the normalized MI. As a convention, we use this to measure the fractional entropy of a protein (for example, PHF8) NGS experiment that the knowledge of a chromatin feature (such as H3K4me3) NGS experiment provides, for example, *U*(PHF8, H3K4me3) = MI(PHF8, H3K4me3)/*H*(PHF8) (Extended Data Fig. 4a).

We next compared these normalized MI estimates for each of the MARCS-identified proteins for which ENCODE ChIP–seq data were available in K562 cells. For each of the MARCS chromatin features, and for each of the ChIP–seq chromatin features, we measured whether the proteins predicted to be strongly recruited or strongly excluded by MARCS feature had significantly higher or lower uncertainty coefficients, when compared to proteins neither strongly recruited nor strongly excluded, or proteins identified in MARCS for which we had no MARCS feature effect estimates at all. For these comparisons, we used a Mann–Whitney *U*-test (two-sided) and Benjamini–Hochberg correction. For the benefit of visualization we also computed the differences between mean log_2_-normalized MI estimates for MARCS-feature-associated proteins and others. In cases in which proteins had multiple ChIP–seq replicates, we used the harmonic average of their normalized MI coefficients for the analysis. We treated replicates of chromatin feature ChIP–seq analyses independently. In cases in which one ChIP–seq protein mapped to multiple MARCS proteins, we used the chromatin feature effect estimates from the proteins with the lowest *P* value.

As an additional similarity metric to the normalized MI statistic described above, we computed the Kendall correlation between the peak heights (as defined by the column 7 signalValue in the ‘narrowPeak’ and ‘broadPeak’ file formats) for genomic bins for which the peaks were co-present. This metric is used in Extended Data Fig. 4e–j.

For verification of the network analysis results in Extended Data Fig. 7f, we divided each pair of proteins for which ChIP–seq data were available into groups based on the confidence of inferred interactions from the MARCS-based network analysis. In the case of multiple mappings to MARCS, the highest-confidence outcome was chosen. For each such pairs, we computed the symmetric variant of normalized MI statistic: *U*_sym_(*A*,*B*) = (2MI(*A*,*B*))/(*H*(*A*) + *H*(*B*)), based on their ChIP–seq datasets. The statistics of replicate experiments were averaged harmonically. A one-sided Mann–Whitney *U*-test was used to test whether the distribution of symmetric normalized MI coefficients is statistically different across the MARCS confidence levels (Bonferroni correction).

### Statistics

The details of quantification and statistical analyses are described in detail in the Methods. Where appropriate, the necessary information is also described in the figure legend.

### Reporting summary

Further information on research design is available in the Nature Portfolio Reporting Summary linked to this article.

## Online content

Any methods, additional references, Nature Portfolio reporting summaries, source data, extended data, supplementary information, acknowledgements, peer review information; details of author contributions and competing interests; and statements of data and code availability are available at 10.1038/s41586-024-07141-5.

### Supplementary information

Supplementary Information Additional information and controls for SILAC and label-free nucleosome affinity purifications, MARCS data processing, quality controls for nucleosome assembly and modified histone proteins, and a list of key resources.

Reporting Summary

Supplementary Fig. 1 Gel raw data and graph source data.

Supplementary Table 1 Post-processed data of 55 SILAC dinucleosome-purification experiments.

Supplementary Table 2 Heat-map visualization of the 55 SILAC dinucleosome-purification experiments.

Supplementary Table 3 Predicted effect of chromatin modification features on protein binding.

Supplementary Table 4 Integrative analysis of ENCODE NGS datasets and NIH Roadmap chromatin states with MARCS feature effect estimates.

Supplementary Table 5 Clustered groups of chromatin feature effect estimates.

Supplementary Table 6 List of H3K4me1- and H3K4me3-associated proteins in IMR-90 cells.

Supplementary Table 7 List of protein–protein interaction predictions and their responses to chromatin modification features.

Supplementary Table 8 List of protein complexes and their predicted chromatin responses.

Supplementary Table 9 Label-free MS quantification results of protein binding responses to dinucleosomes incorporating various linker DNAs.

Supplementary Table 10 Key resources table.

## Data Availability

Gel raw data for the immunoblots shown in Fig. 5e and Extended Data Figs. 2b and 5g,h,j and a graph source data table providing the number of feature effect estimate measurements for the H3ac and H4ac features for each of the protein complexes displayed in the bar graph in Fig. 3d are provided in Supplementary Fig. 1. The MS data have been deposited at the ProteomeXchange Consortium via the PRIDE^73^ partner repository (https://www.ebi.ac.uk/pride/) under the following identifiers: SILAC dinucleosome-purification experiments (PXD018966; the H4K20me2 samples from this experiment were previously deposited with identifier PXD009281 as part of ref. ^55^); H3K4me1 and H3K4me3 ChIP–MS (analysis of histone PTMs; PXD042224); H3K4me1 and H3K4me3 ChIP–MS (analysis of co-purified proteins; PXD042826); label-free dinucleosome-purification experiments with 200 bp SV40 promoter linker (PXD041835); label-free dinucleosome-purification experiments with 200 bp SV40 enhancer linker (PXD041443); label-free dinucleosome-purification experiments with short linkers and heterochromatic PTMs (PXD042368); IP–MS analysis of the human INO80 complex composition and interactome (PXD020712); ChIP–MS analysis of histone PTMs co-purified with the human INO80 complex (PXD042210); analysis of the effect of native chemical ligation on protein binding (PXD042390); MS analysis of ligated and recombinant human histones H3 and H4 (PXD020773); analysis of the stability of nucleosomal modifications during affinity purification in nuclear extract (PXD042823). Moreover, the SILAC nucleosome affinity purification data presented in this publication are available in an interactive format online (https://marcs.helmholtz-munich.de). The following public databases were used for data analyses in this study: BioGRID^34^ (https://thebiogrid.org/); CORUM^70^ (https://mips.helmholtz-muenchen.de/corum/); Complex portal^38^ (https://www.ebi.ac.uk/complexportal/home); ENCODE^30^ (https://www.encodeproject.org/); EpiFactors^37^ (http://epifactors.autosome.ru/); Mygene.info^59^ (https://mygene.info/); UniProt/Swiss-Prot^71^ (https://www.uniprot.org). A detailed list of ENCODE datasets used for the integration of MARCS with ChIP–seq data, including ENCODE accession numbers, is provided in Supplementary Table 4. A list of key resources and reagents used in this study is provided in Supplementary Table 10 and the Supplementary Information.

## Code availability

The source code developed for this study for data processing and analyses (https://github.com/lukauskas/publications-lukauskas-2024-marcs) and for the interactive web interface (https://github.com/lukauskas/marcs) are available at GitHub. Detailed information about software used in this manuscript is provided in the ‘key resources table’ in Supplementary Table 10 and the Supplementary Information.
