# Publisher Correction: Decoding chromatin states by proteomic profiling of nucleosome readers

**DOI:** 10.1038/s41586-024-07392-2

**Published:** 2024-04-09

**Authors:** Saulius Lukauskas, Andrey Tvardovskiy, Nhuong V. Nguyen, Mara Stadler, Peter Faull, Tina Ravnsborg, Bihter Özdemir Aygenli, Scarlett Dornauer, Helen Flynn, Rik G. H. Lindeboom, Teresa K. Barth, Kevin Brockers, Stefanie M. Hauck, Michiel Vermeulen, Ambrosius P. Snijders, Christian L. Müller, Peter A. DiMaggio, Ole N. Jensen, Robert Schneider, Till Bartke

**Affiliations:** 1 Institute of Functional Epigenetics, Helmholtz Zentrum München, Neuherberg, Germany; 2 MRC Laboratory of Medical Sciences (LMS), London, UK; 3 Department of Chemical Engineering, Imperial College London, London, UK; 4 Institute of Clinical Sciences (ICS), Faculty of Medicine, Imperial College London, London, UK; 5 Institute of Computational Biology, Helmholtz Zentrum München, Neuherberg, Germany; 6 Department of Statistics, Ludwig Maximilian University Munich, Munich, Germany; 7 Proteomic Sciences Technology Platform, The Francis Crick Institute, London, UK; 8 VILLUM Center for Bioanalytical Sciences and Department of Biochemistry and Molecular Biology, University of Southern Denmark, Odense, Denmark; 9 Department of Molecular Biology, Faculty of Science, Radboud Institute for Molecular Life Sciences, Oncode Institute, Radboud University Nijmegen, Nijmegen, The Netherlands; 10 The Netherlands Cancer Institute, Amsterdam, The Netherlands; 11 Metabolomics and Proteomics Core, Helmholtz Zentrum München, Munich, Germany; 12 Center for Computational Mathematics, Flatiron Institute, New York, NY USA; 13 Faculty of Biology, Ludwig Maximilian University Munich, Martinsried, Germany; 14 German Center for Diabetes Research (DZD), Neuherberg, Germany; 15 Present Address: Northwestern Proteomics Core Facility, Northwestern University, Chicago, IL USA; 16 Present Address: Clinical Protein Analysis Unit (ClinZfP), Biomedical Center (BMC), Faculty of Medicine, Ludwig Maximilian University Munich, Martinsried, Germany

**Keywords:** Protein-protein interaction networks, Protein databases, Proteome informatics, Histone post-translational modifications, Epigenetics

Correction to: *Nature* 10.1038/s41586-024-07141-5 Published online 6 March 2024

In the version of the article initially published, the first column of data points for RNF2 and EZH2 on the left-hand side of Fig. 1e were mistakenly shifted to the far right. In Fig. 4d, “6” was missing from the *x* axis and in Fig. 5e the left “+” was missing from the “H4ac” row. These errors have now been corrected in the HTML and PDF versions of the article.

