# Supplementary material for: Decoding chromatin states by proteomic profiling of nucleosome readers
Source: Nature. 2024 Mar 6;627(8004):671–9. doi: 10.1038/s41586-024-07141-5 (PMC10954555; doi:10.1038/s41586-024-07141-5)
Supplement: Supplementary file 1 — Additional information and controls for SILAC and label-free nucleosome affinity purifications, MARCS data processing, quality controls for nucleosome assembly and modified histone proteins, and a list of key resources. [file 41586_2024_7141_MOESM1_ESM.pdf]

nature portfolio

https://doi.org/10.1038/s41586-024-07141-5

Supplementary information

Decoding chromatin states by proteomic profiling of nucleosome readers

In the format provided by the authors and unedited

Supplementary Information

Decoding Chromatin States by Proteomic Profiling of Nucleosome Readers

Saulius Lukauskas, Andrey Tvardovskiy, Nhuong V. Nguyen, Mara Stadler, Peter Faull, Tina Ravnsborg, Bihter Özdemir Aygenli, Scarlett Dornauer, Helen Flynn, Rik G. H. Lindeboom, Teresa K. Barth, Kevin Brockers, Stefanie M. Hauck, Michiel Vermeulen, Ambrosius P. Snijders, Christian L. Müller, Peter A. DiMaggio, Ole N. Jensen, Robert Schneider and Till Bartke

Table of Contents

Table of Contents 2
Nucleosome affinity pull-down tests for the optimisation of SNAP experiments 4
Nucleosome affinity pull-down tests examining the effect of native chemical ligation on protein binding to di-nucleosomes 6
Design of MARCS di-nucleosome library 7
SDS-PAGE of ligated and recombinant histone products 8
SDS-PAGE of refolded histone octamers 8
Unmethylated and CpG-methylated biotin-601 di-nucleosome DNA 9
Biotin-601 di-nucleosome DNAs incorporating different linker lengths 10
Native PAGE EMSA gels of reconstituted di-nucleosomes 12
Summary of the data postprocessing algorithm for the SNAP mass spectrometry data 13
Distribution of estimated enrichment axes for each of the SNAP experiments 14
Verification of modification status of ligated histone products by mass spectrometry 15
List of peptides used for MS-based evaluation of the ligated H3 and H4 protein quality. 15
Histone H3 products 16
Unmodified Histone H3 (recombinant, histone product 1a) 16
Unmodified Histone H3 (ligated, histone product 1b) 17
H3K9me2 (histone product 2) 18
H3K9me3 (histone product 3) 19
H3K9me3K27me3 (histone product 4) 20
H3K27me2 (histone product 5) 21
H3K27me3 (histone product 6) 22
H3K4me1K27me3 (histone product 7) 23
H3K4me1K18acK23acK27me3 (histone product 8) 24
H3K4me1K9acK14acK27me3 (histone product 9) 25
H3K4me1 (histone product 10) 26
H3K4me1K27ac (histone product 11) 27
H3K27ac (histone product 12) 28
H3K4me1K9acK14ac (histone product 13) 29
H3K9acK14ac (histone product 14) 30
H3K4me3K9acK14ac (histone product 15) 31
H3K4me3K9acK18ac (histone product 16) 32
H3K4me3K9ac (histone product 17) 33
H3K4me3 (histone product 18) 34
H3K4me3K14ac (histone product 19) 35
H3K4me1-5ac (H3K4me1K9acK14acK18acK23acK27ac, histone product 20) 36
H3K4me3-5ac (H3K4me3K9acK14acK18acK23acK27ac, histone product 21) 37
Histone H4 products 38
Unmodified Histone H4 (recombinant, histone product 22a) 38
Unmodified Histone H4 (ligated, histone product 22b) 38
H4K20me2 (histone product 23) 39
H4K20me3 (histone product 24) 39
H4K16ac (histone product 25) 40

H4K5acK12ac (histone product 26) ... 40
H4K5acK8acK12ac (histone product 27) ... 41
H4K5acK8acK12acK16ac (histone product 28) ... 41
H4K5acK8acK12acK16acK20me2 (histone product 29) ... 42

Evaluation of the stability of nucleosomal modifications during the incubation with HeLa nuclear extract (part 1) ... 43

Evaluation of the stability of nucleosomal modifications during the incubation with HeLa nuclear extract (part 2) ... 45

Key Resources Table ... 47

Supplementary Material Legends ... 61

Supplementary Figure 1. Gel raw data and graph source data ... 61

Supplementary Table 1. Post-processed data of 55 SILAC di-nucleosome purification experiments ... 61

Supplementary Table 2. Heatmap visualisation of the 55 SILAC di-nucleosome purification experiments ... 61

Supplementary Table 3. Predicted effect of chromatin modification features on protein binding ... 61

Supplementary Table 4. Integrative analysis of ENCODE NGS datasets and NIH Roadmap chromatin states with MARCS feature effect estimates ... 62

Supplementary Table 5. Clustered groups of chromatin feature effect estimates ... 62

Supplementary Table 6. List of H3K4me1- and H3K4me3-associated proteins in IMR-90 cells ... 62

Supplementary Table 7. List of protein-protein interaction predictions and their responses to chromatin modification features ... 62

Supplementary Table 8. List of protein complexes and their predicted chromatin responses ... 63

Supplementary Table 9. Label-free MS quantification results of protein binding responses to di-nucleosomes incorporating various linker DNAs ... 63

Supplementary Table 10. Key resources table ... 63

References (Supplementary Information) ... 64

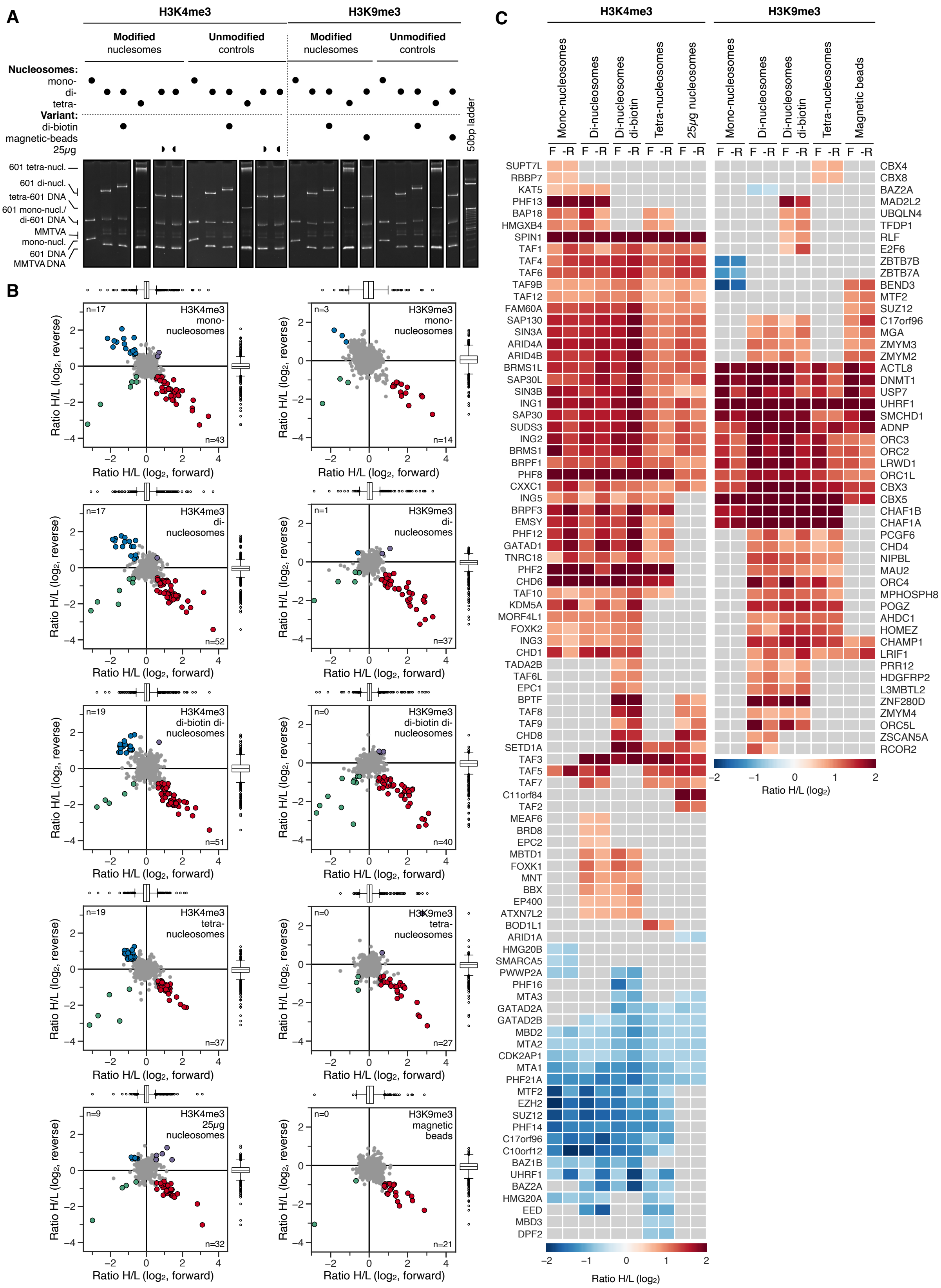

Nucleosome affinity pull-down tests for the optimisation of SNAP experiments

(legend on the next page)

(continued from previous page)

(A) Verification of the assembly of unmodified (control), H3K4me3-, and H3K9me3-modified mono-, di-, and tetra-nucleosomes with one or two biotin handles, as well as the nucleosomes used for magnetic beads-based purification and the 25 μg (2x amount) test SNAP experiments. Equal amounts of the assembled nucleosomes before immobilisation were separated on 5% native PAGE gels and stained with ethidium bromide. The positions of assembled mono-, di-, and tetra- nucleosomes and corresponding unassembled nucleosomal DNAs, and of the MMTVA competitor DNA and MMTVA nucleosomes formed during the assembly reaction are indicated on the left. The rightmost marker lane corresponds to the Invitrogen 50bp DNA ladder. Note that nucleosomes for the 25 μg experiment were prepared in two batches - one was used in the forward, the other in the reverse experiment. The batches are annotated with half-circles. (B) SILAC scatterplots of the results of the trial pull-downs for the different H3K4me3- and H3K9me3-modified mono-, di-, and tetra- nucleosomes as shown in (A). The number of significantly enriched (bottom right quadrant) or depleted (top left quadrant) proteins is highlighted in each experiment. Average ratios of two technical replicates of the forward and reverse pull-down experiments (n=2 biologically independent replicates) are plotted. Significant enrichment was determined from Tukey boxplot statistics of $\log_2$ H/L ratios, marking proteins 1.5 inter-quartile range (i.e. 1.5 x (Q3 - Q1)) away from the first (Q1) and third quartiles (Q3) as significant. The median (Q2) is marked by a line in each boxplot. (C) Significantly regulated proteins in panel (B) visualised in a heatmap format indicating the number of identified proteins and the degree of enrichment or depletion under the different pull-down conditions as described in (A). The reverse H/L ratio was inverted in order for both ratios to be on the same scale. Grey squares mark proteins that were not detected as significantly enriched in particular experiments. The comparison of mono-, di-, and tetra-nucleosomes, biotinylation of one or both ends of the DNA, sepharose or magnetic streptavidin beads, and the use of increased amounts of nucleosomes indicates that the highest number of proteins showing modification-dependent binding is identified in pull-downs with 12.5 μg of di-nucleosomes and streptavidin sepharose beads. Given that di-nucleosomes biotinylated at both ends did not assemble with sufficient quality (see native PAGE gels in (A)), and the concern that double biotinylation might bridge between beads or cause torsion or steric hindrance due to immobilisation at both ends, single-end biotinylated di-nucleosomes were used for all SNAP experiments.

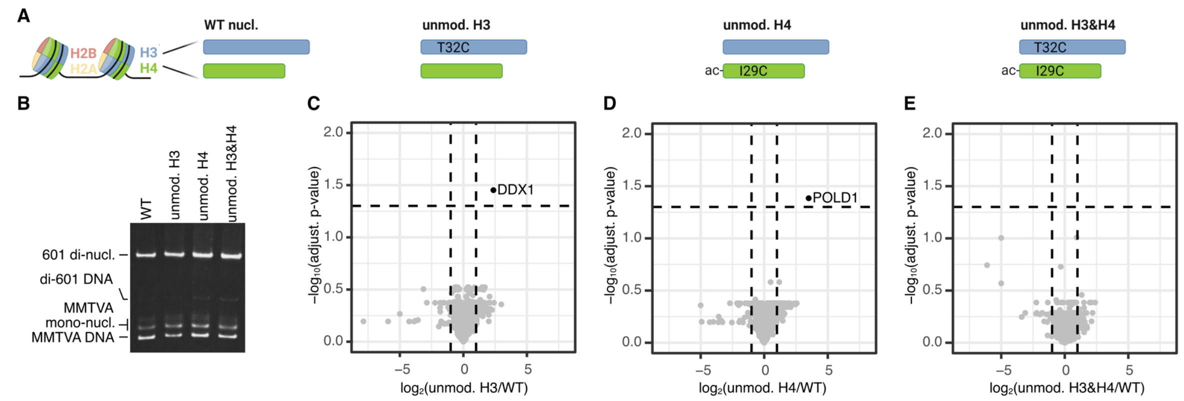

Nucleosome affinity pull-down tests examining the effect of native chemical ligation on protein binding to di-nucleosomes

(A) Schematic representation of a di-nucleosome incorporating unmodified recombinant wild type histones H3 and H4 (WT), ligated unmodified histone H3 and recombinant wild type histone H4 (unmod. H3), recombinant wild type histone H3 and ligated unmodified histone H4 (unmod. H4), as well as ligated unmodified histones H3 and H4 (unmod. H3&H4). Note that ligated histones H3 and H4 contain T32C or I29C single amino acid substitutions, respectively, that are introduced by the native chemical ligation procedure. In addition, ligated histone H4 is N-terminally acetylated to mimic its naturally blocked N-terminus. (B) Verification of the correct assembly of the WT control, unmod. H3, unmod. H4, and unmod. H3&H4 di-nucleosomes used in the pull-down experiments shown in panels (C-E). Equal amounts of the assembled nucleosomes before immobilisation were separated on a 5% native PAGE gel and stained with ethidium bromide. The positions of the assembled di-nucleosomes, unassembled nucleosomal DNAs, and of the MMTVA competitor DNA and MMTVA nucleosomes formed during the assembly reaction are indicated on the left. (C-E) Label-free quantitative MS analysis of pull-down experiments performed with WT, unmod. H3, unmod. H4, and unmod. H3&H4 di-nucleosomes and HeLa S3 nuclear extract. Each label-free nucleosome affinity purification was performed in n=3 independent experimental replicates as described in the methods. The volcano plots show the comparison of nuclear protein binding between the WT di-nucleosomes and di-nucleosomes incorporating either ligated H3 and WT H4 (C), WT H3 and ligated H4 (D), or ligated H3 and ligated H4 (E). A two-tailed Student's t-test was used for statistical analysis and p-values were adjusted for multiple comparisons using the Benjamini/Hochberg approach. The dashed lines indicate FDR = 0.05 and log_2FC (fold change) = 1 or -1 compared to the WT di-nucleosomes. Note that the protein binding profiles between di-nucleosomes containing unmodified ligated histones H3 and H4 and di-nucleosomes containing unmodified recombinant WT histones H3 and H4 are highly similar.

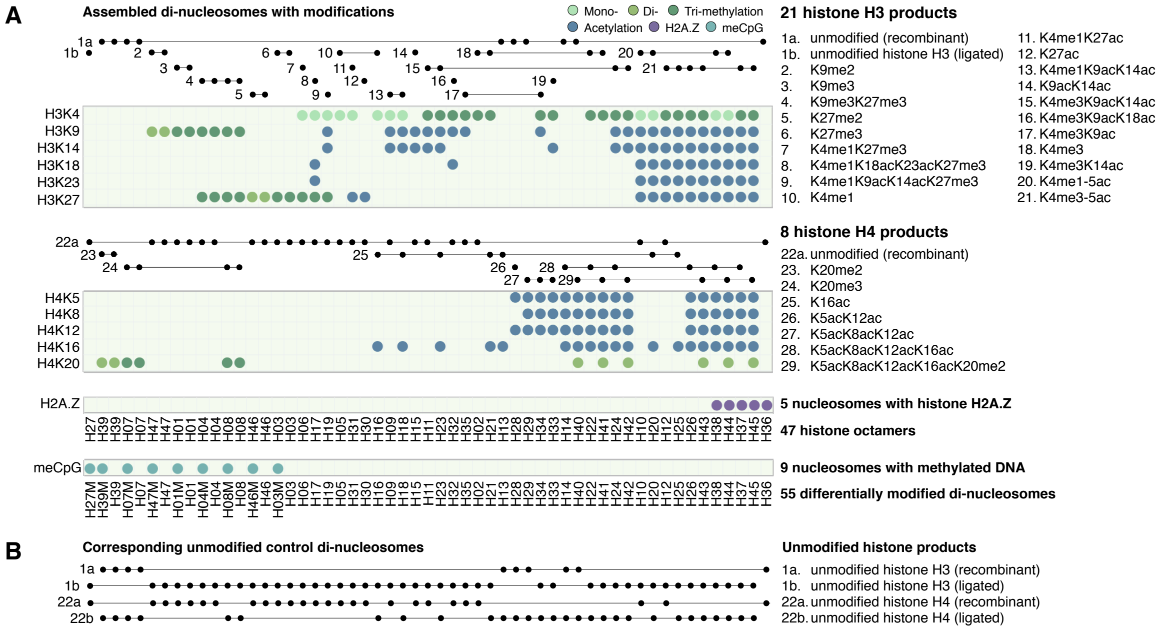

Design of MARCS di-nucleosome library

(A) 21 histone H3 and 8 histone H4 products decorated with different modifications were refolded into 47 distinct histone octamers (H01-H47) together with histones H2A and H2B as indicated. In some cases, canonical H2A was replaced by the histone variant H2A.Z. Octamers were then assembled into a library of 55 di-nucleosomes together with unmethylated or CpG-methylated (M) DNA. Repressive nucleosomes were designed to contain di- and tri-methylation of lysines 9 and 27 of histone H3 (H3K9me2/3 and H3K27me2/3), that mark constitutive and facultative heterochromatin, and tri-methylation of lysine 20 of histone H4 (H4K20me3) that marks pericentric and telomeric heterochromatin[1,2]. Combinations of these tri-methyl marks and CpG-methylated DNA were used to test for cross-talk between silencing pathways, and between heterochromatic histone modifications and DNA methylation. Euchromatic nucleosomes were decorated with mono- and tri-methylation of lysine 4 of histone H3 in order to profile enhancer- (H3K4me1) and promoter- (H3K4me3) like chromatin states. Both were combined with varying degrees of acetylation of lysines (Kac) in the H3 and H4 N-terminal tails, and the histone variant H2A.Z due to its emergent role at enhancers and promoters[3]. Di-methylation of lysine 20 of histone H4 (H4K20me2) was added to a subset of both repressive and activating nucleosomes as it is a pervasive modification present throughout the genome[4]. In addition to repressive and activating modification signatures H4K5acK12ac that marks newly deposited histones[5,6], H2A.Z that also has a function as a regulator of heterochromatin boundaries[7], and three nucleosomes combining H3K4me1 with H3K27me3 and low levels of H3 acetylation were included, as such species were detected by mass spectrometry[8,9]. In total 55 di-nucleosomes were assembled. Each of the di-nucleosomes was assigned an identifier as listed below the heatmap, see also Supplementary Table 1. The heatmap shows only modified nucleosomes. (B) Each SILAC nucleosome affinity purification experiment was controlled with an unmodified nucleosome. The histone H3 and H4 products 1a and 22a, respectively, indicate unmodified recombinant (not ligated) H3/H4 proteins that were used in controls for experiments that did not test modifications on H3 or H4. In experiments that profiled H3 and/or H4 modifications, control nucleosomes were constructed with unmodified H3 and/or H4 histone products (1b and 22b, respectively) prepared by native chemical ligation, as appropriate.

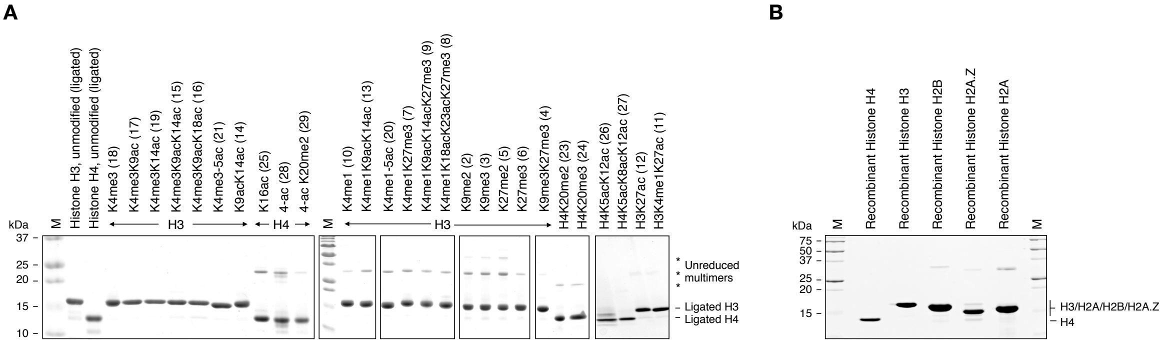

SDS-PAGE of ligated and recombinant histone products

(A) Truncated human H3Δ1-31T32C and H4Δ1-28I29C proteins were purified from *E. coli* and ligated to synthetic modified histone H3 (1-31) or H4 (1-28) thioester peptides (respectively) as described in the methods. Coomassie-stained SDS-PAGE gels of the resulting ligated histone products are shown here. Numbers in parentheses correspond to the notation in the section *Design of MARCS di-nucleosome library*. Bands marked with an asterisk correspond to unreduced multimers of histones H3 or H4. Marker (BioRad Precision Plus) lanes are marked as "M". (B) Coomassie-stained SDS-PAGE gels of purified recombinant histone proteins. Marker (BioRad Precision Plus) lanes are marked as "M".

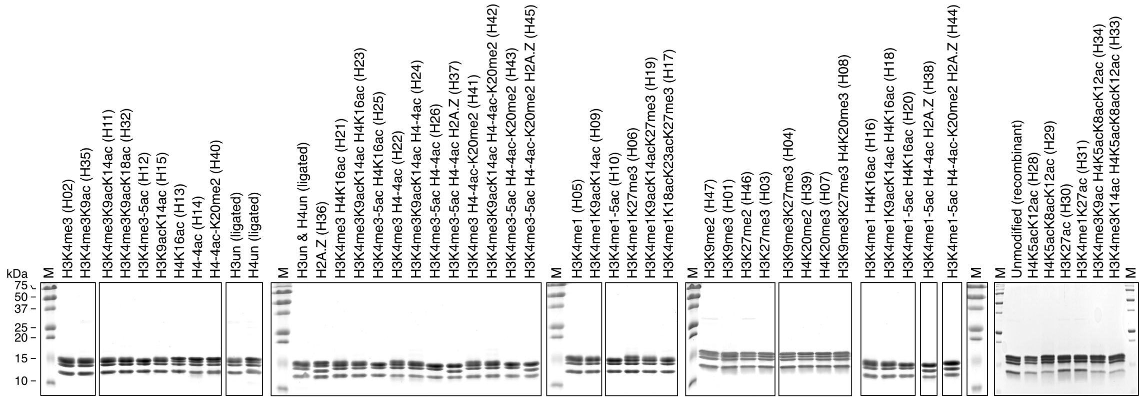

SDS-PAGE of refolded histone octamers

Histone octamers were refolded from purified histones H2A (or H2A.Z) and H2B, and either recombinant (unmodified) or ligated modified histones H3 and H4 and purified by size exclusion chromatography as described in the methods. The 'HXX' notation in parentheses corresponds to the octamer identifiers as in the section *Design of MARCS di-nucleosome library*. Lanes marked 'H3un', 'H4un', and 'H3un&H4un' correspond to octamers containing unmodified ligated H3 and/or H4 proteins that were used as control nucleosomes in experiments with H3, H4, and H3 & H4 modifications respectively. Marker lanes are marked as "M" (BioRad Precision Plus).

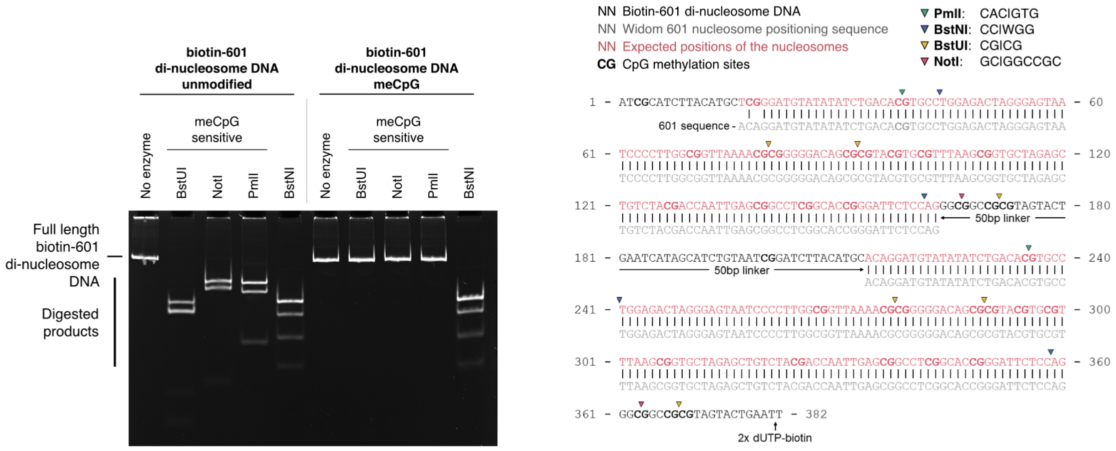

Unmethylated and CpG-methylated biotin-601 di-nucleosome DNA

Biotinylated nucleosomal DNA containing two 601 nucleosome positioning sequences[10] separated by a 50-base-pair (bp) linker was prepared in unmethylated or CpG-methylated form as described in the methods. The biotin-601 di-nucleosome DNAs were digested with methylation-sensitive restriction enzymes and separated on a 12% PAGE gel as indicated. The complete block of digestion by BstUI, NotI, and PmlI indicates successful methylation of CpG-dinucleotides in the CpG-methylated DNA. The BstNI restriction enzyme is not methylation-sensitive. Positions of undigested full-length DNA and digestion products are shown in the left panel. The DNA sequence and its alignment to the Widom 601 DNA sequence, as well as CpG methylation and restriction enzyme sites are shown in the right panel.

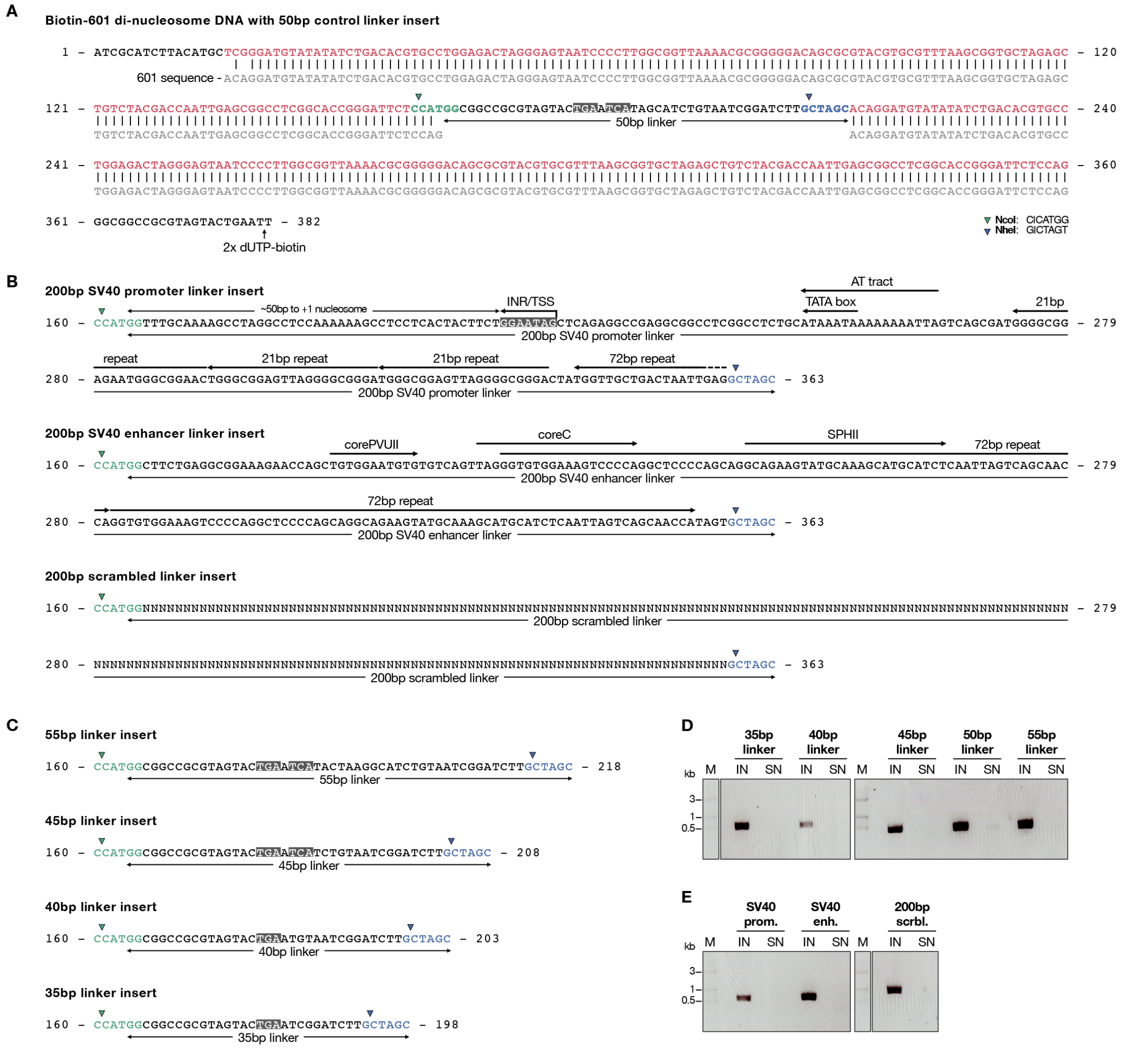

Biotin-601 di-nucleosome DNAs incorporating different linker lengths

(A) Sequence of the biotin-601 di-nucleosome DNA with 50bp control linker used as reference for pull-downs with di-nucleosomes containing different linker lengths. Alignment to the Widom 601 nucleosome positioning sequence and the expected nucleosome positions are indicated in grey and red (see also section *Unmethylated and CpG-methylated biotin-601 di-nucleosome DNA*). For facilitating the insertion of different linker sequences NcoI and NheI sites were incorporated at the 5'- and 3'-ends of the linker of the original 601 di-nucleosome DNA construct (marked in bold). The palindromic consensus motif of AP-1 family transcription factors is indicated by grey boxes. (B) Sequences of the 200bp inserts providing 'nucleosome-free regions' (NFR) between the NcoI and NheI sites of the biotin-601 di-nucleosome DNA as in (A). The 'SV40 promoter' linker incorporates the SV40 early core promoter including the three '21bp repeats', the AT tract with the TATA box, and the transcription start site (TSS). The initiation region (INR) of the early SV40 mRNA transcripts (indicated by a grey box) was placed ~50bp away from the 5'-nucleosome to mimic the canonical TATA box promoter architecture. The 'SV40 enhancer' linker contains the functional elements of the SV40 early enhancer including the 'corePVUII', 'coreC', and 'SPHII' elements, and the two '72bp repeats' as indicated. The '200bp scrambled' linker incorporates a randomized library of 192bp of DNA between the NcoI and NheI sites (see methods) thereby providing 200bp of control DNA without sequence bias between the two nucleosomes. (C) Sequences of short linker inserts ranging from 35-55bp between the NcoI and NheI sites in addition to the 50bp linker shown in (A). The palindromic consensus motif of AP-1 family

transcription factors is indicated by grey boxes. Only half of the motif remains in the shorter 40bp and 35bp linkers. (D, E) The biotinylated 601 di-nucleosome DNAs shown in panels (A-C) were prepared as described in the methods, and purity and biotinylation of the DNAs were verified by agarose gel electrophoresis of inputs (IN) and supernatants (SN) after depletion with streptavidin sepharose beads. Panel (D) shows the biotin-601 di-nucleosome DNAs with short linkers and panel (E) shows the biotin-601 di-nucleosome DNAs with the 200bp SV40 promoter, SV40 enhancer, or scrambled linker inserts. All samples were run on the same gel, but lanes were rearranged for clarity. DNA size markers (1kb Marker – Invitrogen) are labelled “M”.

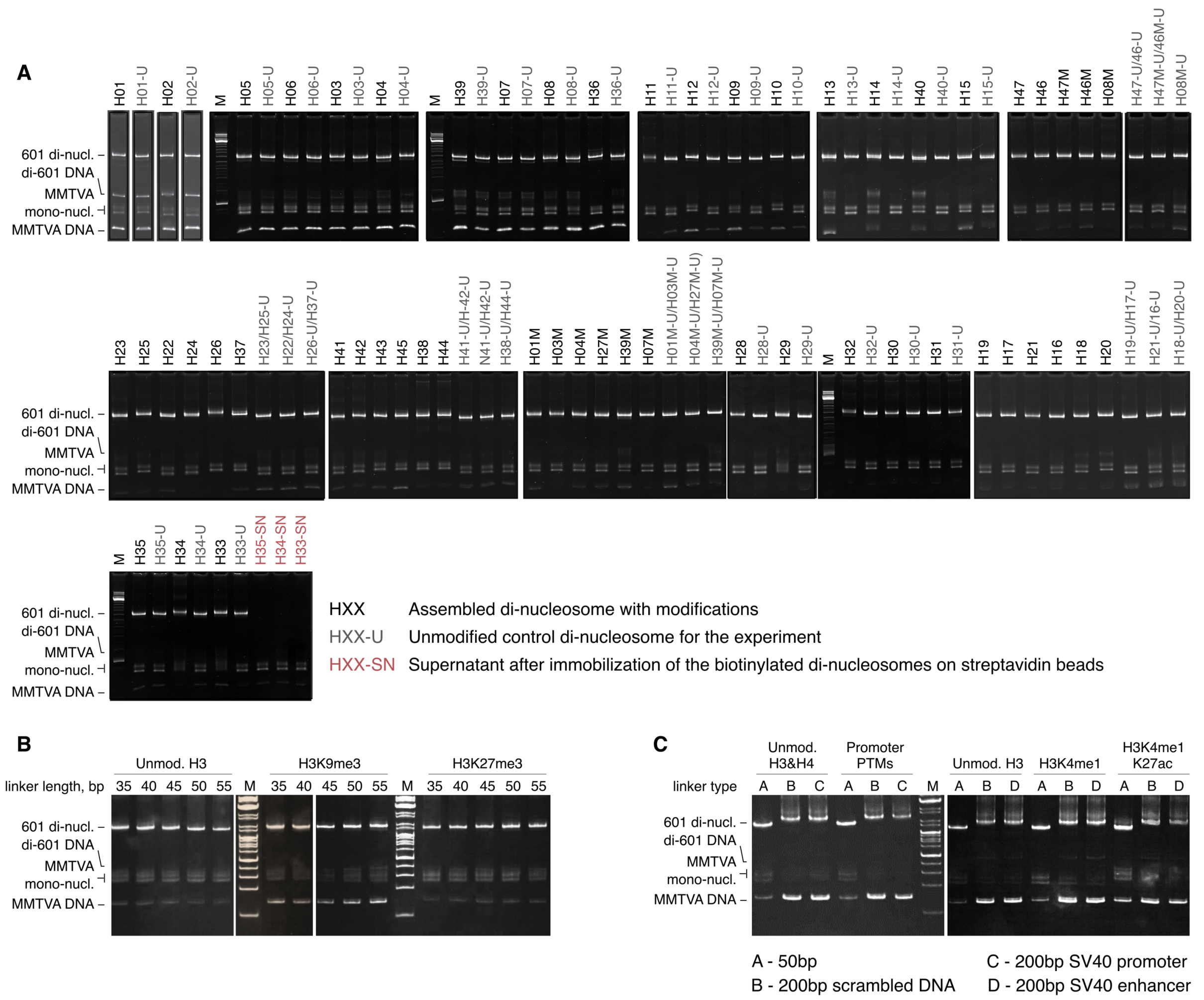

Native PAGE EMSA gels of reconstituted di-nucleosomes

(A) Biotinylated 601 di-nucleosomes with 50bp linkers used for SILAC nucleosome affinity purifications. The di-nucleosomes were assembled in the presence of a 147 bp mouse mammary tumor virus A (MMTVA) competitor DNA[11] and a slight excess of octamers to ensure saturation of the 601 repeats as described in the methods. The reconstitution reactions were separated on 5% native PAGE gels[12] to verify the integrity of the di-nucleosomes. The migration profiles of free biotin-601 di-nucleosome DNA (di-601 DNA) and competitor DNA (MMTVA DNA) and of the assembled 601 di-nucleosomes (601 di-nucl.), as well as small amounts of mono-nucleosomes formed from the MMTVA competitor DNA (MMTVA mono-nucl.), are shown. Lanes are numbered using the identifiers in the section *Design of MARCS di-nucleosome library*. Identifiers with the suffix “HXX-U" correspond to control (unmodified) nucleosomes used for the respective SNAP experiments. Identifiers with the suffix “HXX-SN” correspond to three exemplary supernatants after the immobilisation of the biotinylated 601 di-nucleosomes on the streptavidin beads. While the biotin-601 di-nucleosomes are captured by streptavidin (depleted from the supernatant), the non-biotinylated MMTV A competitor DNA and MMTV A competitor nucleosomes remain in the supernatant and can thereby be removed from the immobilized 601 di-nucleosomes in subsequent washing steps. Bands are labelled on the left-hand side of the gels. Markers (50bp Marker – Invitrogen) are labelled “M”. (B) 601 di-nucleosomes with short linker inserts ranging from 35-55bp used for label-free nucleosome affinity purifications with heterochromatic histone modifications. “M” labels markers (1kb ladder – Invitrogen). (C) 601 di-nucleosomes with 50bp and 200bp NFR linker inserts used for label-free nucleosome affinity purifications with promoter (H3acK4me3/H4acK20me2/H2A.Z) and enhancer modifications. “M” labels marker (1kb ladder – Invitrogen).

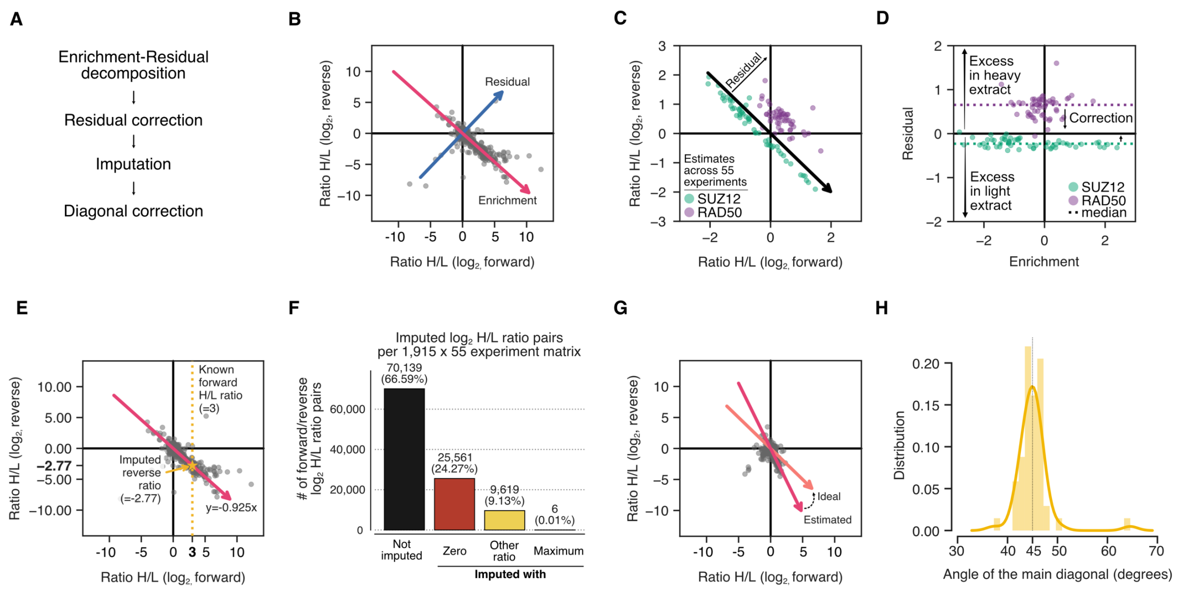

Summary of the data postprocessing algorithm for the SNAP mass spectrometry data

(A) Summary of the data postprocessing procedure for the forward and reverse log_2 H/L ratio pairs of each of the SNAP experiments. (B) First, the principal direction of data spread (i.e. direction of *enrichment*) was estimated using a modified PCA procedure as described in the Methods section. (C-D) Second, systemic variation in the second principal direction (i.e. *residual* direction) across all pull-down experiments resulting from differences in protein abundance in heavy and light cell batches was corrected to ensure a median zero offset. Note that in panels (C) and (D), unlike other panels, the scatter points correspond to data for a single protein across multiple experiments. (E) Third, in cases where only either the forward or the reverse H/L ratio was measured for the protein (9.13% of ratio pairs), but not both, the missing ratio was imputed by projecting the measured ratio to the estimated principal enrichment line. (F) Overall distribution and type of imputation across the SNAP data set. In six cases (0.01%) the estimated H/L ratio was infinite as protein intensity could be measured in the modified nucleosome, but not in the unmodified nucleosome, here the ratio was imputed to the maximum ratio identified in the particular SNAP experiment. All other missing H/L ratios were imputed to zero (24.27%). (G) Finally, the resulting data for each of the pull-down experiments were then further rotated so the estimated principal direction of variation lays exactly on the ideal 45° diagonal, so reverse ratio on average equals the negative of the forward one. (H) Estimated angle offset of the principal directions before correction, showing that for most experiments the correction had only a minor effect (see also figure in the section *Distribution of estimated enrichment axes for each of the SNAP experiment*).

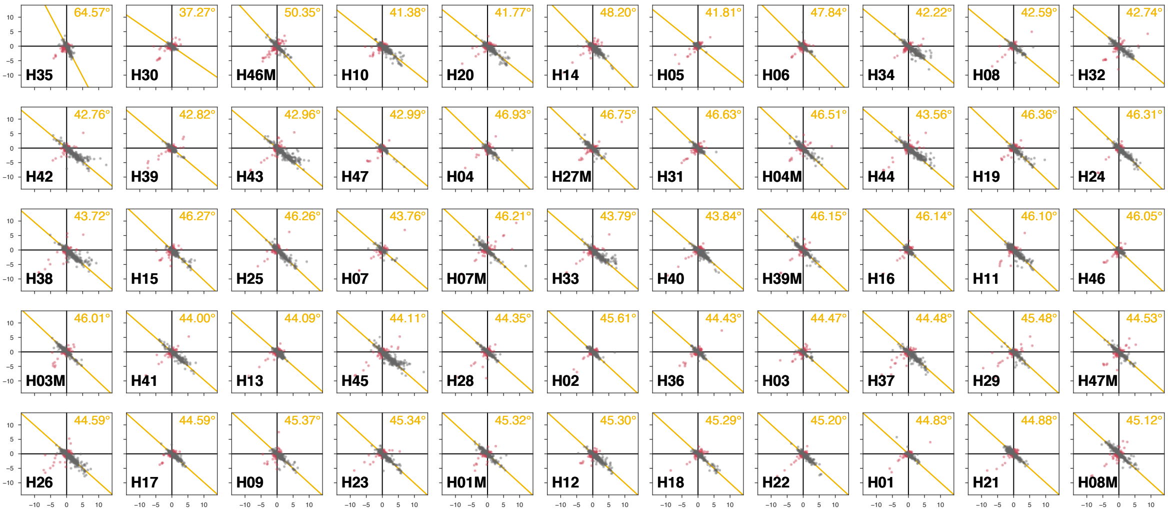

Axes: x: Ratio H/L (log_2 forward), y: (reverse)

Distribution of estimated enrichment axes for each of the SNAP experiments

The scatterplots display the raw (unprocessed) data for each of the 55 SNAP experiments, labelled with the nucleosome identifier in the bottom left corner (see Supplementary Table 1 or library design in the section *Design of MARCS di-nucleosome library* for description of the codes). Estimated principal directions of data variance are shown using yellow lines. Points coloured red were considered as outliers and not taken into account when estimating the principal direction. The angle in the top-right corner corresponds to the estimated angle offset of this line, which should ideally be equal to 45 degrees. SNAP experiments are sorted from the experiment with the biggest deviation from the ideal 45° diagonal to the lowest. The two outlier experiments for which the deviation was greatest were H35 (H3K4me3K9ac) and H30 (H3K27ac).

Verification of modification status of ligated histone products by mass spectrometry

Modification State	Peptide sequence (with specified masses of modifications)	m/z	z
**H3K4me0**	T[+119.037114]K[+56.026215]QTAR	440.2378	2+
**H3K4me1**	T[+119.037114]K[+70.041865]QTAR	447.2456	2+
**H3K4me2**	T[+119.037114]K[+28.0313]QTAR	426.2403	2+
**H3K4me3**	T[+119.037114]K[+42.04695]QTAR	433.2482	2+
**H3K9unK14un**	K[+175.063329]STGGK[+56.026215]APR	566.8091	2+
**H3K9me2K14un**	K[+147.068414]STGGK[+56.026215]APR	552.8116	2+
**H3K9me3K14un**	K[+161.084064]STGGK[+56.026215]APR	559.8195	2+
**H3K9acK14un**	K[+161.047679]STGGK[+56.026215]APR	559.8013	2+
**H3K9unK14ac**	K[+175.063329]STGGK[+42.010565]APR	559.8013	2+
**H3K9acK14ac**	K[+161.047679]STGGK[+42.010565]APR	552.7935	2+
**H3K18unK23un**	K[+175.063329]QLATK[+56.026215]AAR	609.3537	2+
**H3K18unK23ac**	K[+175.063329]QLATK[+42.010565]AAR	602.3459	2+
**H3K18acK23un**	K[+161.047679]QLATK[+56.026215]AAR	602.3459	2+
**H3K18acK23ac**	K[+161.047679]QLATK[+42.010565]AAR	595.338	2+
**H3K27unmod (lig. H3)**	K[+175.063329]SAPAC[+57.021464]GGVK[+56.026215]K[+56.026215]PHR	593.982279	3+
**H3K27me1 (lig. H3)**	K[+189.078979]SAPAC[+57.021464]GGVK[+56.026215]K[+56.026215]PHR	598.654162	3+
**H3K27me2 (lig. H3)**	K[+147.068414]SAPAC[+57.021464]GGVK[+56.026215]K[+56.026215]PHR	584.650641	3+
**H3K27me3 (lig. H3)**	K[+161.084064]SAPAC[+57.021464]GGVK[+56.026215]K[+56.026215]PHR	589.3225	3+
**H3K27ac (lig. H3)**	K[+161.047679]SAPAC[+57.021464]GGVK[+56.026215]K[+56.026215]PHR	589.310396	3+
**H3K27unmod (recomb. H3)**	K[+175.063329]SAPATGGVK[+56.026215]K[+56.026215]PHR	574.321289	3+
**H3K27me1 (recomb. H3)**	K[+189.078979]SAPATGGVK[+56.026215]K[+56.026215]PHR	578.993172	3+
**H3K27me2 (recomb. H3)**	K[+147.068414]SAPATGGVK[+56.026215]K[+56.026215]PHR	564.989651	3+
**H3K27me3 (recomb. H3)**	K[+161.084064]SAPATGGVK[+56.026215]K[+56.026215]PHR	569.661534	3+
**H3K27ac (recomb. H3)**	K[+161.047679]SAPATGGVK[+56.026215]K[+56.026215]PHR	569.649406	3+
**H4K5unK8unK12unK16un**	G[+119.037114]K[+56.026215]GGK[+56.026215]GLGK[+56.026215]GGAK[+56.026215]R	807.4597	2+
**H4K5unK8unK12unK16ac**	G[+119.037114]K[+56.026215]GGK[+56.026215]GLGK[+56.026215]GGAK[+42.010565]R	800.4519	2+
**H4K5acK8unK12acK16un**	G[+119.037114]K[+42.010565]GGK[+56.026215]GLGK[+42.010565]GGAK[+56.026215]R	793.4441	2+
**H4K5acK8acK12acK16un**	G[+119.037114]K[+42.010565]GGK[+42.010565]GLGK[+42.010565]GGAK[+56.026215]R	786.4363	2+
**H4K5acK8acK12acK16ac**	G[+119.037114]K[+42.010565]GGK[+42.010565]GLGK[+42.010565]GGAK[+42.010565]R	779.4284	2+
**H4K20unmod**	K[+175.063329]VLR	345.7185	2+
**H4K20me1**	K[+189.078979]VLR	352.7263	2+
**H4K20me2**	K[+147.068414]VLR	331.721	2+
**H4K20me3**	K[+161.084064]VLR	338.7289	2+

List of peptides used for MS-based evaluation of the ligated H3 and H4 protein quality.

The table contains a manually curated list of unmodified as well as differentially modified histone N-terminal tail peptides generated by in silico digestion of human histone H3 and H4 (column 1); peptide sequences with specified mass shifts for the modifications present (column 2); corresponding monoisotopic m/z values (column 3); and most abundant peptide charge states used for the analysis (column 4).

Histone H3 products

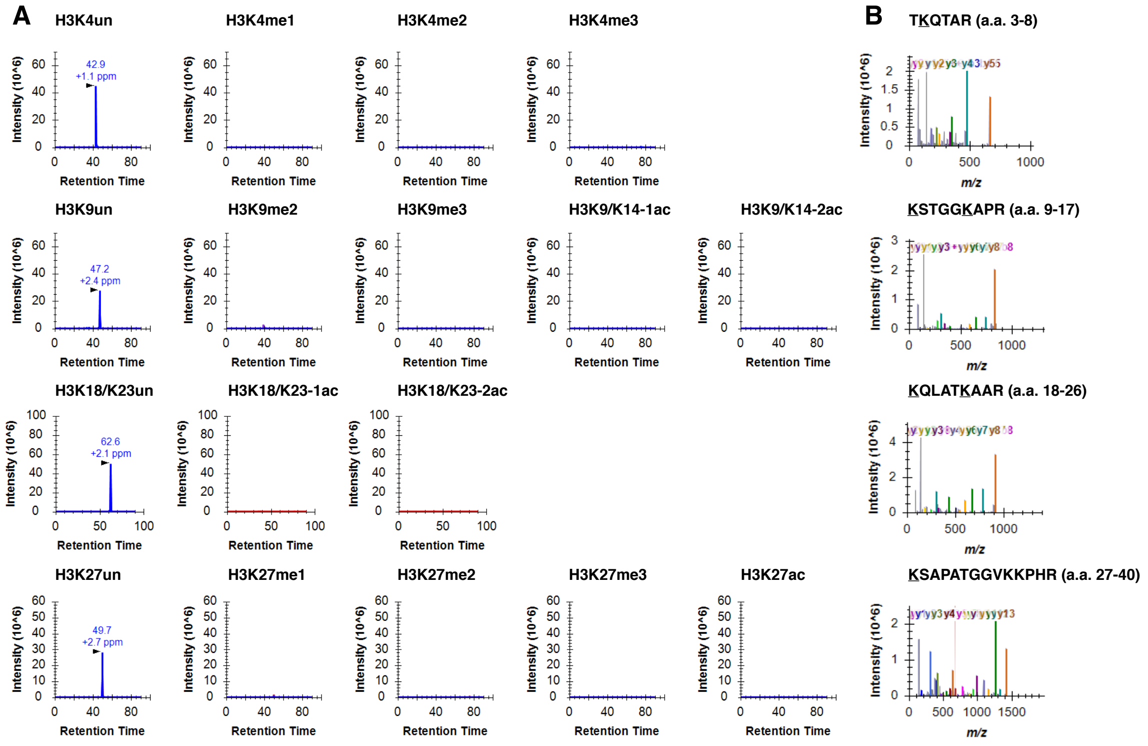

Unmodified Histone H3 (recombinant, histone product 1a)

Verification of modification status of recombinant, unmodified histone H3 protein. Histone proteins were derivatised with propionic anhydride and phenyl isocynate and analysed using liquid chromatography mass spectrometry (LC-MS) as described in the methods. Extracted ion chromatograms of 3-TKQTAR-8, 9-KSTGGKAPR-17, 18-KQLATKAAR-26 and 27-KSAPATGGVKKPHR-40 peptides at various modification levels are shown in panel A. Representative annotated MS/MS spectra are shown in panel B. Underlined lysines correspond to modification sites in panel A.

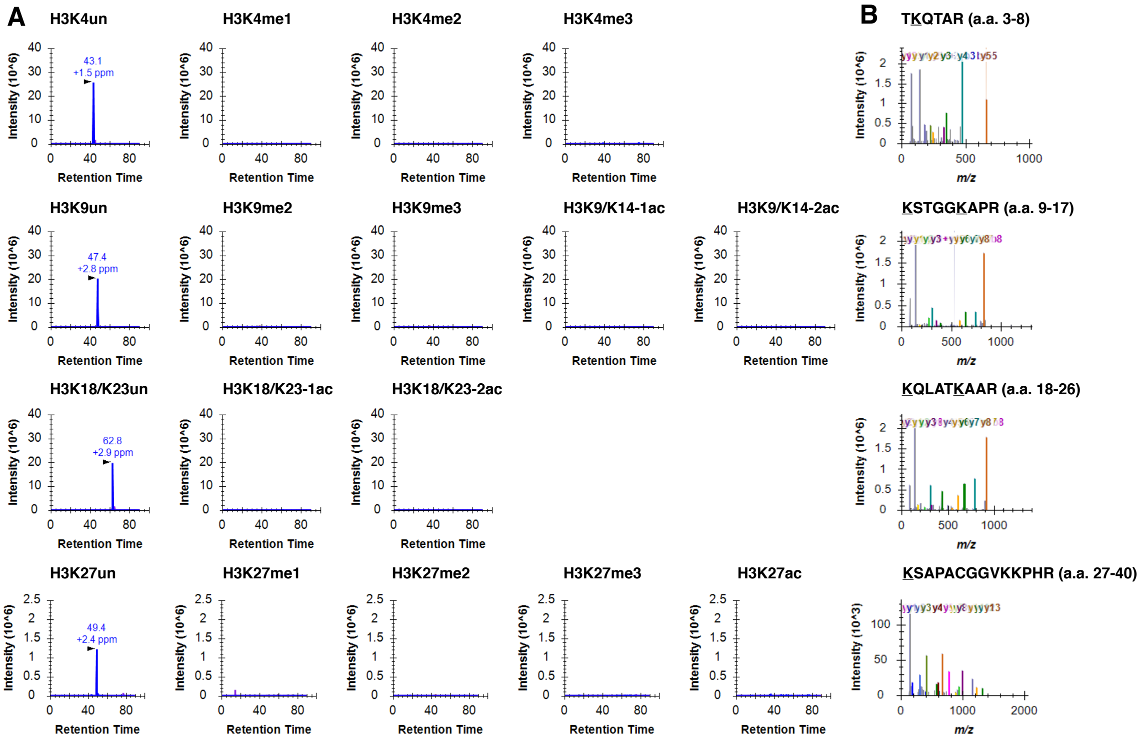

Unmodified Histone H3 (ligated, histone product 1b)

Verification of modification status of ligated, unmodified histone H3 protein. Histone proteins were derivatised with propionic anhydride and phenyl isocynate and analysed using liquid chromatography mass spectrometry (LC-MS) as described in the methods. Extracted ion chromatograms of 3-TKQTAR-8, 9-KSTGGKAPR-17, 18-KQLATKAAR-26 and 27-KSAPACGGVKKPHR-40 peptides at various modification levels are shown in panel A. Representative annotated MS/MS spectra are shown in panel B. Underlined lysines correspond to modification sites in panel A.

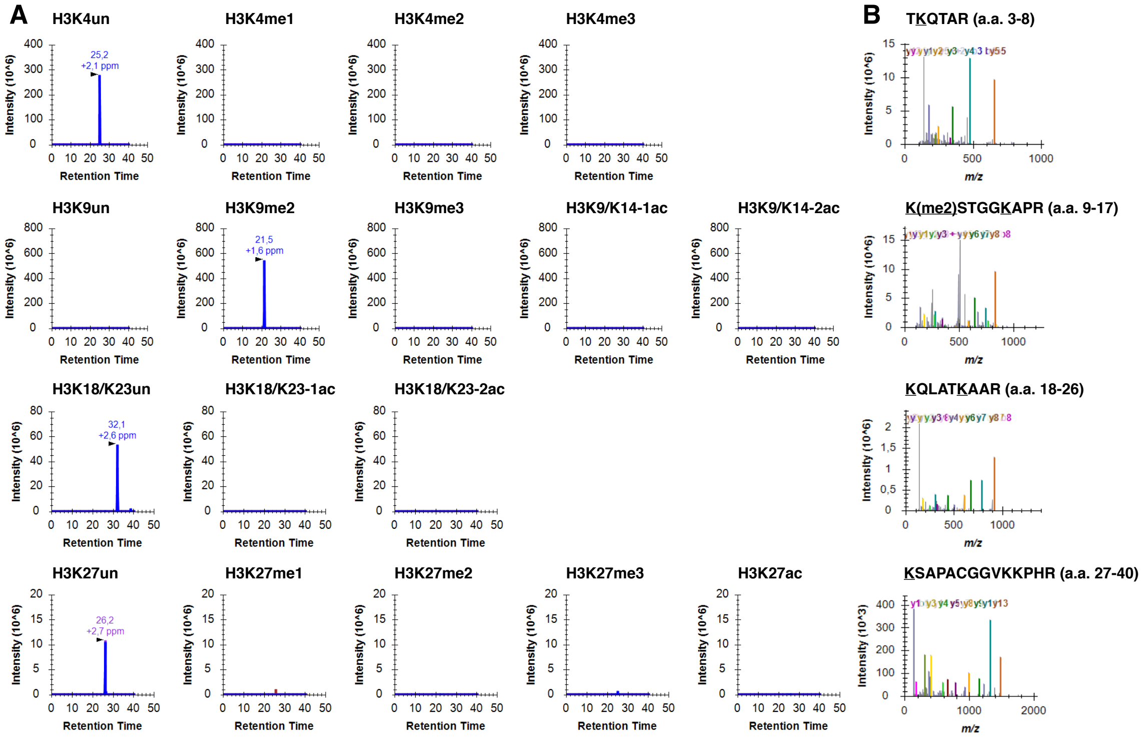

H3K9me2 (histone product 2)

Verification of modification status of ligated H3K9me2 protein. Histone proteins were derivatised with propionic anhydride and phenyl isocynate and analysed using liquid chromatography mass spectrometry (LC-MS) as described in the methods. Extracted ion chromatograms of 3-TKQTAR-8, 9-KSTGGKAPR-17, 18-KQLATKAAR-26 and 27-KSAPACGGVKKPHR-40 peptides at various modification levels are shown in panel A. Representative annotated MS/MS spectra are shown in panel B. Underlined lysines correspond to modification sites in panel A.

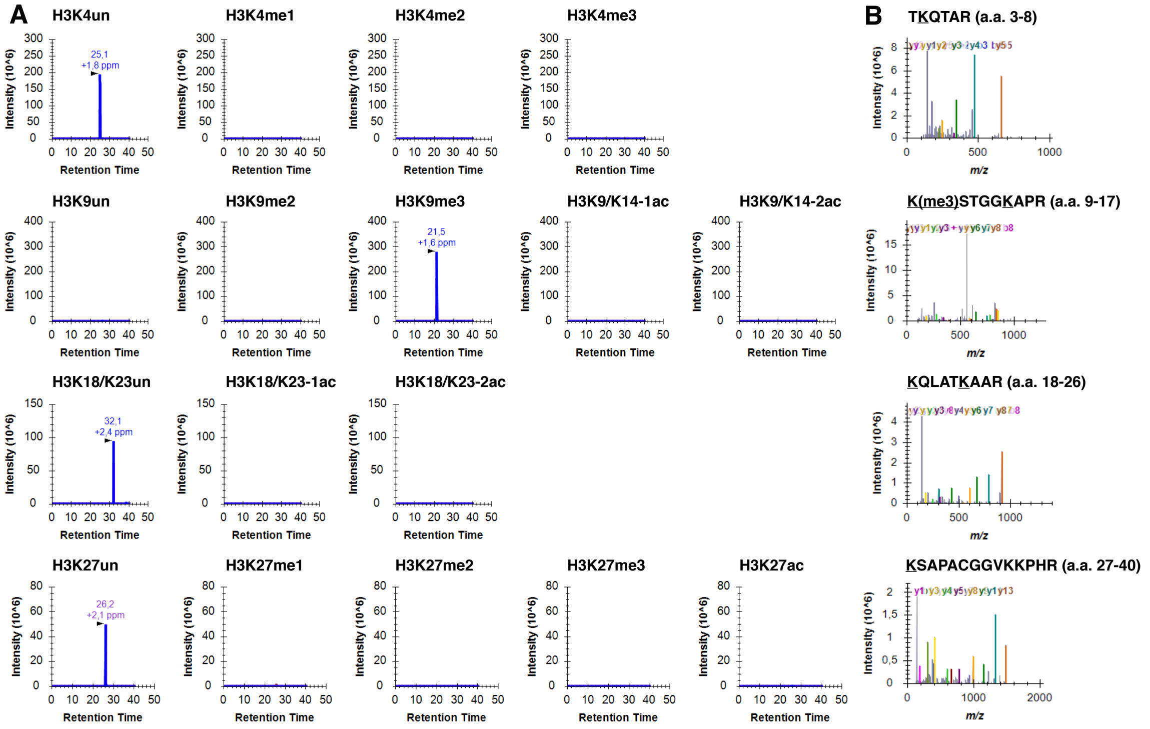

H3K9me3 (histone product 3)

Verification of modification status of ligated H3K9me3 protein. Histone proteins were derivatised with propionic anhydride and phenyl isocynate and analysed using liquid chromatography mass spectrometry (LC-MS) as described in the methods. Extracted ion chromatograms of 3-TKQTAR-8, 9-KSTGGKAPR-17, 18-KQLATKAAR-26 and 27-KSAPACGGVKKPHR-40 peptides at various modification levels are shown in panel A. Representative annotated MS/MS spectra are shown in panel B. Underlined lysines correspond to modification sites in panel A.

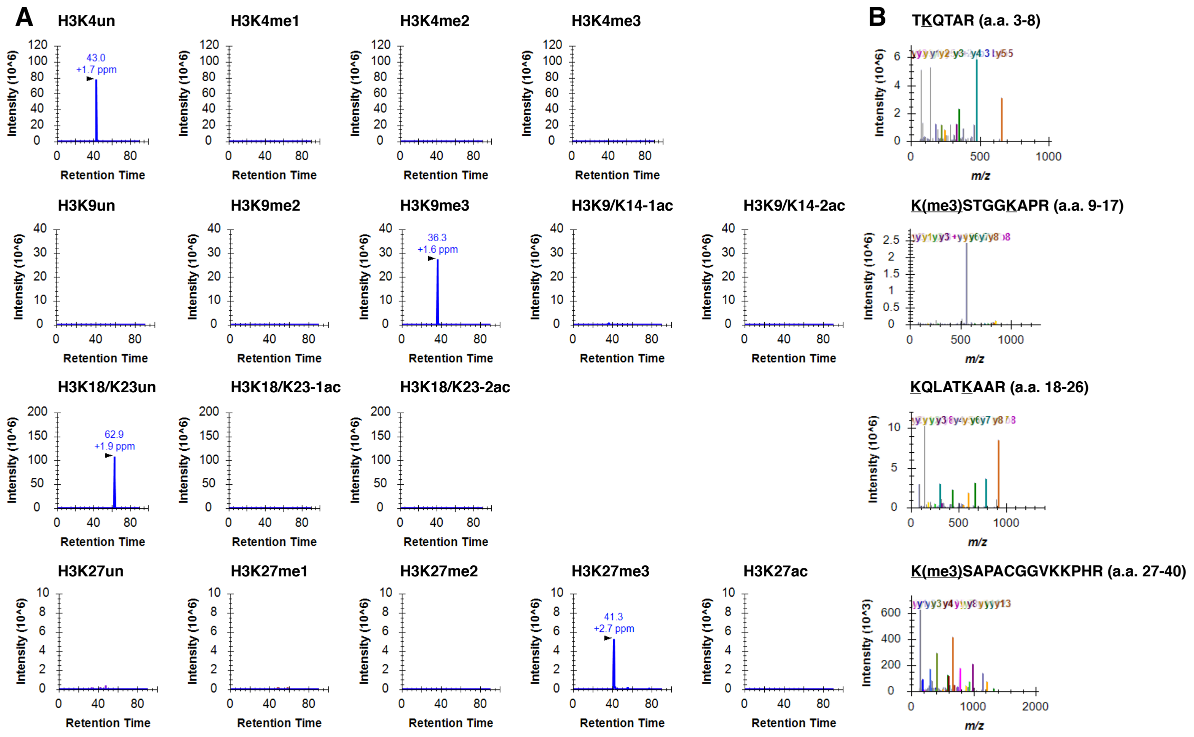

H3K9me3K27me3 (histone product 4)

Verification of modification status of ligated H3K9me3K27me3 protein. Histone proteins were derivatised with propionic anhydride and phenyl isocynate and analysed using liquid chromatography mass spectrometry (LC-MS) as described in the methods. Extracted ion chromatograms of 3-TKQTAR-8, 9-KSTGGKAPR-17, 18-KQLATKAAR-26 and 27-KSAPACGGVKKPHR-40 peptides at various modification levels are shown in panel A. Representative annotated MS/MS spectra are shown in panel B. Underlined lysines correspond to modification sites in panel A.

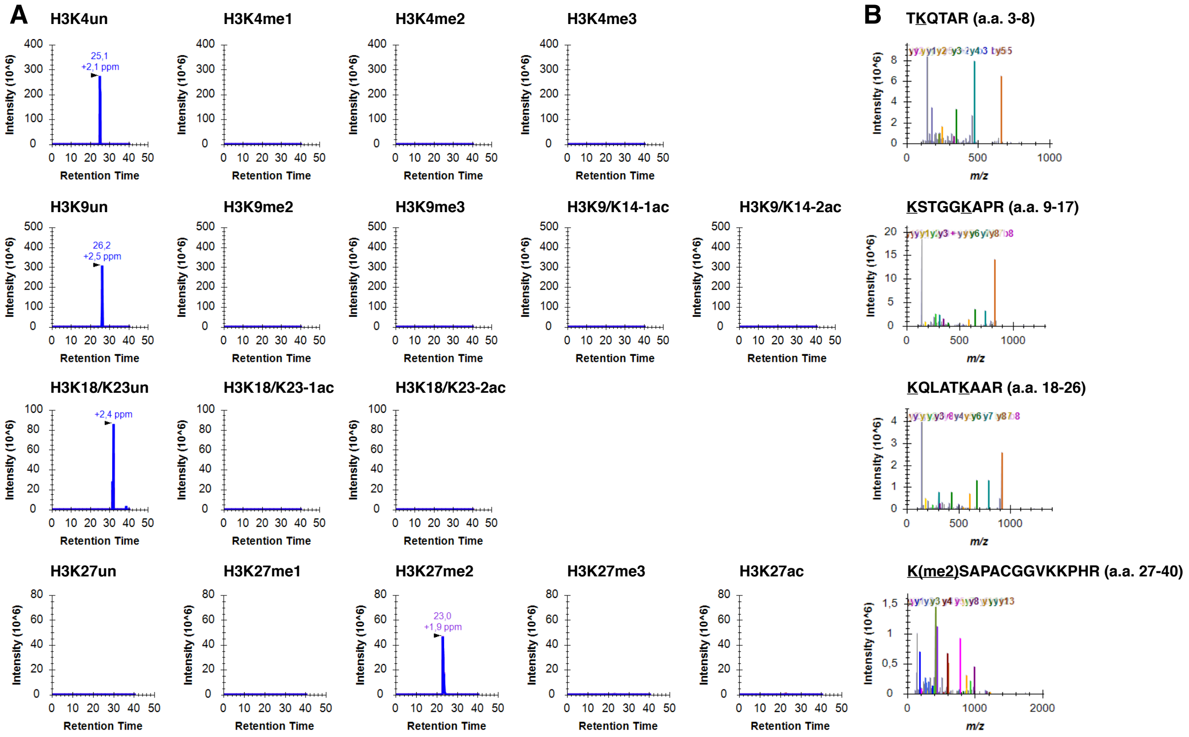

H3K27me2 (histone product 5)

Verification of modification status of ligated H3K27me2 protein. Histone proteins were derivatised with propionic anhydride and phenyl isocynate and analysed using liquid chromatography mass spectrometry (LC-MS) as described in the methods. Extracted ion chromatograms of 3-TKQTAR-8, 9-KSTGGKAPR-17, 18-KQLATKAAR-26 and 27-KSAPACGGVKKPHR-40 peptides at various modification levels are shown in panel A. Representative annotated MS/MS spectra are shown in panel B. Underlined lysines correspond to modification sites in panel A.

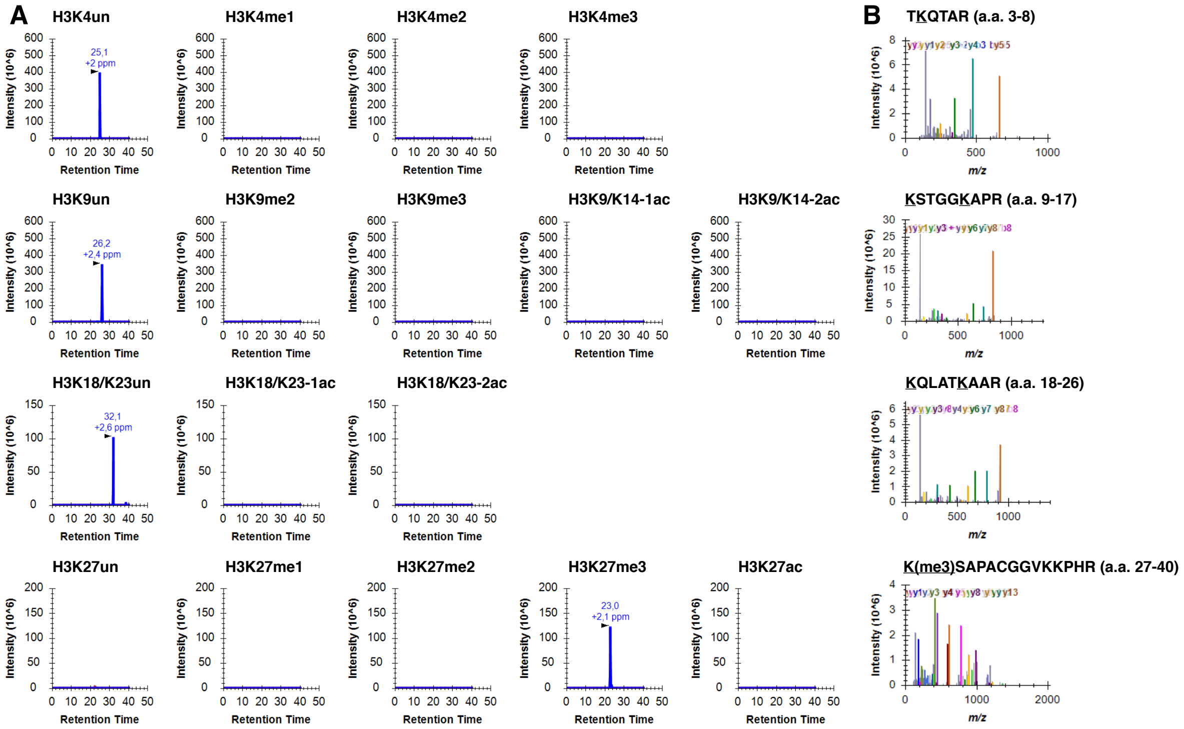

H3K27me3 (histone product 6)

Verification of modification status of ligated H3K27me3 protein. Histone proteins were derivatised with propionic anhydride and phenyl isocynate and analysed using liquid chromatography mass spectrometry (LC-MS) as described in the methods. Extracted ion chromatograms of 3-TKQTAR-8, 9-KSTGGKAPR-17, 18-KQLATKAAR-26 and 27-KSAPACGGVKKPHR-40 peptides at various modification levels are shown in panel A. Representative annotated MS/MS spectra are shown in panel B. Underlined lysines correspond to modification sites in panel A.

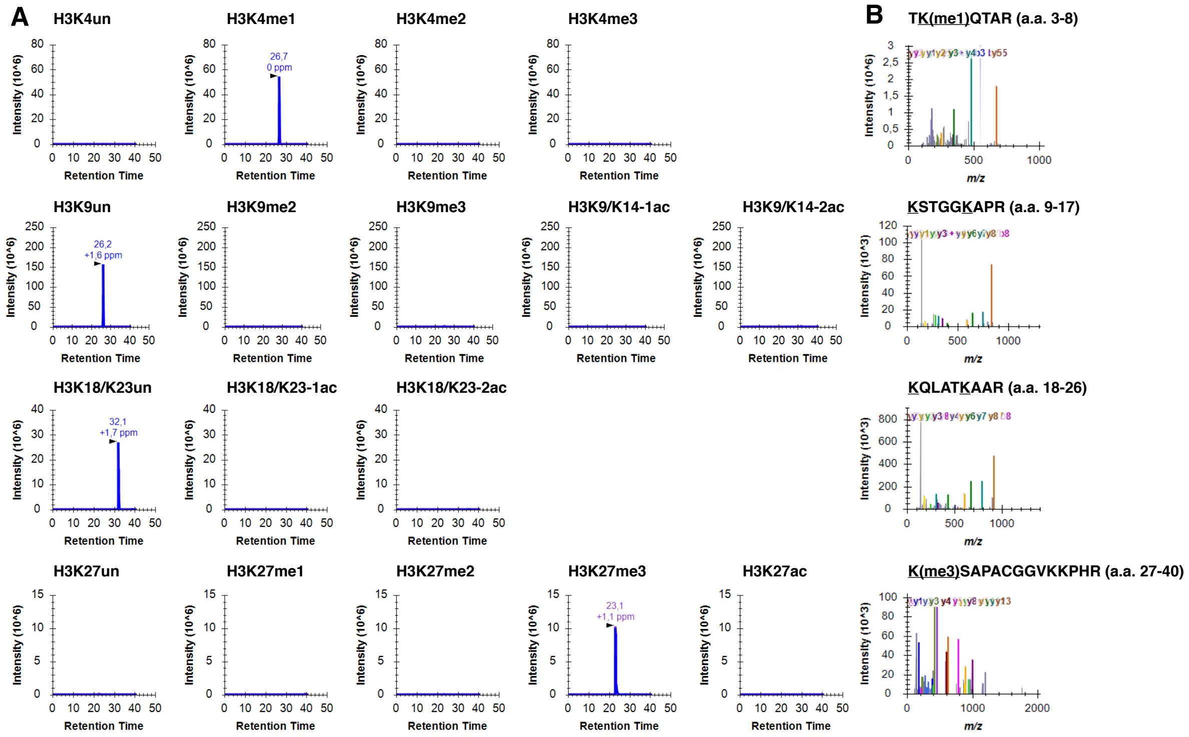

H3K4me1K27me3 (histone product 7)

Verification of modification status of ligated H3K4me1K27me3 protein. Histone proteins were derivatised with propionic anhydride and phenyl isocynate and analysed using liquid chromatography mass spectrometry (LC-MS) as described in the methods. Extracted ion chromatograms of 3-TKQTAR-8, 9-KSTGGKAPR-17, 18-KQLATKAAR-26 and 27-KSAPACGGVKKPHR-40 peptides at various modification levels are shown in panel A. Representative annotated MS/MS spectra are shown in panel B. Underlined lysines correspond to modification sites in panel A.

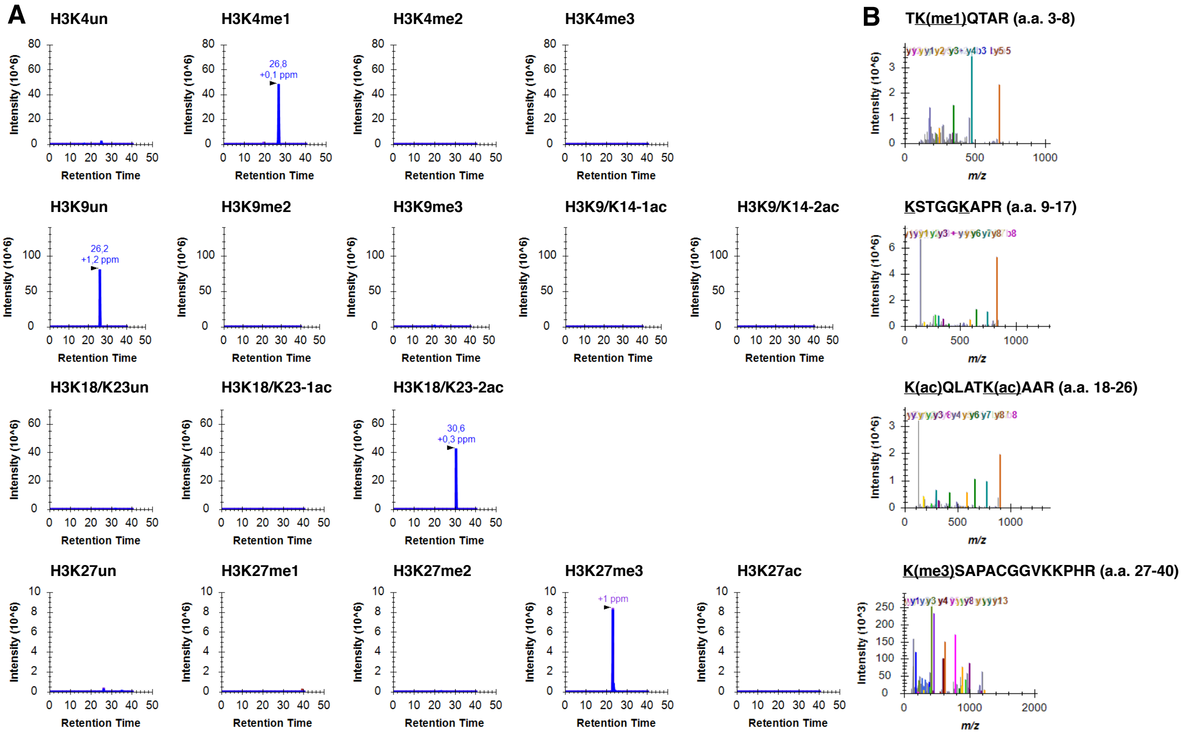

H3K4me1K18acK23acK27me3 (histone product 8)

Verification of modification status of ligated H3K4me1K18acK23acK27me3 protein. Histone proteins were derivatised with propionic anhydride and phenyl isocynate and analysed using liquid chromatography mass spectrometry (LC-MS) as described in the methods. Extracted ion chromatograms of 3-TKQTAR-8, 9-KSTGGKAPR-17, 18-KQLATKAAR-26 and 27-KSAPACGGVKKPHR-40 peptides at various modification levels are shown in panel A. Representative annotated MS/MS spectra are shown in panel B. Underlined lysines correspond to modification sites in panel A.

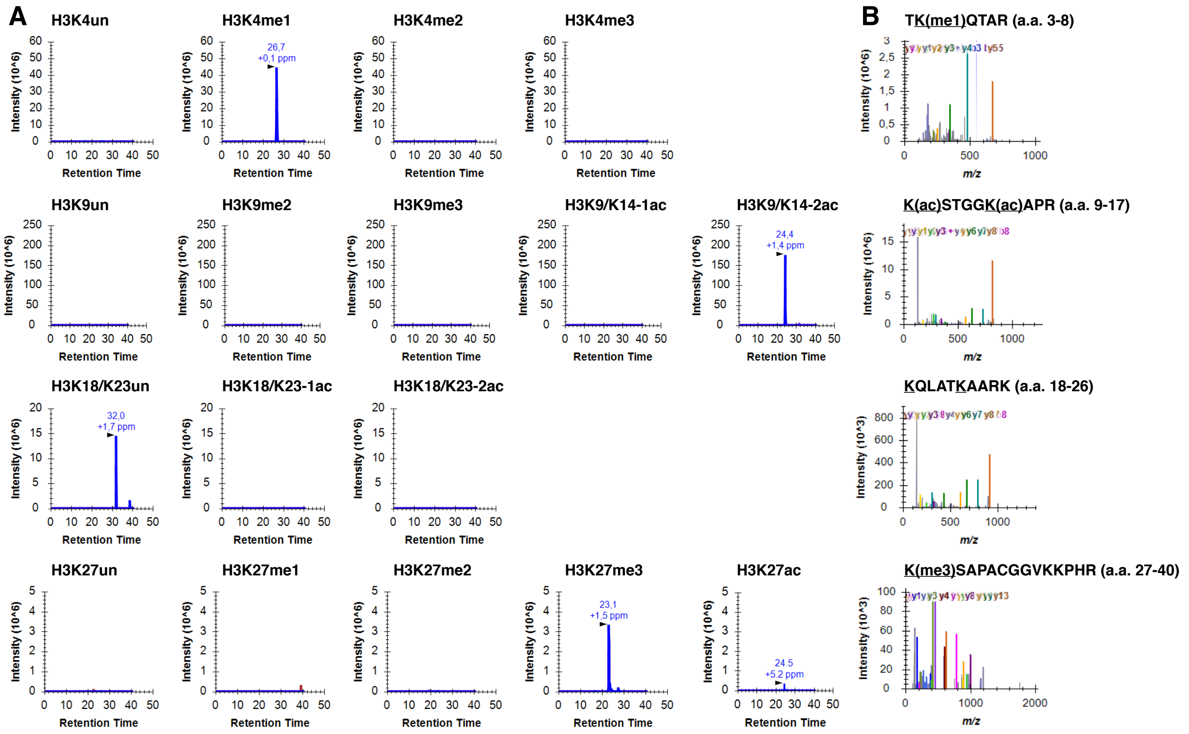

H3K4me1K9acK14acK27me3 (histone product 9)

Verification of modification status of ligated H3K4me1K9acK14acK27me3 protein. Histone proteins were derivatised with propionic anhydride and phenyl isocynate and analysed using liquid chromatography mass spectrometry (LC-MS) as described in the methods. Extracted ion chromatograms of 3-TKQTAR-8, 9-KSTGGKAPR-17, 18-KQLATKAAR-26 and 27-KSAPACGGVKKPHR-40 peptides at various modification levels are shown in panel A. Representative annotated MS/MS spectra are shown in panel B. Underlined lysines correspond to modification sites in panel A.

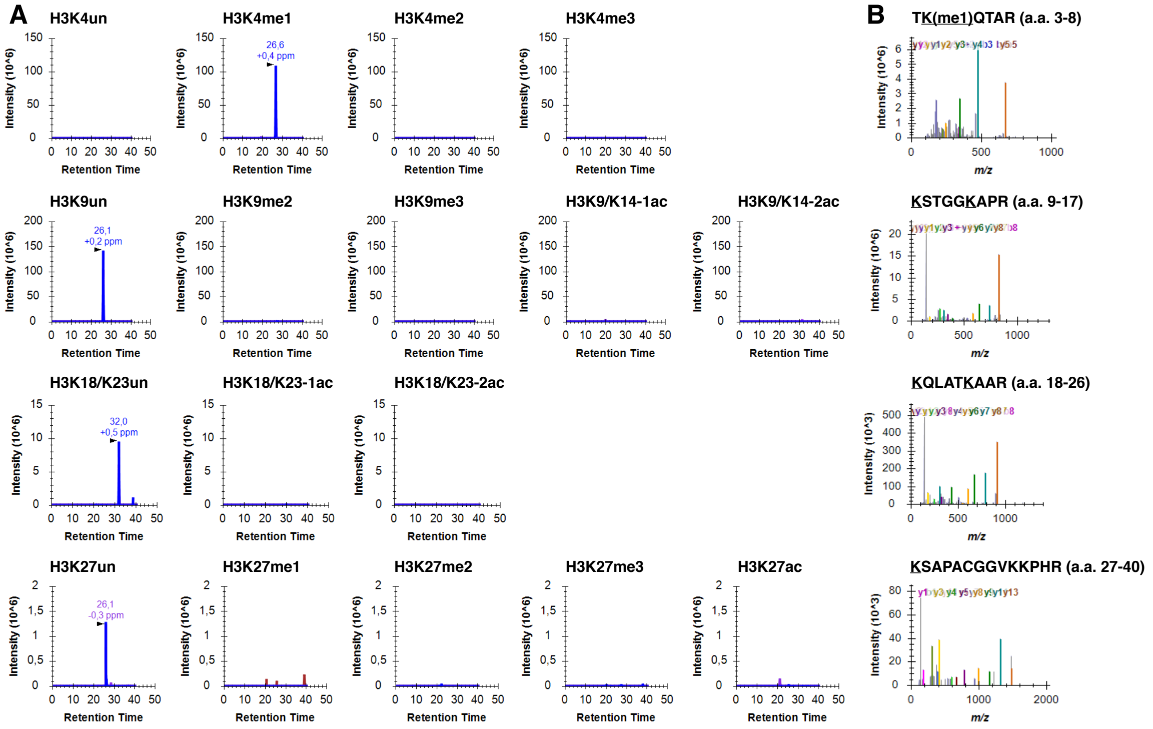

H3K4me1 (histone product 10)

Verification of modification status of ligated H3K4me1 protein. Histone proteins were derivatised with propionic anhydride and phenyl isocynate and analysed using liquid chromatography mass spectrometry (LC-MS) as described in the methods. Extracted ion chromatograms of 3-TKQTAR-8, 9-KSTGGKAPR-17, 18-KQLATKAAR-26 and 27-KSAPACGGVKKPHR-40 peptides at various modification levels are shown in panel A. Representative annotated MS/MS spectra are shown in panel B. Underlined lysines correspond to modification sites in panel A.

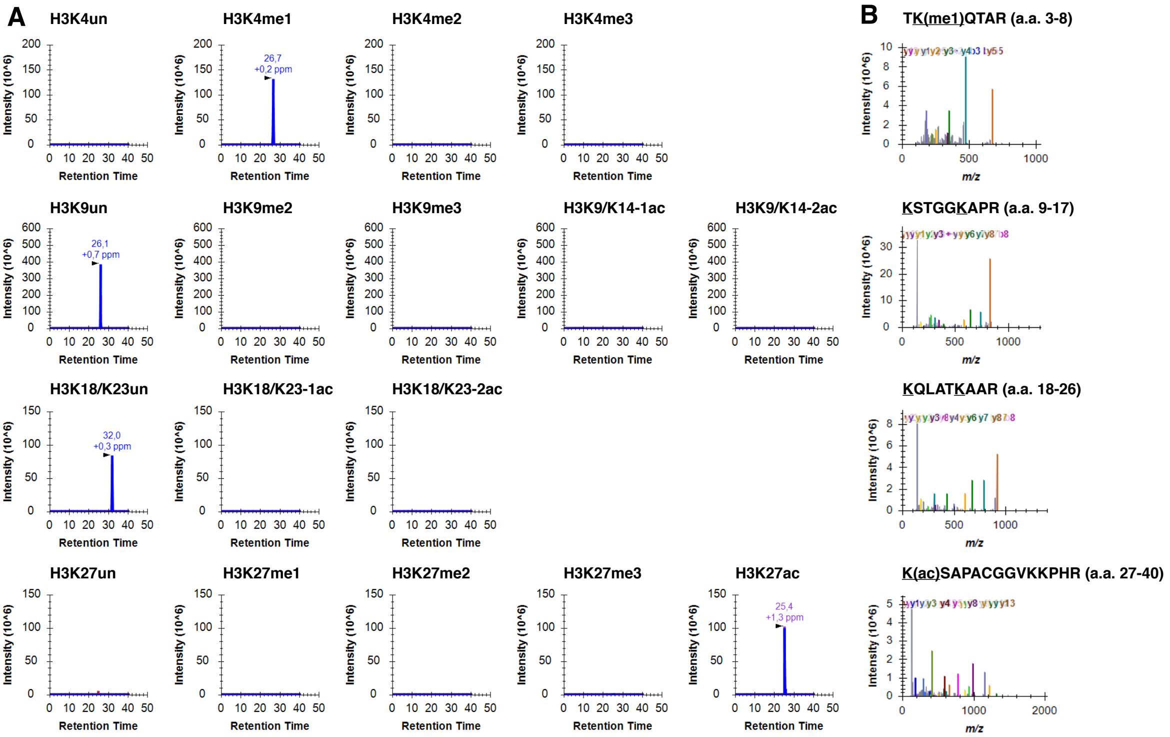

H3K4me1K27ac (histone product 11)

Verification of modification status of ligated H3K4me1K27ac protein. Histone proteins were derivatised with propionic anhydride and phenyl isocyanate and analysed using liquid chromatography mass spectrometry (LC-MS) as described in the methods. Extracted ion chromatograms of 3-TKQTAR-8, 9-KSTGGKAPR-17, 18-KQLATKAAR-26 and 27-KSAPACGGVKKPHR-40 peptides at various modification levels are shown in panel A. Representative annotated MS/MS spectra are shown in panel B. Underlined lysines correspond to modification sites in panel A.

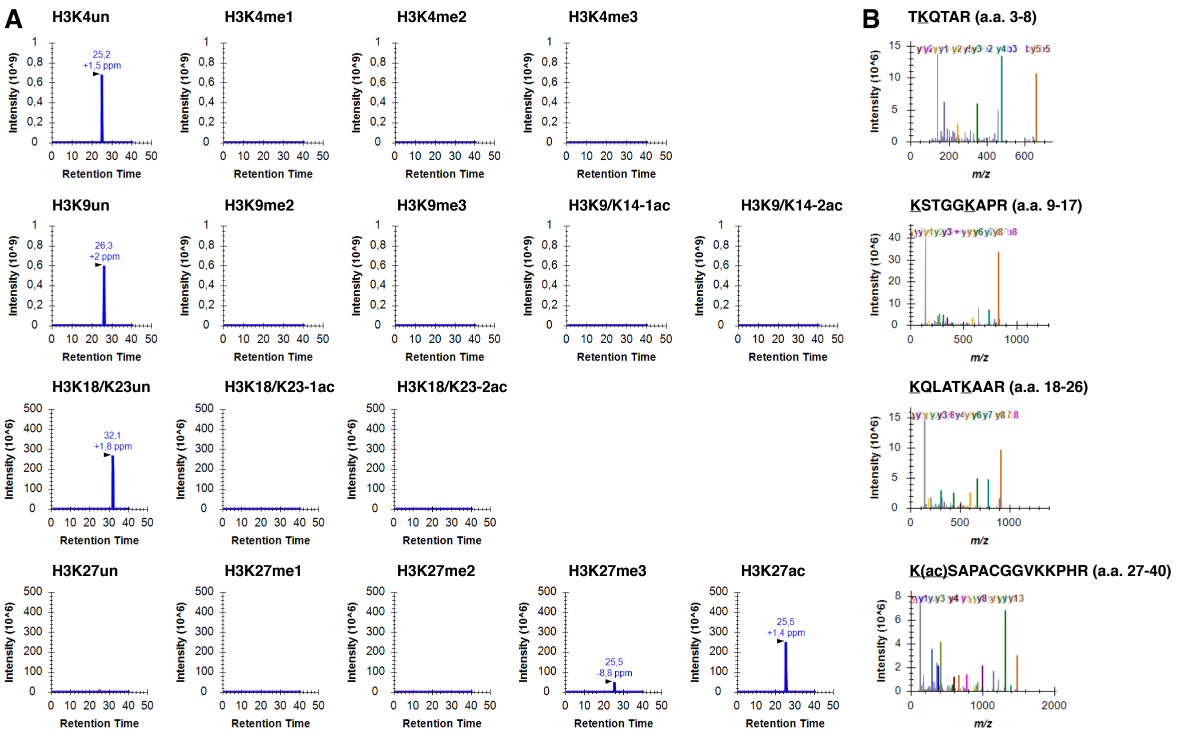

H3K27ac (histone product 12)

Verification of modification status of ligated H3K27ac protein. Histone proteins were derivatised with propionic anhydride and phenyl isocynate and analysed using liquid chromatography mass spectrometry (LC-MS) as described in the methods. Extracted ion chromatograms of 3-TKQTAR-8, 9-KSTGGKAPR-17, 18-KQLATKAAR-26 and 27-KSAPACGGVKKPHR-40 peptides at various modification levels are shown in panel A. Representative annotated MS/MS spectra are shown in panel B. Underlined lysines correspond to modification sites in panel A.

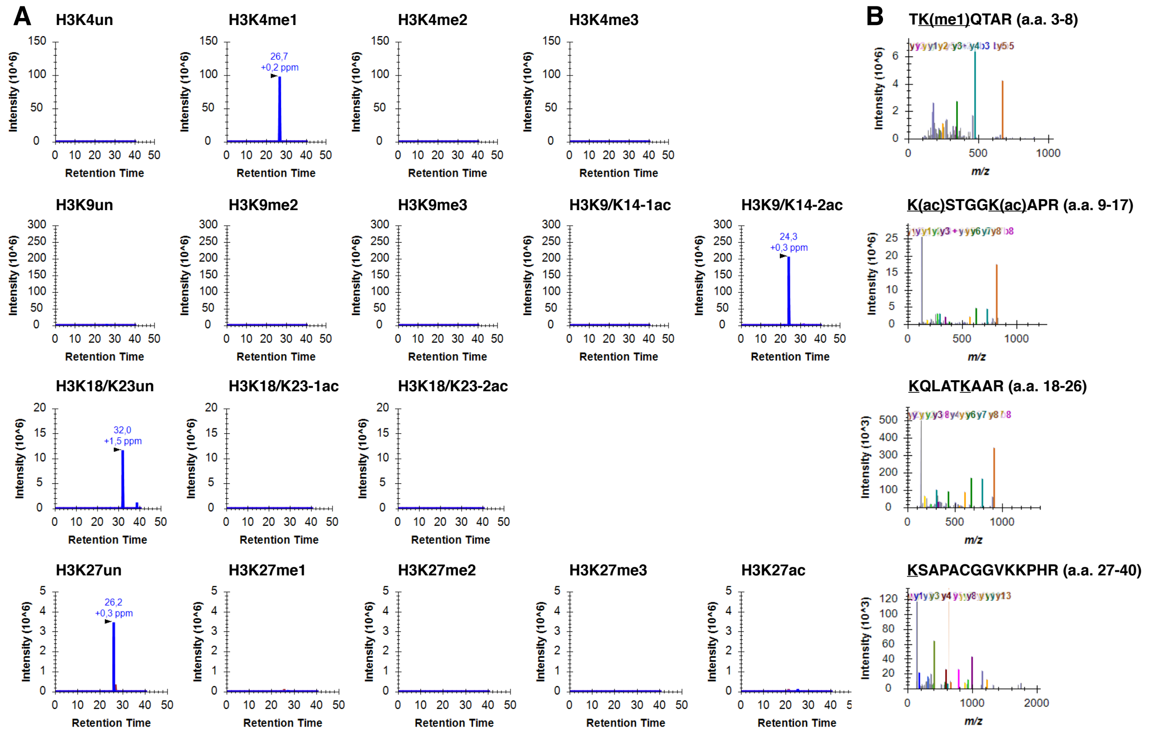

H3K4me1K9acK14ac (histone product 13)

Verification of modification status of ligated H3K4me1K9acK14ac protein. Histone proteins were derivatised with propionic anhydride and phenyl isocynate and analysed using liquid chromatography mass spectrometry (LC-MS) as described in the methods. Extracted ion chromatograms of 3-TKQTAR-8, 9-KSTGGKAPR-17, 18-KQLATKAAR-26 and 27-KSAPACGGVKKPHR-40 peptides at various modification levels are shown in panel A. Representative annotated MS/MS spectra are shown in panel B. Underlined lysines correspond to modification sites in panel A.

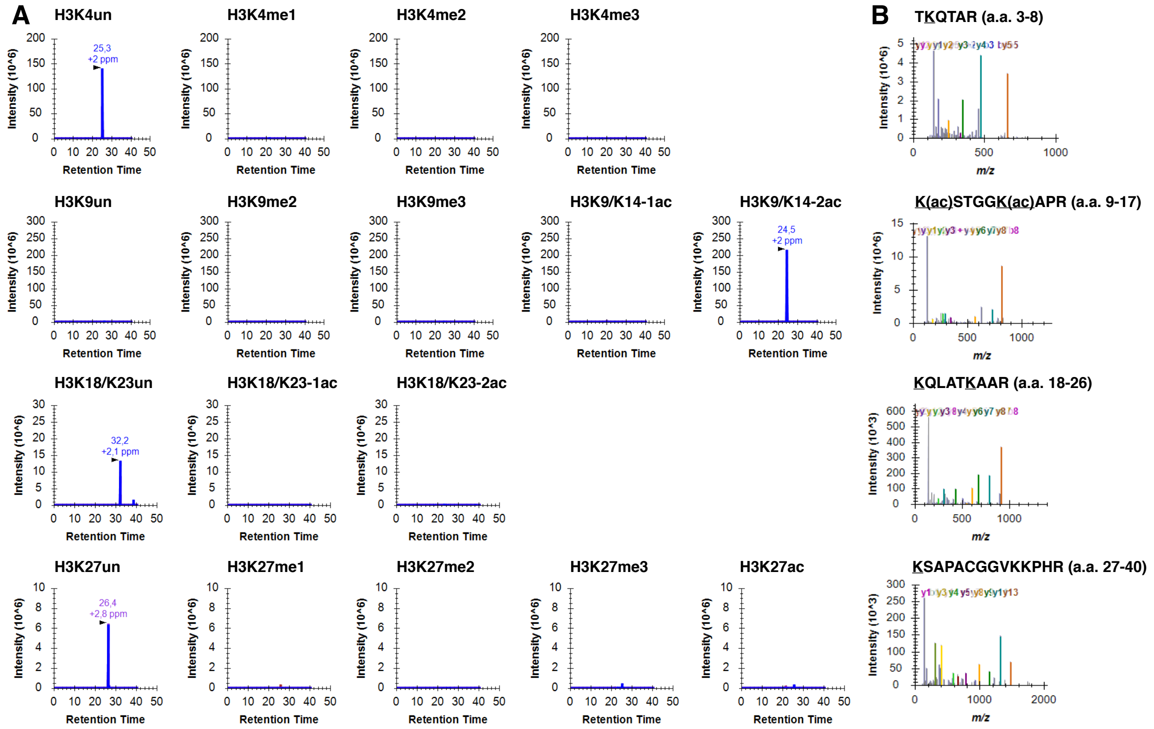

H3K9acK14ac (histone product 14)

Verification of modification status of ligated H3K9acK14ac protein. Histone proteins were derivatised with propionic anhydride and phenyl isocynate and analysed using liquid chromatography mass spectrometry (LC-MS) as described in the methods. Extracted ion chromatograms of 3-TKQTAR-8, 9-KSTGGKAPR-17, 18-KQLATKAAR-26 and 27-KSAPACGGVKKPHR-40 peptides at various modification levels are shown in panel A. Representative annotated MS/MS spectra are shown in panel B. Underlined lysines correspond to modification sites in panel A.

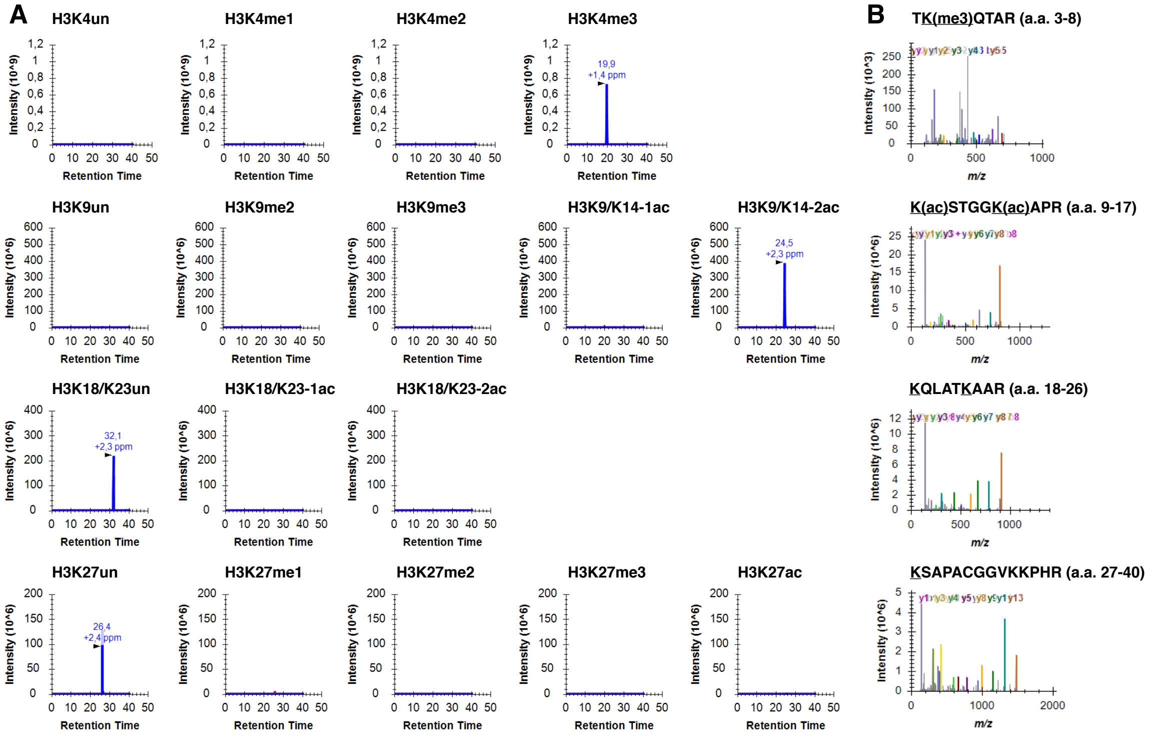

H3K4me3K9acK14ac (histone product 15)

Verification of modification status of ligated H3K4me3K9acK14ac protein. Histone proteins were derivatised with propionic anhydride and phenyl isocynate and analysed using liquid chromatography mass spectrometry (LC-MS) as described in the methods. Extracted ion chromatograms of 3-TKQTAR-8, 9-KSTGGKAPR-17, 18-KQLATKAAR-26 and 27-KSAPACGGVKKPHR-40 peptides at various modification levels are shown in panel A. Representative annotated MS/MS spectra are shown in panel B. Underlined lysines correspond to modification sites in panel A.

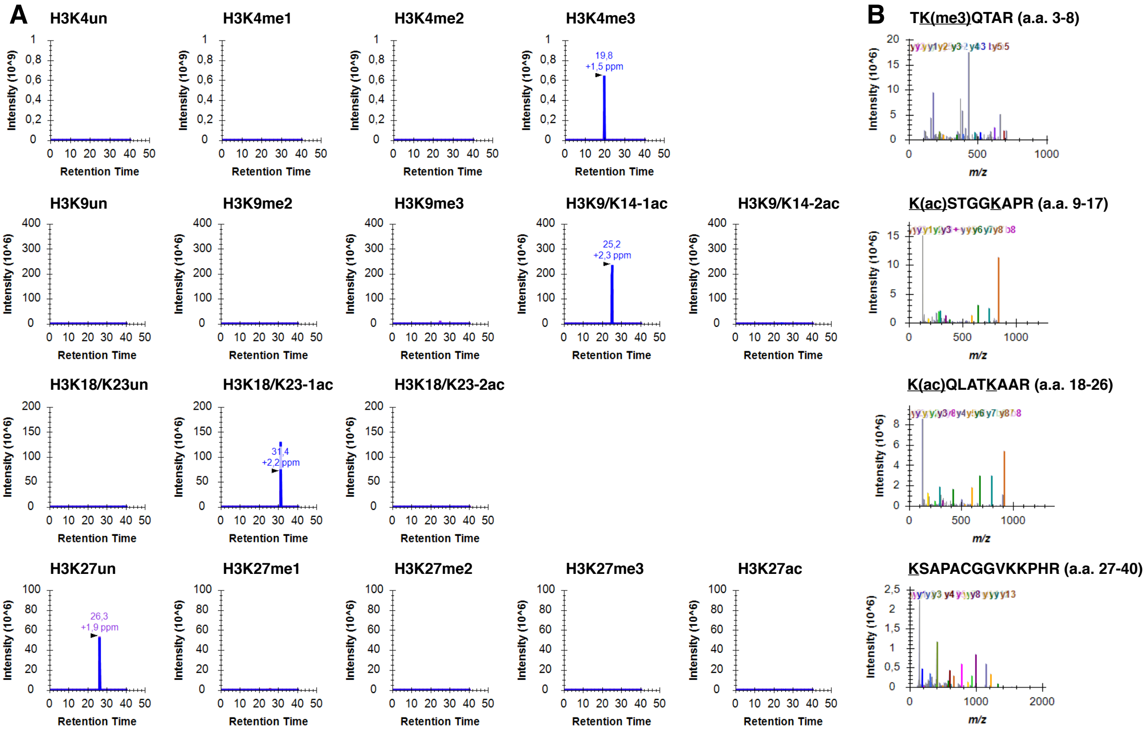

H3K4me3K9acK18ac (histone product 16)

Verification of modification status of ligated H3K4me3K9acK18ac protein. Histone proteins were derivatised with propionic anhydride and phenyl isocynate and analysed using liquid chromatography mass spectrometry (LC-MS) as described in the methods. Extracted ion chromatograms of 3-TKQTAR-8, 9-KSTGGKAPR-17, 18-KQLATKAAR-26 and 27-KSAPACGGVKKPHR-40 peptides at various modification levels are shown in panel A. Representative annotated MS/MS spectra are shown in panel B. Underlined lysines correspond to modification sites in panel A.

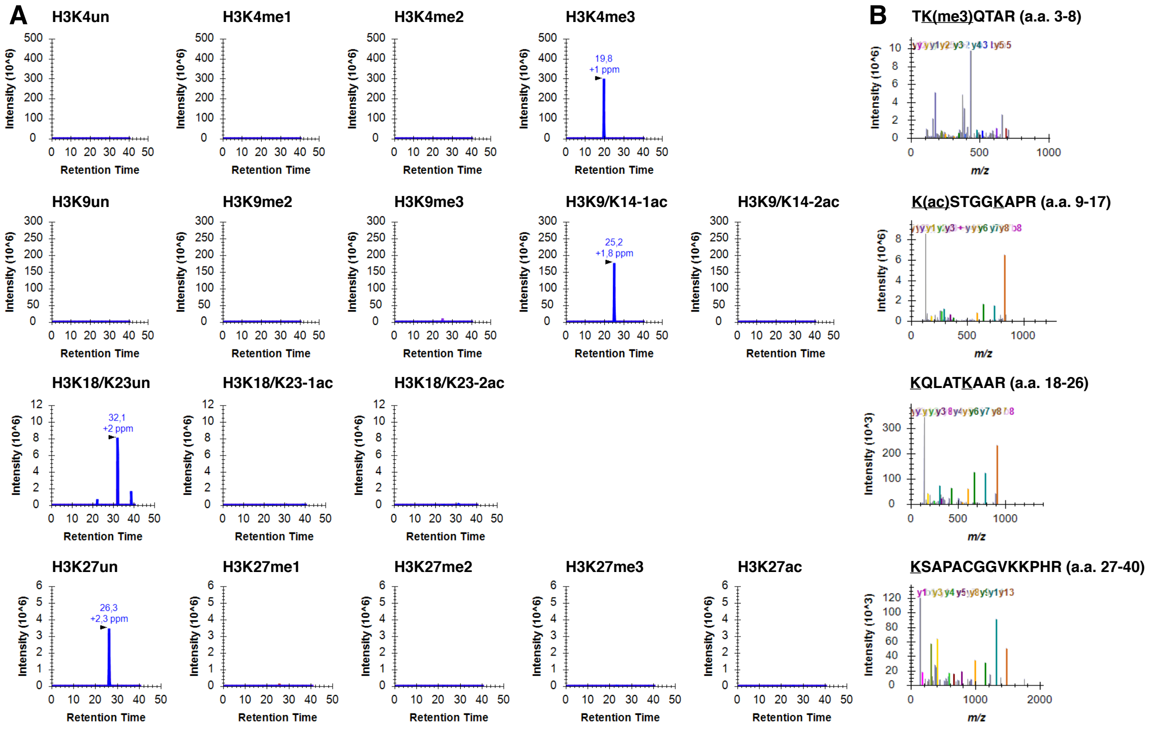

H3K4me3K9ac (histone product 17)

Verification of modification status of ligated H3K4me3K9ac protein. Histone proteins were derivatised with propionic anhydride and phenyl isocynate and analysed using liquid chromatography mass spectrometry (LC-MS) as described in the methods. Extracted ion chromatograms of 3-TKQTAR-8, 9-KSTGGKAPR-17, 18-KQLATKAAR-26 and 27-KSAPACGGVKKPHR-40 peptides at various modification levels are shown in panel A. Representative annotated MS/MS spectra are shown in panel B. Underlined lysines correspond to modification sites in panel A.

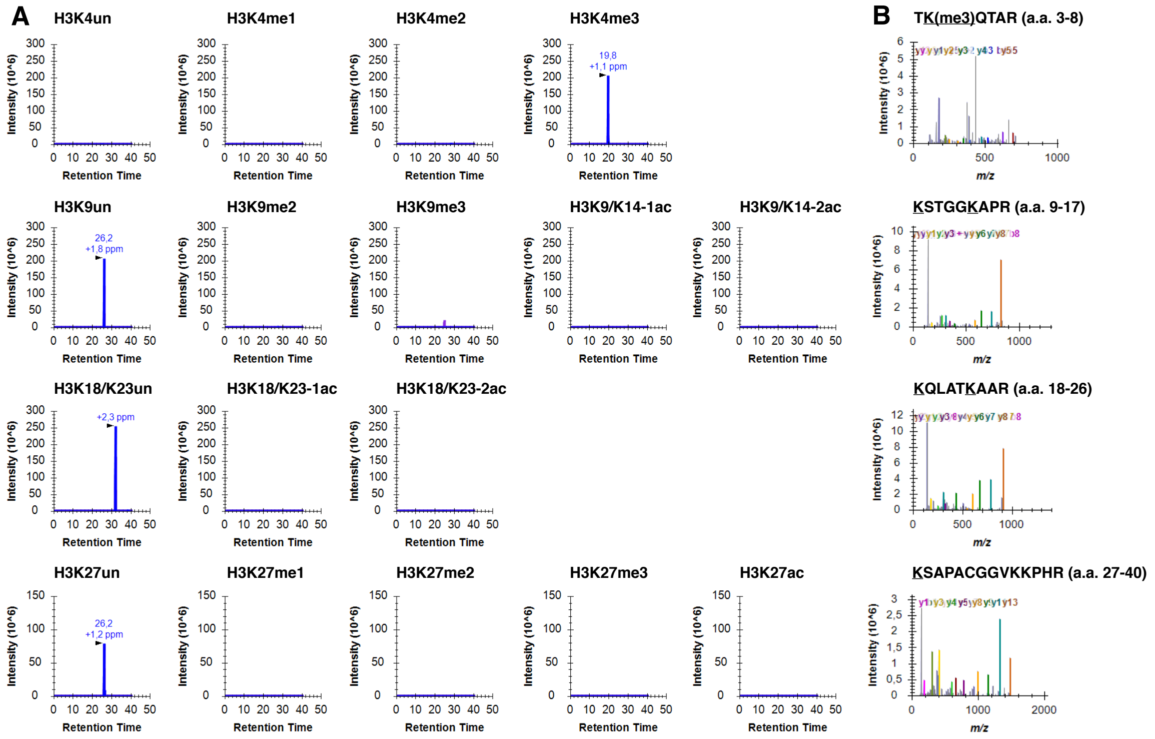

H3K4me3 (histone product 18)

Verification of modification status of ligated H3K4me3 protein. Histone proteins were derivatised with propionic anhydride and phenyl isocynate and analysed using liquid chromatography mass spectrometry (LC-MS) as described in the methods. Extracted ion chromatograms of 3-TKQTAR-8, 9-KSTGGKAPR-17, 18-KQLATKAAR-26 and 27-KSAPACGGVKKPHR-40 peptides at various modification levels are shown in panel A. Representative annotated MS/MS spectra are shown in panel B. Underlined lysines correspond to modification sites in panel A.

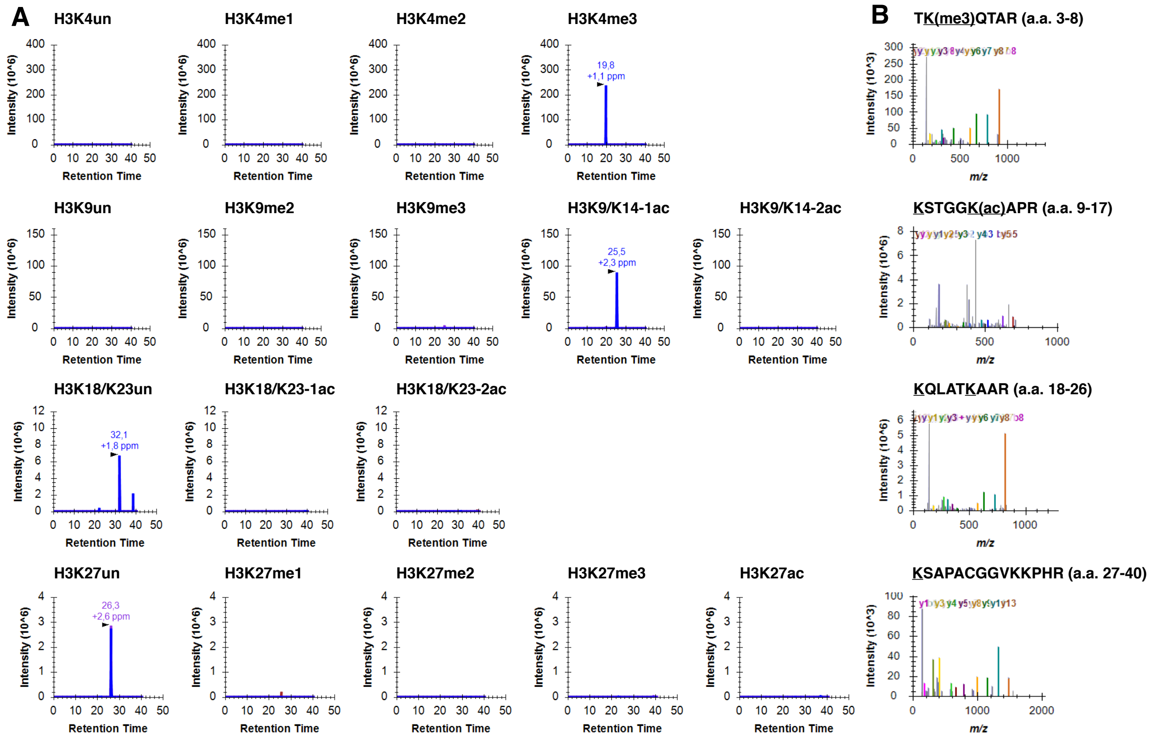

H3K4me3K14ac (histone product 19)

Verification of modification status of ligated H3K4me3K14ac protein. Histone proteins were derivatised with propionic anhydride and phenyl isocynate and analysed using liquid chromatography mass spectrometry (LC-MS) as described in the methods. Extracted ion chromatograms of 3-TKQTAR-8, 9-KSTGGKAPR-17, 18-KQLATKAAR-26 and 27-KSAPACGGVKKPHR-40 peptides at various modification levels are shown in panel A. Representative annotated MS/MS spectra are shown in panel B. Underlined lysines correspond to modification sites in panel A.

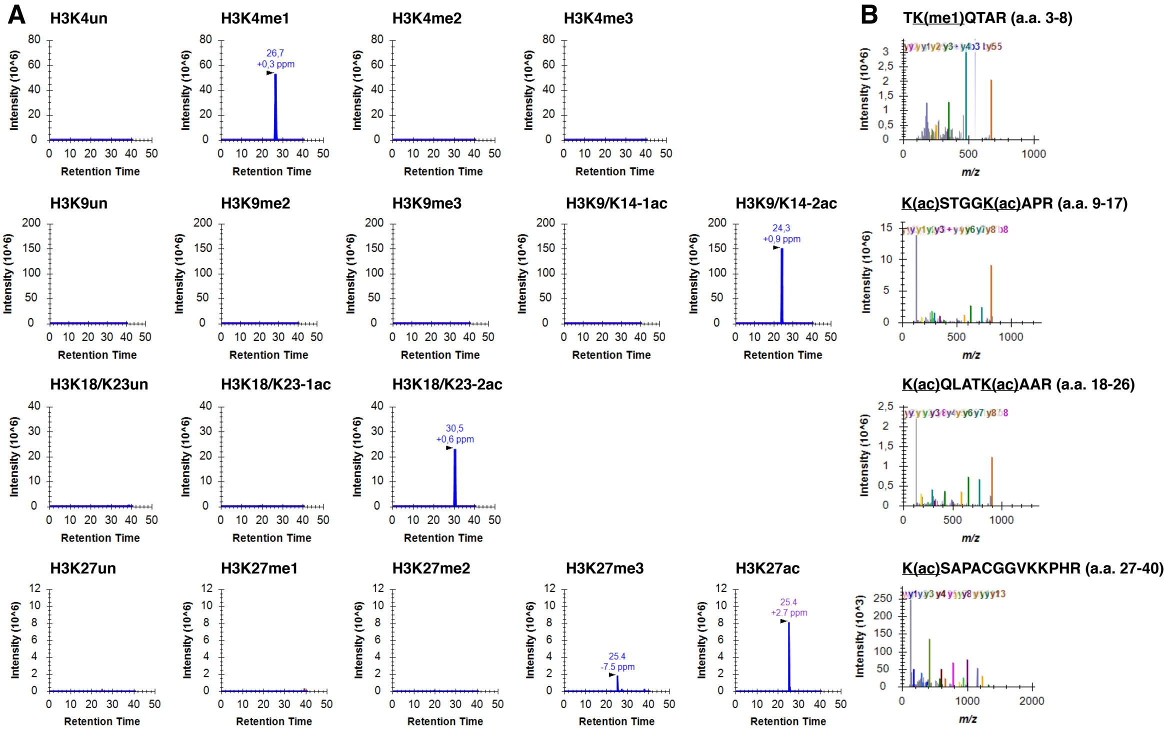

H3K4me1-5ac (H3K4me1K9acK14acK18acK23acK27ac, histone product 20)

Verification of modification status of ligated H3K4me1K9acK14acK18acK23acK27ac protein. Histone proteins were derivatised with propionic anhydride and phenyl isocynate and analysed using liquid chromatography mass spectrometry (LC-MS) as described in the methods. Extracted ion chromatograms of 3-TKQTAR-8, 9-KSTGGKAPR-17, 18-KQLATKAAR-26 and 27-KSAPACGGVKKPHR-40 peptides at various modification levels are shown in panel A. Representative annotated MS/MS spectra are shown in panel B. Underlined lysines correspond to modification sites in panel A.

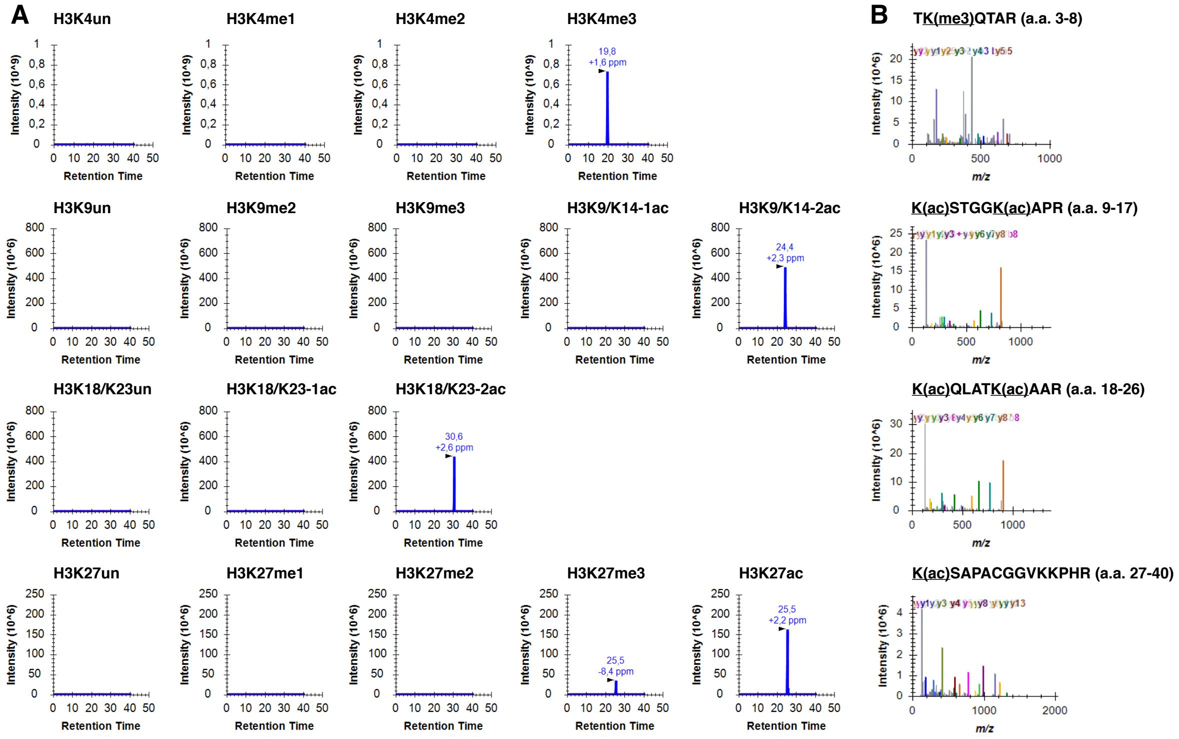

H3K4me3-5ac (H3K4me3K9acK14acK18acK23acK27ac, histone product 21)

Verification of modification status of ligated H3K4me3K9acK14acK18acK23acK27ac protein. Histone proteins were derivatised with propionic anhydride and phenyl isocynate and analysed using liquid chromatography mass spectrometry (LC-MS) as described in the methods. Extracted ion chromatograms of 3-TKQTAR-8, 9-KSTGGKAPR-17, 18-KQLATKAAR-26 and 27-KSAPACGGVKKPHR-40 peptides at various modification levels are shown in panel A. Representative annotated MS/MS spectra are shown in panel B. Underlined lysines correspond to modification sites in panel A.

Histone H4 products

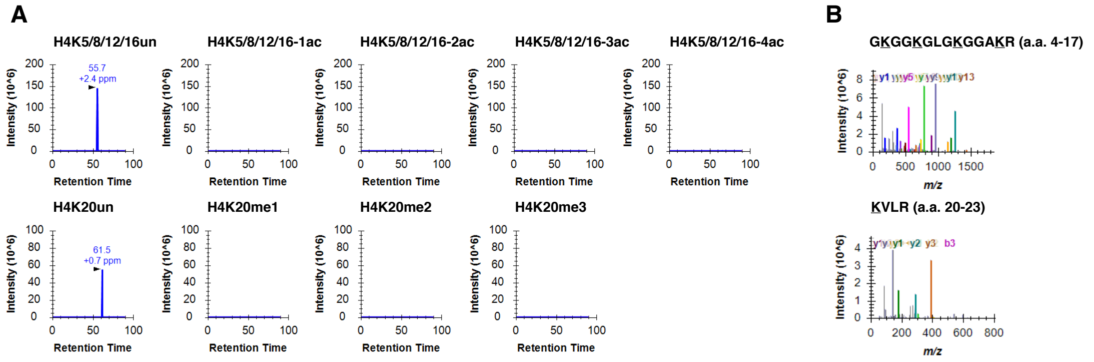

Unmodified Histone H4 (recombinant, histone product 22a)

Verification of modification status of recombinant unmodified histone H4 protein. Histone proteins were derivatised with propionic anhydride and phenyl isocynate and analysed using liquid chromatography mass spectrometry (LC-MS) as described in the methods. Extracted ion chromatograms of 4-GKGGKGLGKGGAKR-17 and 20-KVLR-23 peptides at various modification levels are shown in panel A. Representative annotated MS/MS spectra are shown in panel B. Underlined lysines correspond to modification sites in panel A.

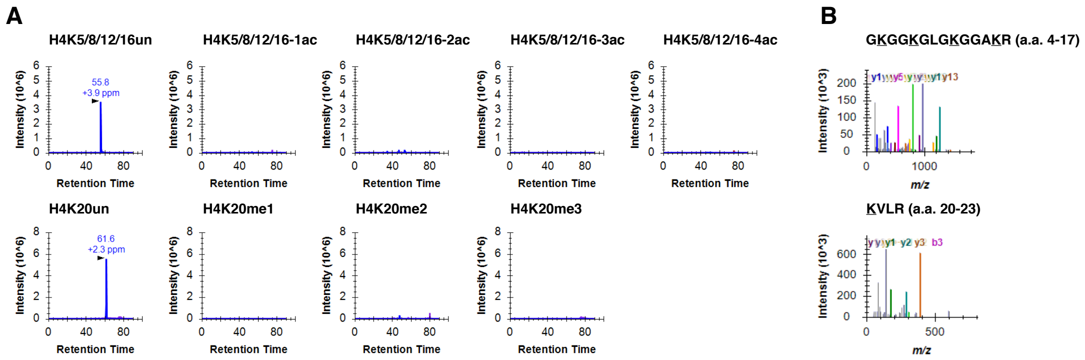

Unmodified Histone H4 (ligated, histone product 22b)

Verification of modification status of ligated unmodified histone H4 protein. Histone proteins were derivatised with propionic anhydride and phenyl isocynate and analysed using liquid chromatography mass spectrometry (LC-MS) as described in the methods. Extracted ion chromatograms of 4-GKGGKGLGKGGAKR-17 and 20-KVLR-23 peptides at various modification levels are shown in panel A. Representative annotated MS/MS spectra are shown in panel B. Underlined lysines correspond to modification sites in panel A.

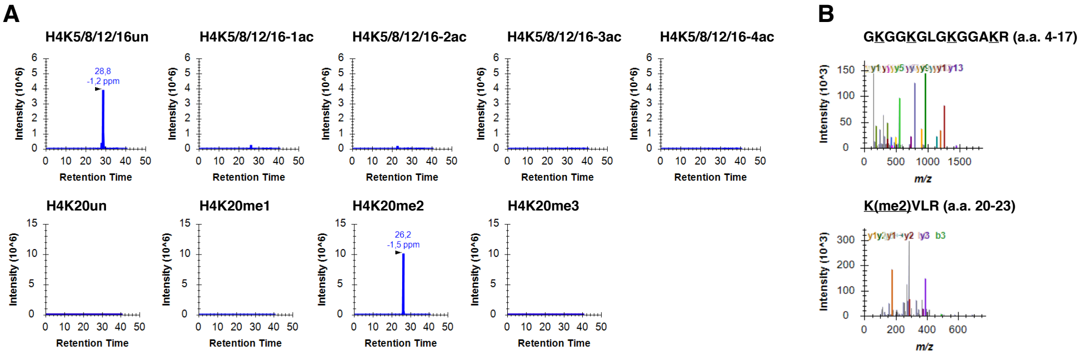

H4K20me2 (histone product 23)

Verification of modification status of ligated H4K20me2 protein. Histone proteins were derivatised with propionic anhydride and phenyl isocynate and analysed using liquid chromatography mass spectrometry (LC-MS) as described in the methods. Extracted ion chromatograms of 4-GKGGKGLGKGGAKR-17 and 20-KVLR-23 peptides at various modification levels are shown in panel A. Representative annotated MS/MS spectra are shown in panel B. Underlined lysines correspond to modification sites in panel A.

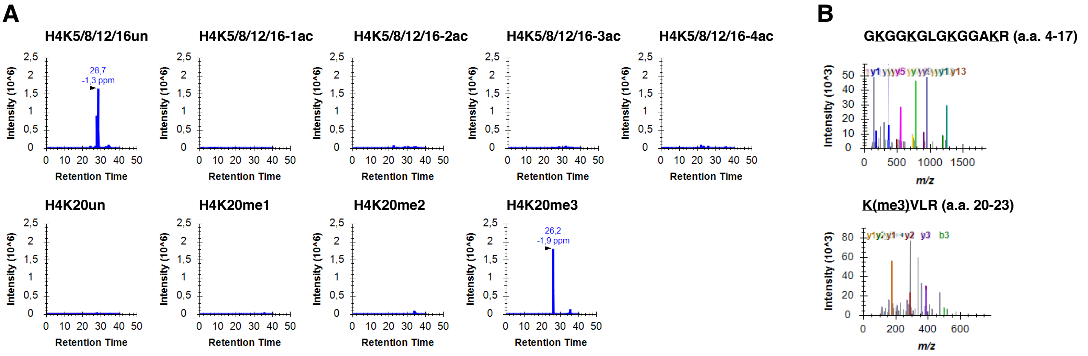

H4K20me3 (histone product 24)

Verification of modification status of ligated H4K20me3 protein. Histone proteins were derivatised with propionic anhydride and phenyl isocynate and analysed using liquid chromatography mass spectrometry (LC-MS) as described in the methods. Extracted ion chromatograms of 4-GKGGKGLGKGGAKR-17 and 20-KVLR-23 peptides at various modification levels are shown in panel A. Representative annotated MS/MS spectra are shown in panel B. Underlined lysines correspond to modification sites in panel A.

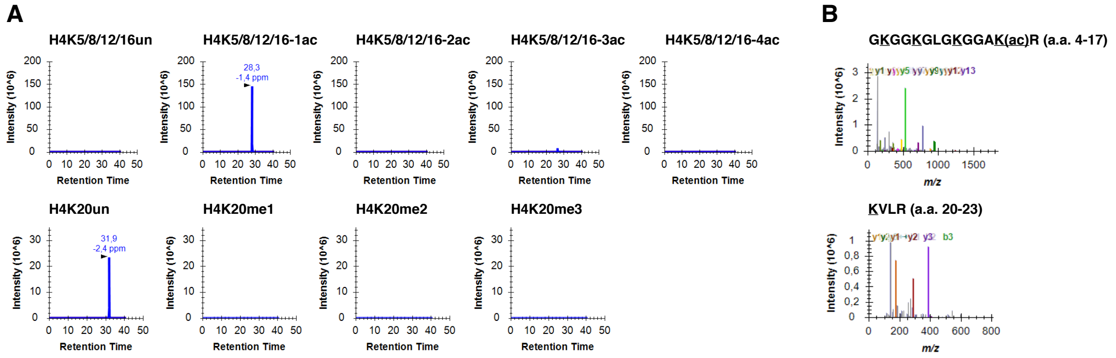

H4K16ac (histone product 25)

Verification of modification status of ligated H4K16ac protein. Histone proteins were derivatised with propionic anhydride and phenyl isocynate and analysed using liquid chromatography mass spectrometry (LC-MS) as described in the methods. Extracted ion chromatograms of 4-GKGGKGLGKGGAKR-17 and 20-KVLR-23 peptides at various modification levels are shown in panel A. Representative annotated MS/MS spectra are shown in panel B. Underlined lysines correspond to modification sites in panel A.

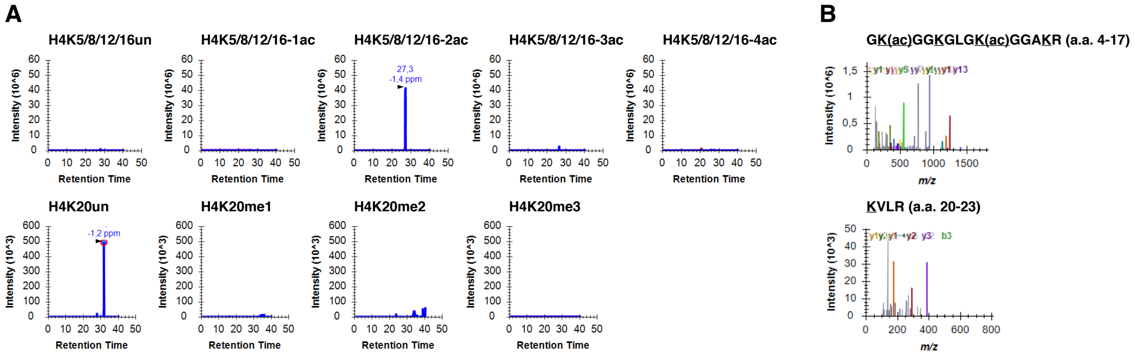

H4K5acK12ac (histone product 26)

Verification of modification status of ligated H4K5acK12ac protein. Histone proteins were derivatised with propionic anhydride and phenyl isocynate and analysed using liquid chromatography mass spectrometry (LC-MS) as described in the methods. Extracted ion chromatograms of 4-GKGGKGLGKGGAKR-17 and 20-KVLR-23 peptides at various modification levels are shown in panel A. Representative annotated MS/MS spectra are shown in panel B. Underlined lysines correspond to modification sites in panel A.

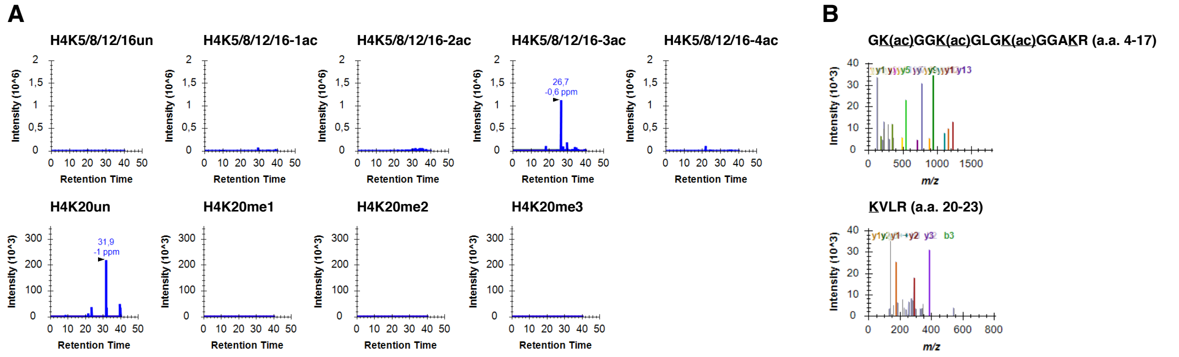

H4K5acK8acK12ac (histone product 27)

Verification of modification status of ligated H4K5acK8acK12ac protein. Histone proteins were derivatised with propionic anhydride and phenyl isocynate and analysed using liquid chromatography mass spectrometry (LC-MS) as described in the methods. Extracted ion chromatograms of 4-GKGGKGLGKGGAKR-17 and 20-KVLR-23 peptides at various modification levels are shown in panel A. Representative annotated MS/MS spectra are shown in panel B. Underlined lysines correspond to modification sites in panel A.

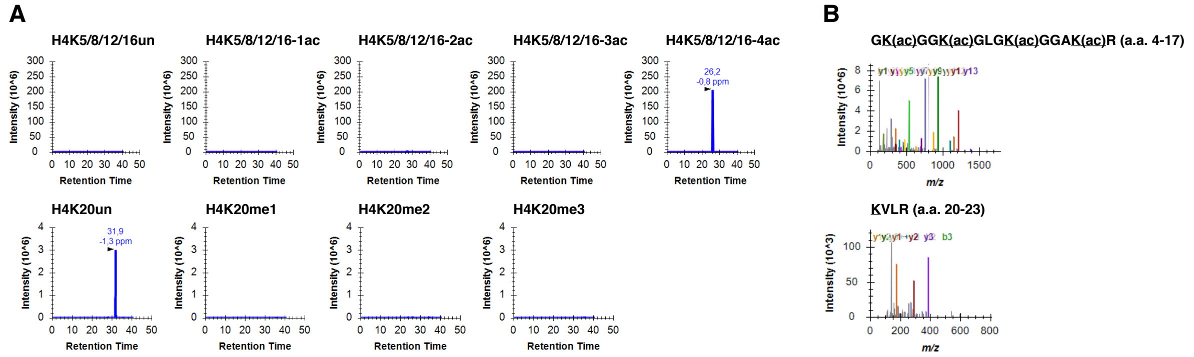

H4K5acK8acK12acK16ac (histone product 28)

Verification of modification status of ligated H4K5acK8acK12acK16ac protein. Histone proteins were derivatised with propionic anhydride and phenyl isocynate and analysed using liquid chromatography mass spectrometry (LC-MS) as described in the methods. Extracted ion chromatograms of 4-GKGGKGLGKGGAKR-17 and 20-KVLR-23 peptides at various modification levels are shown in panel A. Representative annotated MS/MS spectra are shown in panel B. Underlined lysines correspond to modification sites in panel A.

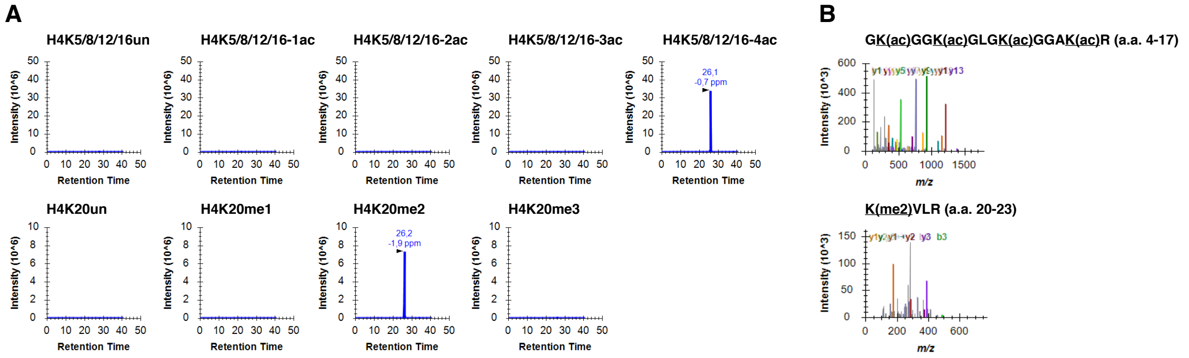

H4K5acK8acK12acK16acK20me2 (histone product 29)

Verification of modification status of ligated H4K5acK8acK12acK16acK20me2 protein. Histone proteins were derivatised with propionic anhydride and phenyl isocynate and analysed using liquid chromatography mass spectrometry (LC-MS) as described in the methods. Extracted ion chromatograms of 4-GKGGKGLGKGGAKR-17 and 20-KVLR-23 peptides at various modification levels are shown in panel A. Representative annotated MS/MS spectra are shown in panel B. Underlined lysines correspond to modification sites in panel A.

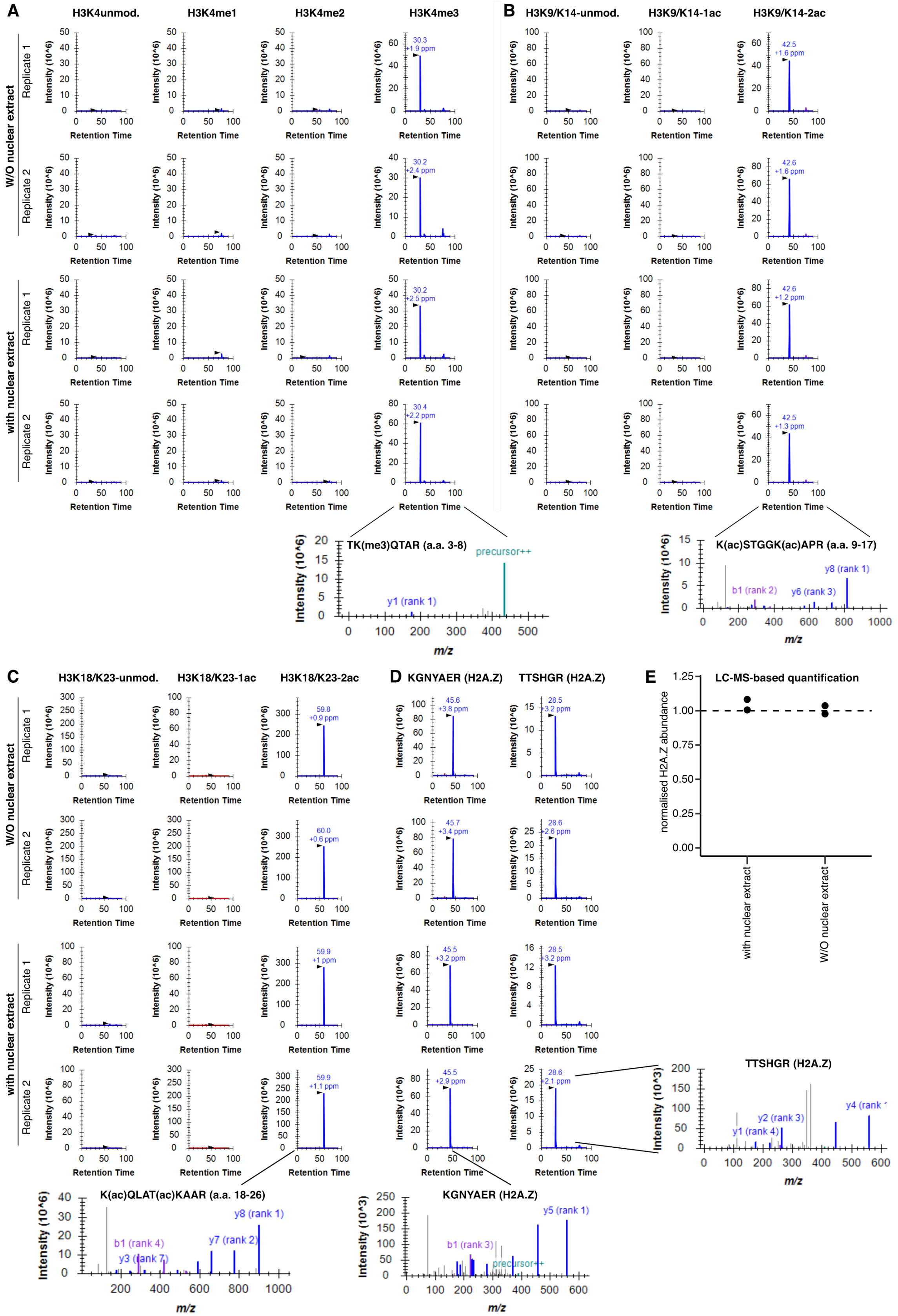

Evaluation of the stability of nucleosomal modifications during the incubation with HeLa nuclear extract (part 1)

(legend on the next page)

(continued from previous page)

(A-C) Verification of the stability of post-translational modifications present on histone H3 during di-nucleosome affinity purification experiments with HeLa S3 nuclear extract. Di-nucleosomes decorated with H3K4me3K9acK14acK18acK23acK27ac (H3acK4me3), H4K5acK8acK12acK16acK20me2 (H4acK20me2), and incorporating the histone variant H2A.Z were used in a label-free nucleosome pull-down experiment with HeLa S3 nuclear extract or buffer only (W/O nuclear extract control). Each pull-down was performed in n=2 independent experimental replicates. Histone proteins were separated by SDS PAGE, derivatised with propionic anhydride, digested with trypsin, treated with phenyl isocyanate, and analysed using label-free liquid chromatography-mass spectrometry (LC-MS) as described in the methods. Extracted ion chromatograms of histone H3 peptides at various modification levels and representative annotated MS/MS spectra of the modified peptide species are shown in panels (A), (B), and (C). Note that the modification status does not change after the pull-down with HeLa S3 nuclear extract. (D-E) Verification of the stability of histone variant H2A.Z in the nucleosome pull-down experiments described in panels (A-C) above. Extracted ion chromatograms of peptides uniquely present in the histone variant H2A.Z as well as their annotated MS/MS spectra are shown in (D). The dot plot in (E) shows the normalized relative abundance of histone variant H2A.Z calculated as the mean MS1 intensity of H2A.Z-specific peptides divided by the mean intensity of H3 and H4 peptides and normalized to the mean relative abundance of H2A.Z in the “W/O nuclear extract” control purifications. Note that the abundance of H2A.Z does not change after the pull-down with HeLa S3 nuclear extract.

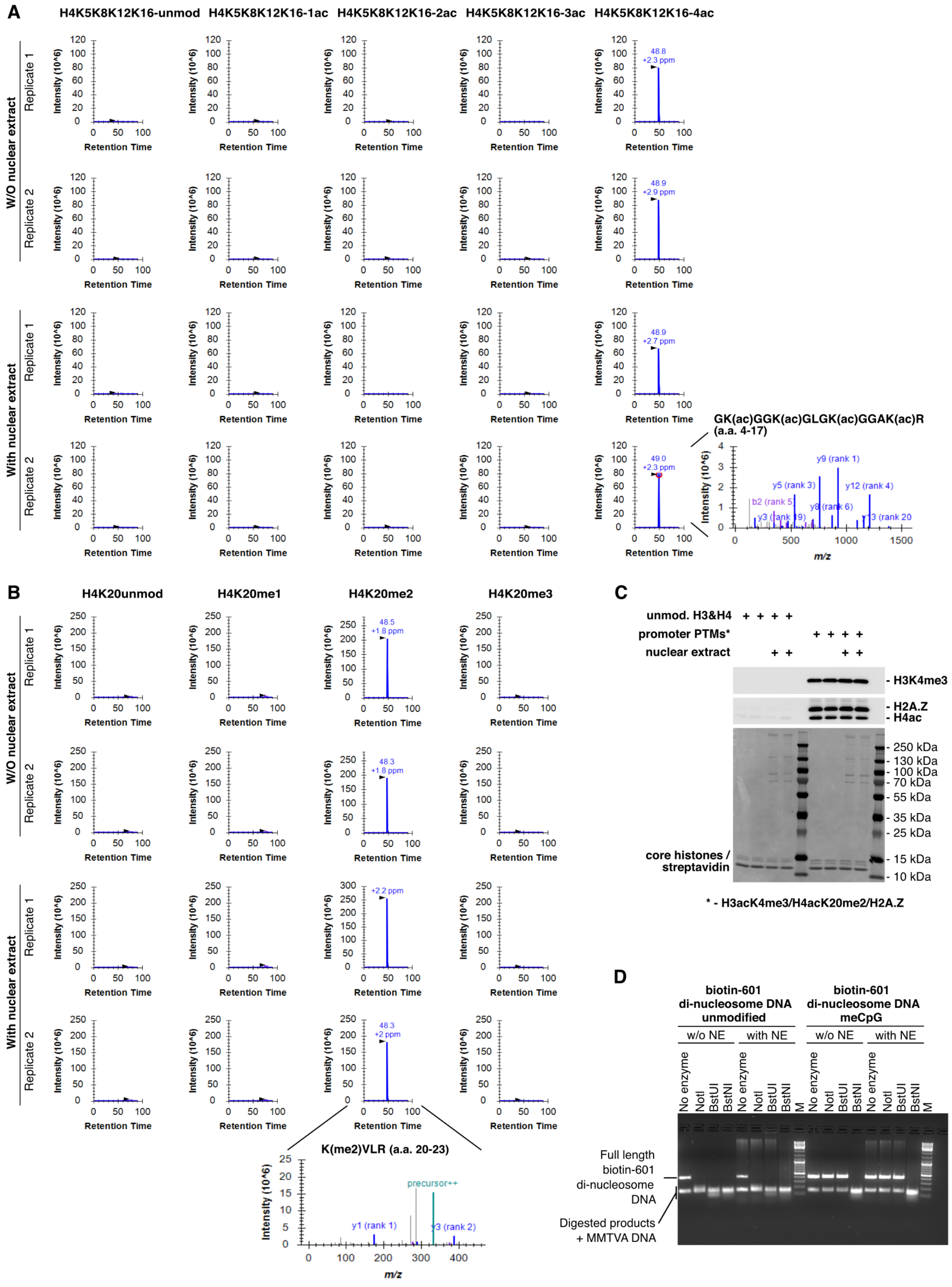

Evaluation of the stability of nucleosomal modifications during the incubation with HeLa nuclear extract (part 2)

(legend on the next page)

(continued from previous page)

(A-B) Verification of the stability of post-translational modifications present on histone H4 during di-nucleosome pull-downs with HeLa S3 nuclear extract. Di-nucleosomes decorated with H3K4me3K9acK14acK18acK23acK27ac (H3acK4me3), H4K5acK8acK12acK16acK20me2 (H4acK20me2), and incorporating the histone variant H2A.Z were used in a label-free nucleosome pull-down experiment with HeLa S3 nuclear extract or buffer only (W/O nuclear extract control). Each pull-down was performed in n=2 independent experimental replicates. Histone proteins were separated by SDS PAGE, derivatised with propionic anhydride, digested with trypsin, treated with phenyl isocyanate, and analysed using label-free liquid chromatography-mass spectrometry (LC-MS) as described in the methods. Extracted ion chromatograms of histone H4 peptides at various modification levels and representative annotated MS/MS spectra are shown in panels (A) and (B). Note that the modification status does not change after the pull-down with HeLa S3 nuclear extract. (C) Unmodified di-nucleosomes and di-nucleosomes decorated with H3acK4me3/H4acK20me2/H2A.Z (marked as ‘Promoter PTMs’) were used in nucleosome pull-down experiments with HeLa S3 nuclear extract or buffer only as above and the histone modification status after the pull-down was examined by western blot. Note that the levels of H3K4me3, H4ac (H4K5acK8acK12acK16ac), and the histone variant H2A.Z do not change after the pull-down with HeLa S3 nuclear extract. Equal loading was confirmed by ponceau staining. Molecular weights of marker proteins (PageRuler Plus - Thermo Scientific) are indicated on the right. Each pull-down was performed in n=2 independent experimental replicates, both replicates are shown in the panel. (D) Verification of the stability of DNA methylation during di-nucleosome pull-down experiments with HeLa nuclear extract. Di-nucleosomes containing unmodified recombinant wild type histones and either unmethylated or CpG-methylated biotin 601 di-nucleosome DNA (see section *Unmethylated and CpG-methylated biotin-601 di-nucleosome DNA*) were assembled in the presence of MMTVA competitor DNA and incubated with a HeLa S3 nuclear extract (with NE) or buffer only (w/o NE control). After the incubation the DNA was re-purified from the reaction via a PCR clean-up column and the methylation status of the biotin 601 di-nucleosome DNA was assessed by digestion with the methylation-sensitive restriction enzymes NotI and BstUI. The BstNI enzyme, that is not CpG methylation-sensitive, was used as a control. Note that unmethylated DNA does not become methylated and methylated DNA is not demethylated during the incubation with nuclear extract. This control was performed once. Markers (1kb Marker – Invitrogen) are labelled “M”.

Key Resources Table

REAGENT or RESOURCE	SOURCE	IDENTIFIER
**Antibodies**		
Anti-V5 tag	eBioscience	TCM5 #14-6796-82
Anti-V5 tag	Abcam	ab15828
Anti-INO80	Abcam	ab118787
Anti-INO80B	Santa Cruz	(E-3): sc-390009
Anti-ACTR5	GeneTex	GTX80453
Anti-TBRG1	Santa Cruz	(D-9): sc-515620
Anti-H3	Active motif	39163
Anti-H3K4me1	Abcam	ab8895
Anti-H3K4me3	Millipore	17-614
Anti-H4	Abcam	ab31830
Anti-H4ac (pan-acetyl)	Active motif	39967
Anti-CBX4	Cell Signaling Technology	E6L7X #30559
Anti-CBX8	Santa Cruz	(C-3): sc-374332
Anti-H2B	Abcam	ab1790
Anti-H2A.Z	Abcam	ab4174
Alexa Fluor® 488 Anti-Mouse IgG	Jackson ImmunoResearch Laboratories	715-545-150
**Bacterial strains**		
E. coli BL21-CodonPlus (DE3)-RIL	Agilent	Cat#230245
E. coli XL-10 Gold	Agilent	Cat#200317
**Chemicals**		
4-Mercaptophenylacetic acid (MPAA)	Sigma	Cat#653152
Tris(2-carboxyethyl)phosphine hydrochloride (TCEP)	Sigma	Cat#C4706
DL-Dithiothreitol (DTT)	Sigma	Cat#D9779
Acetonitrile (LC/MS Grade)	Fisher Scientific	Cat#A955-1
Water (LC/MS Grade)	Fisher Scientific	Cat#W6212

Trifluoroacetic acid (TFA)	Sigma	Cat#91707
Sodium butyrate	Sigma	Cat#B5887
Trichostatin A (TSA)	Sigma	Cat#T1952
Iodoacetamide	Sigma	Cat#I1149
Urea	Sigma	Cat#U1250
Triethylammonium bicarbonate buffer	Sigma	Cat#T7408
Propionic anhydride	Sigma	Cat#240311
Phenyl isocyanate	Sigma	Cat#185353
Trypsin	Promega	Cat#V5113
C18 peptide desalting stagetips	Glygen	Cat#TT2C18
Pierce C-18 spin columns	Thermo Scientific	Cat#89870
SILAC-labelled heavy L-Arginine-$^{13}C_6$, $^{15}N_4$ · HCl	Sigma/Isotec	Cat#608033
SILAC-labelled heavy L-Lysine-$^{13}C_6$, $^{15}N_2$ · HCl	Sigma/Isotec	Cat#608041
Unlabelled L-Arginine-$^{12}C_6$, $^{14}N_4$ · HCl	Sigma	Cat#A6969
Unlabelled L-Lysine-$^{12}C_6$, $^{14}N_2$ · HCl	Sigma	Cat#L8662
RPMI 1640 Medium for SILAC (Arg/Lys-free)	ThermoFisher Scientific	Cat#88365
Dialysed FBS for SILAC	ThermoFisher Scientific/Gibco	Cat#26400044
Biotin-11-dUTP	Yorkshire Bioscience Ltd	Cat#P1612
Streptavidin Sepharose high performance	Cytiva	Cat#17-5113-01
Dynabeads® Protein G	Invitrogen	10004D
Dynabeads® Protein A	Invitrogen	10002D
GFP-Trap® Agarose beads	ChromoTek	gta-20
Lipofectamine CRISPRMAX Cas9 Transfection Reagent	Invitrogen	Cat#CMAX00008
TrueCut Cas9 Protein v2	Invitrogen	Cat#A36498
Micrococcal nuclease	ThermoFisher Scientific	Cat#ENO181
HRV-3C Protease, Biotin tagged	Sigma-Aldrich	Cat#SAE0110
EGS (Ethylene Glycol bis (Succinimidyl Succinate))	ThermoFisher Scientific	Cat#21565

DSG (disuccinimidyl glutarate)	ThermoFisher Scientific	Cat#20593
**Peptides**		
H3unmod (aa 1-31, C-terminal benzyl thioester) peptide: ARTKQTARKSTGGKAPRKQLATKAARKSAPA-S-Bzl	Cambridge Peptides	Peptide code: 15555
H3K9me2 (aa 1-31, C-terminal benzyl thioester) peptide: ARTKQTARK(Me2)STGGKAPRKQLATKAARKSAPA-S-Bzl	Cambridge Peptides	Peptide code: 15556
H3K9me3 (aa 1-31, C-terminal benzyl thioester) peptide: ARTKQTARK(Me3)STGGKAPRKQLATKAARKSAPA-S-Bzl	Cambridge Peptides	Peptide code: 15557
H3K9me3K27me3 (aa 1-31, C-terminal benzyl thioester) peptide: ARTKQTARK(Me3)STGGKAPRKQLATKAARK(Me3)SAPA-S-Bzl	Almac Sciences	Peptide code: 1201P03
H3K27me2 (aa 1-31, C-terminal benzyl thioester) peptide: ARTKQTARKSTGGKAPRKQLATKAARK(Me2)SAPA-S-Bzl	Cambridge Peptides	Peptide code: 24148
H3K27me3 (aa 1-31, C-terminal benzyl thioester) peptide: ARTKQTARKSTGGKAPRKQLATKAARK(Me3)SAPA-S-Bzl	Almac Sciences	Peptide code: 1201P01
H3K4me1K27me3 (aa 1-31, C-terminal benzyl thioester) peptide: ARTK(Me)QTARKSTGGKAPRKQLATKAARK(Me3)SAPA-S-Bzl	Cambridge Peptides	Peptide code: 15563

H3K4me1K18acK23acK27me3 (aa 1-31, C-terminal thioester) peptide: ARTK(Me)QTARKSTGGKAPRK(Ac)QLATK(Ac)AARK(Me3)SAPA-S-Bzl	Cambridge Peptides	Peptide code: 15565
H3K4me1K9acK14acK27me3 (aa 1-31, C-terminal benzyl thioester) peptide: ARTK(Me)QTARK(Ac)STGGK(Ac)APRKQLATKAARK(Me3)SAPA-S-Bzl	Cambridge Peptides	Peptide code: 15564
H3K4me1 (aa 1-31, C-terminal ethyl 3-mercaptopropionate thioester) peptide: ARTK(Me)QTARKSTGGKAPRKQLATKAARKSAPA-S-(CH2)2CO2Et	Almac Sciences	Peptide code: 941P03
H3K4me1K27ac (aa 1-31, C-terminal benzyl thioester) peptide: ARTK(Me)QTARKSTGGKAPRKQLATKAARK(Ac)SAPA-S-Bzl	Cambridge Peptides	Peptide code: 34341
H3K27ac (aa 1-31, C-terminal benzyl thioester) peptide: ARTKQTARKSTGGKAPRKQLATKAARK(Ac)SAPA-S-Bzl	Cambridge Peptides	Peptide code: 34342
H3K4me1K9acK14ac (aa 1-31, C-terminal benzyl thioester) peptide: ARTK(Me)QTARK(Ac)STGGK(Ac)APRKQLATKAARKSAPA-S-Bzl	Cambridge Peptides	Peptide code: 15561
H3K9acK14ac (aa 1-31, C-terminal benzyl thioester) peptide: ARTKQTARK(Ac)STGGK(Ac)APRKQLATKAARKSAPA-S-Bzl	Cambridge Peptides	Peptide code: 15560
H3K4me3K9acK14ac (aa 1-31, C-terminal benzyl thioester) peptide: ARTK(Me3)QTARK(Ac)STGGK(Ac)APRKQLATKAARKSAPA-S-Bzl	Cambridge Peptides	Peptide code: 24153

H3K4me3K9acK18ac (aa 1-31, C-terminal benzyl thioester) peptide: ARTK(Me3)QTARK(Ac)STGGKAPRK(Ac)QLATKAARKSAPA-S-Bzl	Cambridge Peptides	Peptide code: 24154
H3K4me3K9ac (aa 1-31, C-terminal benzyl thioester) peptide: ARTK(Me3)QTARK(Ac)STGGKAPRKQLATKAARKSAPA-S-Bzl	Cambridge Peptides	Peptide code: 24156
H3K4me3 (aa 1-31, C-terminal benzyl thioester) peptide: ARTK(Me3)QTARKSTGGKAPRKQLATKAARKSAPA-S-Bzl	Cambridge Peptides	Peptide code: 34339
H3K4me3K14ac (aa 1-31, C-terminal benzyl thioester) peptide: ARTK(Me3)QTARKSTGGK(Ac)APRKQLATKAARKSAPA-S-Bzl	Cambridge Peptides	Peptide code: 24155
H3K4me1-5ac (aa 1-31, C-terminal benzyl thioester) peptide: ARTK(Me)QTARK(Ac)STGGK(Ac)APRK(Ac)QLATK(Ac)AARK(Ac)SAPA-S-Bzl	Cambridge Peptides	Peptide code: 15562
H3K4me3-5ac (aa 1-31, C-terminal benzyl thioester) peptide: ARTK(Me3)QTARK(Ac)STGGK(Ac)APRK(Ac)QLATK(Ac)AARK(Ac)SAPA-S-Bzl	Cambridge Peptides	Peptide code: 15566
H4unmod (aa 1-28, N-terminal acetylation, C-terminal benzyl thioester) peptide: Ac-SGRGKGGKGLGKGGAKRHRKVLRDNIQG-S-Bzl	Cambridge Peptides	Peptide codes: 15568 & 34343
H4K20me2 (aa 1-28, N-terminal acetylation, C-terminal benzyl thioester) peptide: Ac-SGRGKGGKGLGKGGAKRHRK(Me2)VLRDNIQG-S-Bzl	Almac Sciences	Peptide code: 1201P05

H4K20me3 (aa 1-28, N-terminal acetylation, C-terminal benzyl thioester) peptide: Ac-SGRGKGGKGLGKGGAKRHRK(Me3)VLRDNIQG-S-Bzl	Almac Sciences	Peptide code: 1201P06
H4K16ac (aa 1-28, N-terminal acetylation, C-terminal benzyl thioester) peptide: Ac-SGRGKGGKGLGKGGAK(Ac)RHRKVLRDNIQG-S-Bzl	Cambridge Peptides	Peptide code: 15569
H4K5acK12ac (aa 1-28, N-terminal acetylation, C-terminal benzyl thioester) peptide: Ac-SGRGK(Ac)GGKGLGK(Ac)GGAKRHRKVLRDNIQG-S-Bzl	Cambridge Peptides	Peptide code: 34344
H4K5acK8acK12ac (aa 1-28, N-terminal acetylation, C-terminal benzyl thioester) peptide: Ac-SGRGK(Ac)GGK(Ac)GLGK(Ac)GGAKRHRKVLRDNIQG-S-Bzl	Cambridge Peptides	Peptide code: 34345
H4K5acK8acK12acK16ac (aa 1-28, N-terminal acetylation, C-terminal thioester) peptide: Ac-SGRGK(Ac)GGK(Ac)GLGK(Ac)GGAK(Ac)RHRKVLRDNIQG-S-Bzl	Cambridge Peptides	Peptide code: 15570
H4K5acK8acK12acK16acK20me2 (aa 1-28, N-terminal acetylation, C-terminal thioester) peptide: Ac-SGRGK(Ac)GGK(Ac)GLGK(Ac)GGAK(Ac)RHRK(Me2)VLRDNIQG-S-Bzl	Cambridge Peptides	Peptide code: 24157
**Deposited Data**		
SILAC di-nucleosome purification experiments	This paper	PRIDE: PXD018966
H3K4me1 and H3K4me3 ChIP-MS (histone PTMs)	This paper	PRIDE: PXD042224
H3K4me1 and H3K4me3 ChIP-MS (proteins)	This paper	PRIDE: PXD042826
Label-free di-nucleosome purification experiments with 200bp SV40 promoter linker	This paper	PRIDE: PXD041835

Label-free di-nucleosome purification experiments with 200bp SV40 enhancer linker	This paper	PRIDE: PXD041443
Label-free di-nucleosome purification experiments with short linkers and heterochromatic PTMs	This paper	PRIDE: PXD042368
INO80B-V5 IP-MS data	This paper	PRIDE: PXD020712
ACTR5 ChIP-MS	This paper	PRIDE: PXD042210
Effect of native chemical ligation on protein binding	This paper	PRIDE: PXD042390
MS Histone PTM analysis of ligated and recombinant H3 and H4 protein products	This paper	PRIDE: PXD020773
Stability of nucleosomal modifications during pull-down affinity purification in nuclear extract	This paper	PRIDE: PXD042823
**Cell Lines**		
HeLa S3 (RRID: CVCL_0058)	ATCC	Cat# CCL-2.2
MCF-7 (RRID: CVCL_0031)	ATCC	Cat# HTB-22
MCF-7 with endogenous INO80B-V5 fusion	This paper	N/A
IMR90 human fibroblast (RRID: CVCL_0347)	ATCC	Cat# CCL-186
HeLa Kyoto BAC cell line expressing the C terminally LAP (localization and affinity purification) –tagged INO80 subunit ACTR5	Ref. [13]	Cell line ID: MCP_ky_0007413 (Ref. [13])
**Oligonucleotides**		
Forward annealing oligo for 55bp linker: CAT GGC GGC CGC GTA GTA CTG AAT CAT ACT AAG GCA TCT GTA ATC GGA TCT TG	Metabion	N/A
Reverse annealing oligo for 55bp linker: CTA GCA AGA TCC GAT TAC AGA TGC CTT AGT ATG ATT CAG TAC TAC GCG GCC GC	Metabion	N/A
Forward annealing oligo for 50bp control linker: CAT GGC GGC CGC GTA GTA CTG AAT CAT AGC ATC TGT AAT CGG ATC TTG	Metabion	N/A

Reverse annealing oligo for 50bp control linker:<br>CTA GCA AGA TCC GAT TAC AGA TGC TAT GAT TCA GTA CTA CGC GGC CGC	Metabion	N/A
Forward annealing oligo for 45bp linker:<br>CAT GGC GGC CGC GTA GTA CTG AAT CAT CTG TAA TCG GAT CTT G	Metabion	N/A
Reverse annealing oligo for 45bp linker:<br>CTA GCA AGA TCC GAT TAC AGA TGA TTC AGT ACT ACG CGG CCG C	Metabion	N/A
Forward annealing oligo for 40bp linker:<br>CAT GGC GGC CGC GTA GTA CTG AAT GTA ATC GGA TCT TG	Metabion	N/A
Reverse annealing oligo for 40bp linker:<br>CTA GCA AGA TCC GAT TAC ATT CAG TAC TAC GCG GCC GC	Metabion	N/A
Forward annealing oligo for 35bp linker:<br>CAT GGC GGC CGC GTA GTA CTG AAT CGG ATC TTG	Metabion	N/A
Reverse annealing oligo for 35bp linker:<br>CTA GCA AGA TCC GAT TCA GTA CTA CGC GGC CGC	Metabion	N/A
Forward PCR primer for the amplification of the SV40 enhancer from pGL3-Control:<br>ATT CTC CAT GGC TTC TGA GGC GGA AAG AAC CAG CTG TGG AAT GTG TGT CAG TTA GGG	Metabion	N/A
Reverse PCR primer for the amplification of the SV40 enhancer from pGL3-Control:<br>CCT GTG CTA GCA CTA TGG TTG CTG ACT AAT TGA G	Metabion	N/A
Forward PCR primer for the amplification of the SV40 promoter from pGL3-Control:<br>TCC TGT GCT AGC CTC AAT TAG TCA GCA ACC ATA GTC	Metabion	N/A

Reverse PCR primer for the amplification of the SV40 promoter from pGL3-Control:<br>GAT TCT CCA TGG TTT GCA AAA GCC TAG GCC TCC A	Metabion	N/A
200bp scrambled linker oligo (NcoI and NdeI restriction sites are in *italic*; 5' bGHR and 3' pCIfor primer binding sites are underlined):<br>TAG AAG GCA CAG TCG AGG ATT CT*C CAT GG*N NNN NNN NNN NNN NNN NNN NNN NNN NNN NNN NNN NNN NNN NNN NNN NNN NNN NNN NNN NNN NNN NNN NNN NNN NNN NNN NNN NNN NNN NNN NNN NNN NNN NNN NNN NNN NNN NNN NNN NNN NNN NNN NNN NNN NNN NNN NNN NNN NNN NNN NNN NNN NNN NNN NNN NNN NNN NNN NNN NNN NNN NNN NNN NNN NNN NNN NNN NNN NN*G CTA GC*A CTG TAA TTG AAC TGG GAG TGG	Biolegio	N/A
pCIfor primer for primer extension:<br>CCA CTC CCA GTT CAA TTA CAG	Sigma	N/A
INO80B V5 ssDNA oligo for HDR:<br>GGG GGG GCC CGA GGG TCC TGG ATC CCC CCT TTT GGC TAC GGA AAA CCT GTA TTT TCA GAG CGG TAA GCC TAT CCC TAA CCC TCT CCT CGG TCT CGA TTC TAC GTA AGG CAC TTA ACC CGC ACT CTG CGC CCC GTC CCA TGC CCG CT	IDT	N/A
INO80B crRNA oligo<br>/AltR1/rGrC rUrArC rGrUrA rArGrG rCrCrC rUrUrA rArCrC rGrUrU rUrUrA rGrArG rCrUrA rUrGrC rU/AltR2/	IDT	N/A
Alt-R® CRISPR-Cas9 tracrRNA	IDT	Cat#1072532
**Recombinant DNA**		
Human H3.1 Δ1-31 T32C, cloned into pET28a(+)	Ref. [14]	pTB070
Human H4 Δ1-28 T29C, cloned into pET24b(+)	Ref. [15]	pTB089

Human H2A, cloned into pET21b(+)	Ref. [14]	pTB037
Human H2B, cloned into pET21b(+)	Ref. [14]	pTB008
Human H3.1, cloned into pET21b(+)	Ref. [14]	pTB013
Human H4, cloned into pET21b(+)	Ref. [14]	pTB009
Human H2A.Z, cloned into pET24a(+)	This paper	pTB636
pUC19-16x601	Ref. [14]	pTB050
pUC19-8xdi601	Ref. [15]	pTB401
pUC19-di601_NcoI/NheI_5xGal4	This paper	pTB891
pUC19-di601_NcoI/NheI_200bp_scrambled_linker	This paper	pTB977 (original library)
pUC19-di601_NcoI/NheI_200bp_scrambled_linker	This paper	pTB978 (amplified library)
pUC19-8xdi601_NcoI/NheI_SV40_promoter	This paper	pTB948
pUC19-8xdi601_NcoI/NheI_SV40_enhancer	This paper	pTB952
pUC19-8xdi601_NcoI/NheI_35bp_linker	This paper	pTB960
pUC19-8xdi601_NcoI/NheI_40bp_linker	This paper	pTB964
pUC19-8xdi601_NcoI/NheI_45bp_linker	This paper	pTB968
pUC19-8xdi601_NcoI/NheI_50bp_linker	This paper	pTB972
pUC19-8xdi601_NcoI/NheI_55bp_linker	This paper	pTB976
pUC19-4xtetra601	Ref. [16]	pTB685
pUC19-MMTVA	Thomas Schalch	pTB027
pGL3-Control	Promega	Cat#E1741
**Software**		
Bedtools (v2.30.0)	Ref. [17]	https://github.com/arq5x/bedtools2; RRID:SCR_006646
Bnstruct (R package, v1.0.8)	Ref. [18]	https://cran.r-project.org/web/packages/bnstruct/index.html
CAMERA	Ref. [19]	See *limma*

Cytoscape (v3.7.1)	Ref. [20]	https://cytoscape.org/; RRID:SCR_003032
CLR	Ref. [21]	See *minet*
Gephi (v0.9.2)	Ref. [22]	https://gephi.org/; RRID:SCR_004293
Image Lab Touch Software (v3.3.0.07)	BioRad	N/A
limma (R package, v3.42.2, v3.50.1)	Ref. [23]	https://bioconductor.org/packages/release/bioc/html/limma.html; RRID:SCR_010943
Mascot (v2.6.2)	Ref. [24]	http://www.matrixscience.com/mascot_support_v2_6.html; RRID:SCR_014322
Matplotlib (Python package, v3.0.3, v3.4.3, v3.5.1)	Ref. [25]	https://matplotlib.org/; RRID:SCR_008624
MaxQuant (v1.5.2.8)	Ref. [26]	https://www.maxquant.org/; RRID:SCR_014485
minet (R package, v3.44.1)	Ref. [27]	https://www.bioconductor.org/packages/release/bioc/html/minet.html

NetworkX (Python package, v2.3)	Ref. [28]	https://networkx.github.io/; RRID:SCR_016864
Pingouin (Python package, v0.5.1)	Ref. [29]	https://pingouin-stats.org/; RRID:SCR_022261
Precrec (R package, v0.11)	Ref. [30]	https://cran.r-project.org/web/packages/precrec/index.html; RRID:SCR_018659
Progenesis QI (v4.1)		http://www.nonlinear.com/progenesis/qi-for-proteomics/; RRID:SCR_018923
Proteome Discoverer (v2.5)	ThermoFisher Scientific	N/A
Protoclust (R package, v1.6.3)	Ref. [31]	https://cran.r-project.org/web/packages/protoclust/index.html
python-louvain (Python package, v0.13)		https://github.com/taynaud/python-louvain
Scikit-learn (Python package, v0.21.1)	Ref. [32]	https://scikit-learn.org/; RRID:SCR_002577

Scipy (Python package, v1.3.0, v1.7.1)	Ref. [33]	https://www.scipy.org/; RRID:SCR_008058
Skyline (v20.1.0.31)	Ref. [34]	https://skyline.ms/project/home/software/Skyline/begin.view; RRID:SCR_014080
Statsmodels (Python package, v0.9.0, v0.12.2, v0.13.2)	Ref. [35]	https://www.statsmodels.org/; RRID:SCR_016074
Xcalibur (LC-MS data acquisition software)	ThermoFisher Scientific	N/A
**Databases**		
BioGRID	Ref. [36]	https://thebiogrid.org/; RRID:SCR_007393
CORUM	Ref. [37]	https://mips.helmholtz-muenchen.de/corum/; RRID:SCR_002254
Complex portal	Ref. [38]	https://www.ebi.ac.uk/complexportal/home; RRID:SCR_015038

ENCODE	Ref. [39]	https://www.encodeproject.org/; RRID:SCR_015482
EpiFactors	Ref. [40]	http://epifactors.autosome.ru/; RRID:SCR_016956
Mygene.info	Ref. [41]	https://mygene.info/; RRID:SCR_018660
UniProt/Swiss-Prot	Ref. [42]	https://www.uniprot.org/; RRID:SCR_002380

Supplementary Material Legends

Supplementary Figure 1. Gel raw data and graph source data

Pages 1-2: Uncropped gel images for the immunoblots shown in Figure 5e, Extended Data Figure 2b, and Extended Data Figures 5g,h,j. Images were acquired by CCD camera using a BioRad ChemiDoc Touch Imaging System. Positions of molecular weight markers (kDa) are indicated next to the gels. Cropped parts of the gels used for the final figures are marked by frames. Page 3: Graph source data table providing the number of feature effect estimate measurements for the H3ac and H4ac features for each of the protein complexes displayed in the bar graph in Figure 3d.

Supplementary Table 1. Post-processed data of 55 SILAC di-nucleosome purification experiments

Quantification of protein responses in each of the 55 di-nucleosome purification experiments. Experiments are described in individual sheets of the Excel file. The index for the SNAP experiments is available in the first sheet. For each of the di-nucleosome purification experiments, post-processed and imputed SILAC $\log_2$ H/L ratios of the proteins are listed in columns along with their raw (unprocessed) inputs and intermediate estimates of enrichment (see Methods). Miscellaneous metadata about the measurements, including identification type, number of peptides detected, MS1 intensity, and sequence coverage is also provided.

Supplementary Table 2. Heatmap visualisation of the 55 SILAC di-nucleosome purification experiments

Clustered heatmap of SILAC $\log_2$ H/L ratios of all 1915 proteins detected in our SNAP experiments in Excel format. The heatmap is structured to mimic the layout of Fig. 1d-e. Additional metadata about proteins, including their names, UniProt identifiers, and complex memberships is available in the second worksheet. Information about the imputation status of each of the measurements is available in the third worksheet.

Supplementary Table 3. Predicted effect of chromatin modification features on protein binding

Estimates of chromatin feature effects for 15 nucleosomal features deconvoluted from the SNAP data corresponding directly to the volcano plots in Fig. 2b and Extended Data Fig. 3c-d and 5a. The data for chromatin features is provided in individual worksheets. Effect estimates and statistical parameters are provided in the columns. Rows are ordered by the FDR. Succinct profiles of feature effect estimates for individual proteins are also available in

Supplementary Table 7.

Supplementary Table 4. Integrative analysis of ENCODE NGS datasets and NIH Roadmap chromatin states with MARCS feature effect estimates

The summary table of the integrative analysis of MARCS and NGS datasets used in Extended Data Fig. 4a-j, Extended Data Fig. 7f, and Extended Data Fig. 5l. The identifiers of the datasets analysed are listed in the table, along with their links to MARCS data. The table provides the peak co-occurrence statistics (see online methods) for each of the combinations between protein datasets (rows) and chromatin features (columns), along with the final normalised mutual information and correlation estimates that were used in the figures.

Supplementary Table 5. Clustered groups of chromatin feature effect estimates

Clustered estimates of chromatin feature effects for proteins that respond significantly (limma, FDR ≤ 0.01) and strongly (estimated feature effect of at least 1.0) to one or more chromatin features. Effect estimates and Benjamini/Hochberg-adjusted p-values are provided in the columns. Rows are listed in the same order as Fig. 2e. See also Supplementary Table 3.

Supplementary Table 6. List of H3K4me1- and H3K4me3-associated proteins in IMR-90 cells

Label-free LC-MS quantification results for proteins identified in the anti-H3, anti-H4, anti-H3K4me1, and anti-H3K4me3 ChIP-MS experiments performed using double crosslinked IMR-90 chromatin. For each experiment the RAW as well as $\log_2$ transformed normalised protein abundances are provided. Cross-comparisons of protein abundances between the experiments ($\log_2$ FC), differentially enriched proteins (limma, two-sided, Benjamini/Hochberg-adjusted FDR ≤ 0.05), as well as the corresponding MARCS feature effect estimates are provided in the columns.

Supplementary Table 7. List of protein-protein interaction predictions and their responses to chromatin modification features

List of CLR predicted protein-protein interactions corresponding to Extended Data Fig. 7e and 8 are provided in the first worksheet of the Excel file. Putative interactions are stratified into five decreasing confidence levels ranging from high-confidence (corresponding to Extended Data Fig. 8) to q-value cut-off of 0.05. Putative interactions with confidence lower than q=0.05 were not listed but can be downloaded from the MARCS online resource (https://marcs.helmholtz-munich.de). Known interactions annotated in BioGRID are additionally annotated with corresponding metadata, while putative interactions unmatched in

BioGRID are marked as such. Interactions excluded from the network evaluation (see methods) are marked as excluded. The second sheet contains the information about the network nodes (i.e. the proteins) which includes their coordinates in the embedding (Extended Data Fig. 7e) as well as succinct profiles of chromatin feature effect estimates for each of the proteins profiled which corresponds directly to Fig. 2e.

Supplementary Table 8. List of protein complexes and their predicted chromatin responses

Curated list of chromatin complexes whose subunits can be detected in our data together with the sources for the annotations. In cases where we could detect the complex only partially, the missing subunits have a source record but no MARCS identifier assigned. Additionally, median chromatin feature effect estimates for the complexes (related to Fig. 3d, 5d and Extended Data Fig. 2d) are listed in the subsequent worksheets together with the corresponding CAMERA statistics.

Supplementary Table 9. Label-free MS quantification results of protein binding responses to di-nucleosomes incorporating various linker DNAs

The table summarises the RAW MS quantification results and post-processed data for the affinity purification pull-downs with di-nucleosomes incorporating different linker DNAs described in Fig. 4a. The data for the three different independent sets of pull-downs are presented in individual sheets in the Excel file. The first sheet provides the results for the pull-downs with di-nucleosomes decorated with heterochromatin-associated modifications and incorporating 35-55bp long linkers; the second sheet provides the results for the di-nucleosomes decorated with promoter-associated modifications and incorporating 200bp scrambled DNA and SV40 promoter sequence-based linkers; the third sheet provides the results for the di-nucleosomes decorated with enhancer-associated modifications and incorporating 200bp scrambled DNA and SV40 enhancer sequence-based linkers. For each experiment the RAW and log_2-transformed normalised protein abundances are provided. Cross-comparison of protein abundances between different pull-downs (log_2 FC), differentially abundant proteins (between specified pull-downs) and proteins responding to either linker or modifications and both features (limma, two-sided, Benjamini/Hochberg-adjusted FDR < 0.05) as well as the results of the hierarchical clustering presented in Fig. 4b, Extended Data Fig. 9a and 10a are provided in the columns.

Supplementary Table 10. Key resources table

A list of key resources including reagents, software, and databases used in this study.

References (Supplementary Information)

1. Trojer, P. & Reinberg, D. Facultative Heterochromatin: Is There a Distinctive Molecular Signature? *Mol Cell* **28**, 1–13 (2007).

2. Saksouk, N., Simboeck, E. & Déjardin, J. Constitutive heterochromatin formation and transcription in mammals. *Epigenet Chromatin* **8**, 3 (2015).

3. Giaimo, B. D., Ferrante, F., Herchenröther, A., Hake, S. B. & Borggrefe, T. The histone variant H2A.Z in gene regulation. *Epigenet Chromatin* **12**, 37 (2019).

4. Saredi, G. *et al.* H4K20me0 marks post-replicative chromatin and recruits the TONSL–MMS22L DNA repair complex. *Nature* **534**, 714–718 (2016).

5. Sobel, R. E., Cook, R. G., Perry, C. A., Annunziato, A. T. & Allis, C. D. Conservation of deposition-related acetylation sites in newly synthesized histones H3 and H4. *Proc National Acad Sci* **92**, 1237–1241 (1995).

6. Loyola, A., Bonaldi, T., Roche, D., Imhof, A. & Almouzni, G. PTMs on H3 Variants before Chromatin Assembly Potentiate Their Final Epigenetic State. *Mol Cell* **24**, 309–316 (2006).

7. Meneghini, M. D., Wu, M. & Madhani, H. D. Conserved Histone Variant H2A.Z Protects Euchromatin from the Ectopic Spread of Silent Heterochromatin. *Cell* **112**, 725–736 (2003).

8. Young, N. L. *et al.* High Throughput Characterization of Combinatorial Histone Codes. *Mol Cell Proteomics* **8**, 2266–2284 (2009).

9. Sidoli, S. *et al.* Middle-down hybrid chromatography/tandem mass spectrometry workflow for characterization of combinatorial post-translational modifications in histones. *Proteomics* **14**, 2200–11 (2014).

10. Lowary, P. T. & Widom, J. New DNA sequence rules for high affinity binding to histone octamer and sequence-directed nucleosome positioning. *J Mol Biol* **276**, 19–42 (1998).

11. Dorigo, B., Schalch, T., Bystricky, K. & Richmond, T. J. Chromatin Fiber Folding: Requirement for the Histone H4 N-terminal Tail. *J Mol Biol* **327**, 85–96 (2003).

12. Dyer, P. N. *et al.* Reconstitution of Nucleosome Core Particles from Recombinant Histones and DNA. *Methods Enzymol* **375**, 23–44 (2003).

13. Hein, M. Y. *et al.* A Human Interactome in Three Quantitative Dimensions Organized by Stoichiometries and Abundances. *Cell* **163**, 712–723 (2015).

14. Bartke, T. *et al.* Nucleosome-Interacting Proteins Regulated by DNA and Histone Methylation. *Cell* **143**, 470–484 (2010).

15. Nakamura, K. *et al.* H4K20me0 recognition by BRCA1–BARD1 directs homologous recombination to sister chromatids. *Nat Cell Biol* **21**, 311–318 (2019).

16. Foster, B. M. *et al.* Critical Role of the UBL Domain in Stimulating the E3 Ubiquitin Ligase Activity of UHRF1 toward Chromatin. *Mol Cell* **72**, 739-752.e9 (2018).

17. Quinlan, A. R. & Hall, I. M. BEDTools: a flexible suite of utilities for comparing genomic features. *Bioinformatics* **26**, 841–842 (2010).

18. Franzin, A., Sambo, F. & Camillo, B. D. bnstruct: an R package for Bayesian Network structure learning in the presence of missing data. *Bioinformatics* btw807 (2016) doi:10.1093/bioinformatics/btw807.

19. Wu, D. & Smyth, G. K. Camera: a competitive gene set test accounting for inter-gene correlation. *Nucleic Acids Res* **40**, e133 (2012).

20. Shannon, P. *et al.* Cytoscape: A Software Environment for Integrated Models of Biomolecular Interaction Networks. *Genome Res* **13**, 2498–2504 (2003).

21. Faith, J. J. *et al.* Large-Scale Mapping and Validation of Escherichia coli Transcriptional Regulation from a Compendium of Expression Profiles. *Plos Biol* **5**, e8 (2007).

22. Bastian, M., Heymann, S. & Jacomy, M. Gephi: an open source software for exploring and manipulating networks. *Proceedings of the International AAAI Conference on Web and Social Media* **3**:361-362 (2009).

23. Ritchie, M. E. *et al.* limma powers differential expression analyses for RNA-sequencing and microarray studies. *Nucleic Acids Res* **43**, e47 (2015).

24. Perkins, D. N., Pappin, D. J. C., Creasy, D. M. & Cottrell, J. S. Probability-based protein identification by searching sequence databases using mass spectrometry data. *Electrophoresis* **20**, 3551–3567 (1999).

25. Hunter, J. D. Matplotlib: A 2D Graphics Environment. *Comput Sci Eng* **9**, 90–95 (2007).

26. Cox, J. & Mann, M. MaxQuant enables high peptide identification rates, individualized p.p.b.-range mass accuracies and proteome-wide protein quantification. *Nat Biotechnol* **26**, 1367–1372 (2008).

27. Meyer, P. E., Lafitte, F. & Bontempi, G. minet: A R/Bioconductor Package for Inferring Large Transcriptional Networks Using Mutual Information. *BMC Bioinformatics* **9**, 461 (2008).

28. Hagberg, A., Swart, P. & Chult, D. S. *Exploring network structure, dynamics, and function using NetworkX*. (2008). https://www.osti.gov/servlets/purl/960616

29. Vallat, R. Pingouin: statistics in Python. *J Open Source Softw* **3**, 1026 (2018).

30. Saito, T. & Rehmsmeier, M. Precrec: fast and accurate precision–recall and ROC curve calculations in R. *Bioinformatics* **33**, 145–147 (2016).

31. Bien, J. & Tibshirani, R. Hierarchical Clustering With Prototypes via Minimax Linkage. *J Am Stat Assoc* **106**, 1075–1084 (2012).

32. Pedregosa, F. *et al.* Scikit-learn: Machine Learning in Python. *Journal of Machine Learning Research* **12**, 2825-2830 (2011).

33. Virtanen, P. *et al.* SciPy 1.0: fundamental algorithms for scientific computing in Python. *Nat Methods* **17**, 261–272 (2020).

34. MacLean, B. *et al.* Skyline: an open source document editor for creating and analyzing targeted proteomics experiments. *Bioinformatics* **26**, 966–968 (2010).

35. Seabold, S. & Perktold, J. statsmodels: Econometric and statistical modeling with python. in *Proc of the 9th Python in Science Conf*. (2010).

36. Oughtred, R. *et al.* The BioGRID interaction database: 2019 update. *Nucleic Acids Res* **47**, D529–D541 (2018).

37. Giurgiu, M. *et al.* CORUM: the comprehensive resource of mammalian protein complexes—2019. *Nucleic Acids Res* **47**, D559–D563 (2018).

38. Meldal, B. H. M. *et al.* The complex portal - an encyclopaedia of macromolecular complexes. *Nucleic Acids Res* **43**, D479–D484 (2014).

39. Dunham, I. *et al.* An Integrated Encyclopedia of DNA Elements in the Human Genome. *Nature* **489**, 57–74 (2012).

40. Medvedeva, Y. A. *et al.* EpiFactors: a comprehensive database of human epigenetic factors and complexes. *Database* **2015**, bav067 (2015).

41. Xin, J. *et al.* High-performance web services for querying gene and variant annotation. *Genome Biol* **17**, 91 (2016).

42. Bateman, A. *et al.* UniProt: a worldwide hub of protein knowledge. *Nucleic Acids Res* **47**, D506–D515 (2018).

Fig. 5e

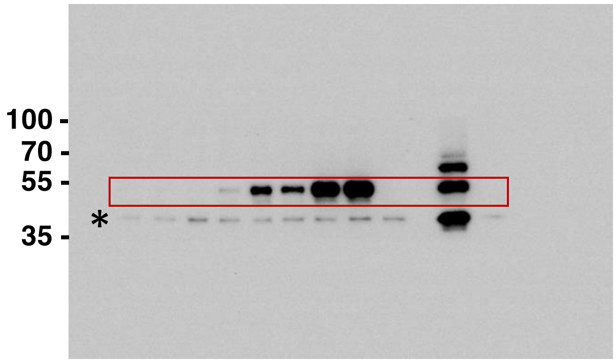

anti-INO80B

* Non specific signal

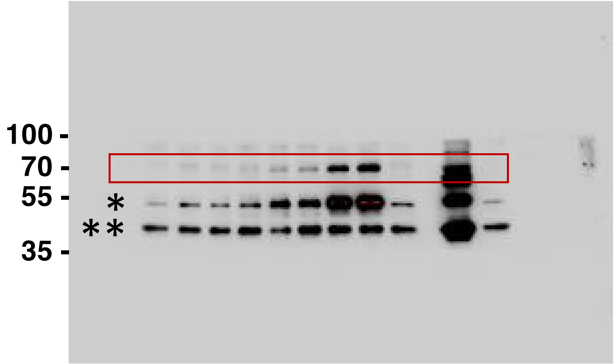

anti-ACTR5

* anti-INO80B; ** Non specific signal

Note: INO80B and ACTR5 WBs were performed sequentially using the same membrane

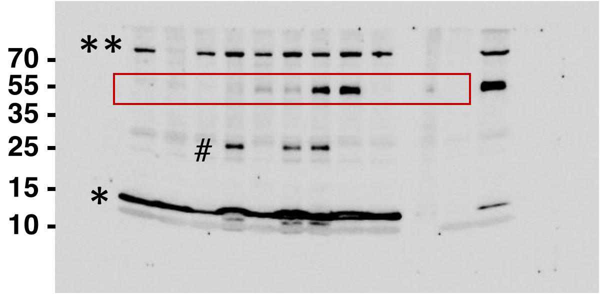

anti-TBRG1

* anti-H4

\# , ** Non specific signal

Note: TBRG1 and H4 were probed on a separate membrane.

Extended Data Fig. 2b

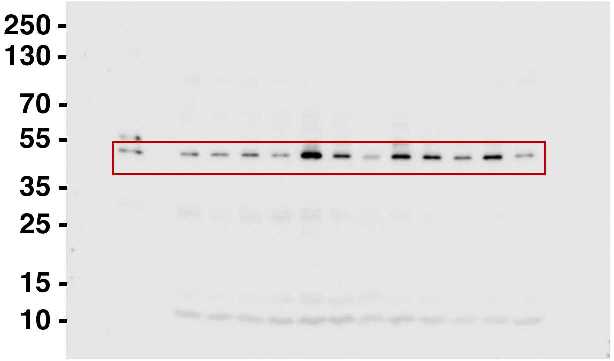

anti-CBX8

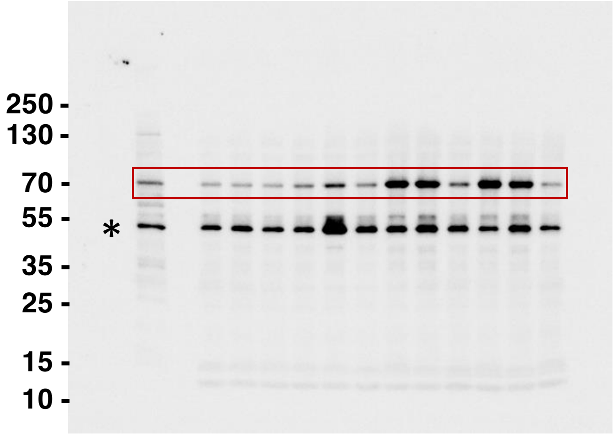

anti-CBX4

* anti-CBX8

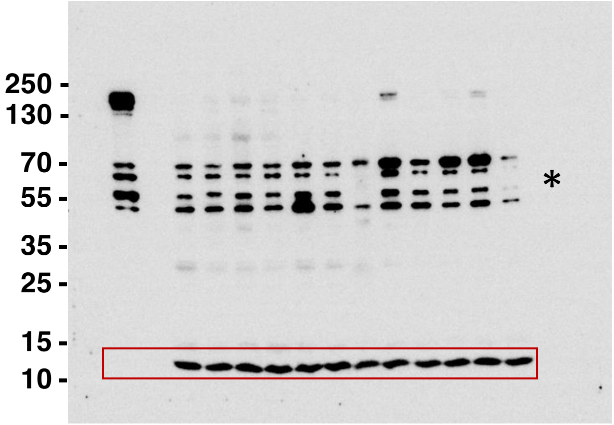

anti-H4

* anti-CBX4 & CBX8

Note: CBX4, CBX8 and H4 were probed sequentially using the same membrane.

Extended Data Fig. 5h

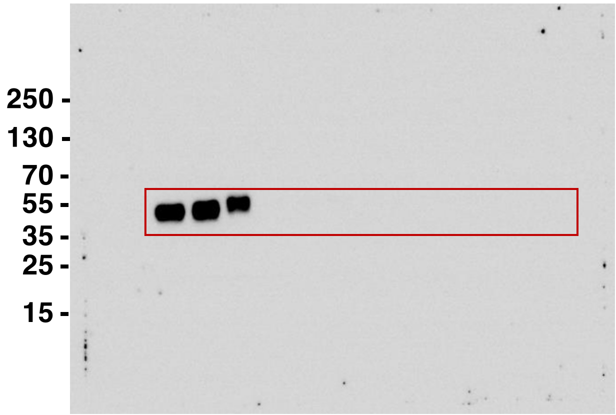

anti-V5 (INO80B)

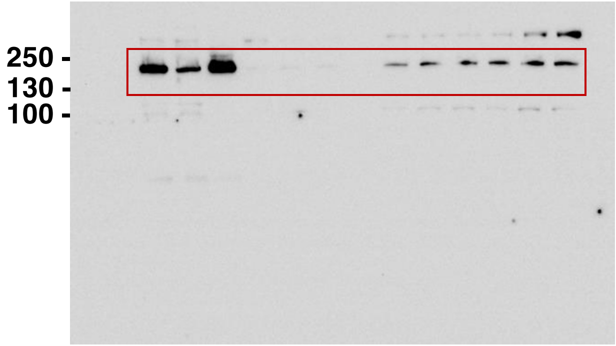

anti-INO80

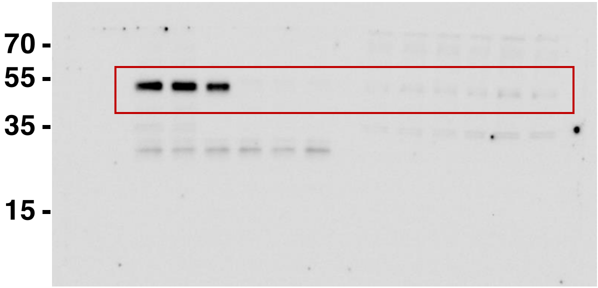

anti-TBRG1

Extended Data Fig. 5j

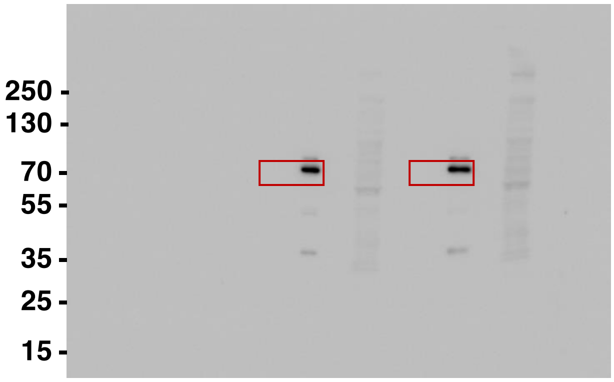

anti-ACTR5

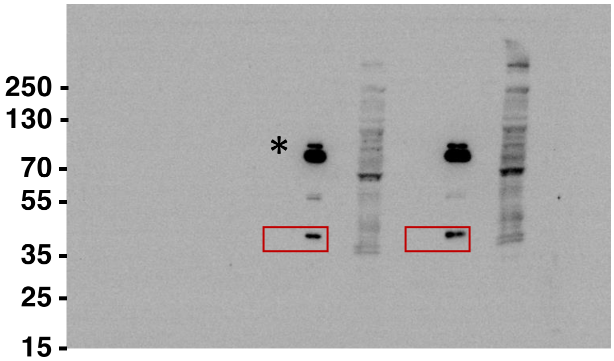

anti-INO80B * anti-ACTR5

Note: INO80B and ACTR5 were probed simultaneously using the same membrane

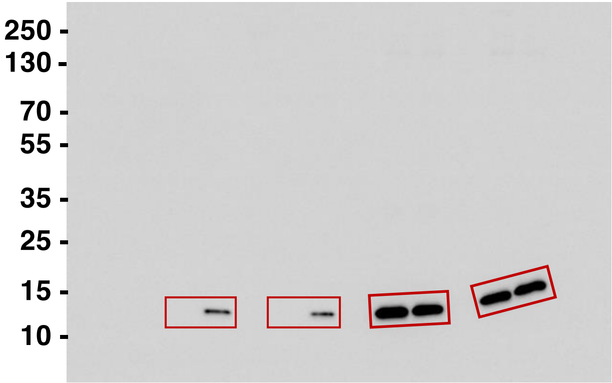

anti-H2B

Note: H2B was probed on a different membrane

Extended Data Fig. 5g

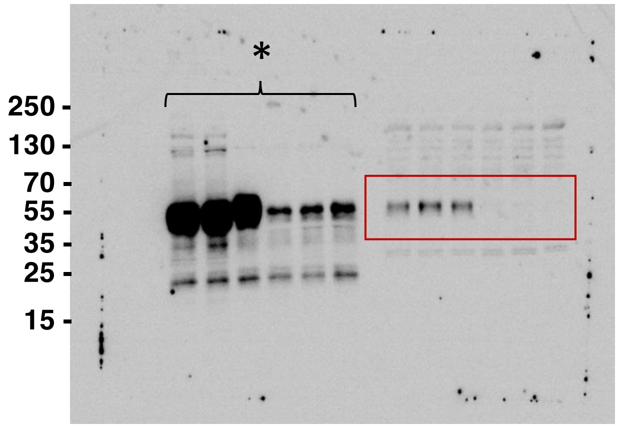

anti-V5 (INO80B)

Note: WB obtained by a longer exposure of the same membrane as shown in Extended Data Fig. 5h

* The first six lanes are IP samples corresponding to the experiment shown in Extended Data Fig. 5h

Sample size values for Fig. 3d

	Complex																										
**predictor**	**PBAF**	**BAF**	**EBAFA/B**	**GBAF**	**ATAC (exclusive subunits)**	**MLL3/4 (exclusive subunits)**	**ATAC**	**MLL3/4**	**NuRF**	**NuRD (exclusive subunits)**	**SAGA**	**NuRD**	**PCAF**	**TFTC**	**NuA4**	**NuA4 (exclusive subunits)**	**TFIID**	**SRCAP**	**MOZ/MORF**	**PRC1 (exclusive subunits)**	**PRC1**	**Integrator**	**Integrator (exclusive subunits)**	**INO80**	**Mediator**	**INO80 (exclusive subunits)**	**CHRAC**
**H3ac**	13	10	9	12	6	6	11	10	4	9	20	14	9	20	20	6	14	9	5	5	7	14	13	15	15	11	4
**H4ac**	16	13	12	12	6	6	11	10	4	9	20	14	9	20	20	6	13	9	5	5	7	13	12	15	15	11	4

Note: all other relevant information can be found in Supplementary Table 8.
