# Supplementary material for: Decoding chromatin states by proteomic profiling of nucleosome readers
Source: Nature. 2024 Mar 6;627(8004):671–9. doi: 10.1038/s41586-024-07141-5 (PMC10954555; doi:10.1038/s41586-024-07141-5)
Supplement: Supplementary file 2 — Reporting Summary [file 41586_2024_7141_MOESM2_ESM.pdf]

Corresponding author(s): Till Bartke

Last updated by author(s): Dec 13, 2023

Reporting Summary

Nature Portfolio wishes to improve the reproducibility of the work that we publish. This form provides structure for consistency and transparency in reporting. For further information on Nature Portfolio policies, see our Editorial Policies and the Editorial Policy Checklist.

Statistics

For all statistical analyses, confirm that the following items are present in the figure legend, table legend, main text, or Methods section.

n/a	Confirmed	
☐	☒	The exact sample size (*n*) for each experimental group/condition, given as a discrete number and unit of measurement
☐	☒	A statement on whether measurements were taken from distinct samples or whether the same sample was measured repeatedly
☐	☒	The statistical test(s) used AND whether they are one- or two-sided<br>*Only common tests should be described solely by name; describe more complex techniques in the Methods section.*
☒	☐	A description of all covariates tested
☐	☒	A description of any assumptions or corrections, such as tests of normality and adjustment for multiple comparisons
☐	☒	A full description of the statistical parameters including central tendency (e.g. means) or other basic estimates (e.g. regression coefficient) AND variation (e.g. standard deviation) or associated estimates of uncertainty (e.g. confidence intervals)
☐	☒	For null hypothesis testing, the test statistic (e.g. *F*, *t*, *r*) with confidence intervals, effect sizes, degrees of freedom and *P* value noted<br>*Give P values as exact values whenever suitable.*
☒	☐	For Bayesian analysis, information on the choice of priors and Markov chain Monte Carlo settings
☒	☐	For hierarchical and complex designs, identification of the appropriate level for tests and full reporting of outcomes
☐	☒	Estimates of effect sizes (e.g. Cohen's *d*, Pearson's *r*), indicating how they were calculated

Our web collection on statistics for biologists contains articles on many of the points above.

Software and code

Policy information about availability of computer code

Data collection

Mass spectrometry data was collected on Thermo Scientific Q-Exactive (SNAP experiments) and Q-Exactive HF (label-free nucleosome pull-downs, IP-MS, ChIP-MS, and MS of modified histone samples) mass spectrometers using Thermo Scientific Xcalibur Software. Western blot images were acquired by CCD camera using a BioRad ChemiDoc Touch Imaging System running Image Lab Touch Software (v2.3.0.07).

Data analysis

The data analysis scripts for this study are available at GitHub at locations https://github.com/lukauskas/publications-lukauskas-2024-marcs (source code for main scripts) and https://github.com/lukauskas/marcs (source code for the interactive MARCS interface).

Key software for data analysis and data visualisation that was used in this manuscript are:
- Bedtools (v2.30.0) - NGS data analysis
- Bnstruct (R package, v1.0.8) - data analysis
- CAMERA (part of limma R package; see limma for version number) - statistical analysis
- Cytoscape (v3.7.1) - network visualisation and analysis
- CLR (part of minet R package; see minet for version number) - network inference
- Gephi (v0.9.2) - network analysis and visualisation
- limma (R package, v3.42.2, v3.50.1) - statistical analysis
- Mascot (v2.6.2) - search engine for protein identification from proteomic mass spectrometry data
- Matplotlib (Python package, v3.0.3, v3.4.3, v3.5.1) - data visualisation
- MaxQuant (v1.5.2.8) - proteomic quantification
- minet (R package, v3.44.1) - network inference
- NetworkX (Python package, v2.3) - network visualisation and analysis
- Pingouin (Python package, v0.5.1) - statistical analysis

• Precrec (R package, v0.11) - statistical analysis
• Progenesis QI (v4.1) - protein quantification of proteomic mass spectrometry
• Proteome Discoverer (v2.5) - protein quantification of proteomic mass spectrometry
• Protoclust (R package, v1.6.3) - data analysis and visualisation
• python-louvain (Python package, v0.13) - network analysis and visualisation
• Scikit-learn (Python package, v0.21.1) - data analysis and visualisation
• Scipy (Python package, v1.3.0, v1.7.1) - data analysis and visualisation
• Skyline (v20.1.0.31) - identification and quantification of histone PTMs for histone mass spectrometry data
• Statsmodels (Python package, v0.9.0, v0.12.2, v0.13.2) - statistical analysis

Detailed information about software used in this manuscript is provided in the Key Resources Table in Supplementary Table 10 and the Supplementary Information.

For manuscripts utilizing custom algorithms or software that are central to the research but not yet described in published literature, software must be made available to editors and reviewers. We strongly encourage code deposition in a community repository (e.g. GitHub). See the Nature Portfolio guidelines for submitting code & software for further information.

Data

Policy information about availability of data

All manuscripts must include a data availability statement. This statement should provide the following information, where applicable:

- Accession codes, unique identifiers, or web links for publicly available datasets
- A description of any restrictions on data availability
- For clinical datasets or third party data, please ensure that the statement adheres to our policy

Gel source raw data for western blots shown in Figure 5e, Extended Data Figure 2b, and Extended Data Figures 5g,h,j is provided in Supplementary Figure 1.

The mass spectrometry data that was generated for this study has been deposited to the ProteomeXchange Consortium via the PRIDE partner repository (https://www.ebi.ac.uk/pride/) with the following identifiers:
• SILAC di-nucleosome purification experiments: PXD018966 including the H4K20me2 samples from this experiment which were previously deposited with identifier PXD009281
• H3K4me1 and H3K4me3 ChIP-MS (analysis of histone PTMs): PXD042224
• H3K4me1 and H3K4me3 ChIP-MS (analysis of co-purified proteins): PXD042826
• Label-free di-nucleosome purification experiments with 200bp SV40 promoter linker: PXD041835
• Label-free di-nucleosome purification experiments with 200bp SV40 enhancer linker: PXD041443
• Label-free di-nucleosome purification experiments with short linkers and heterochromatic PTMs: PXD042368
• IP-MS analysis of the human INO80 complex composition and interactome: PXD020712
• ChIP-MS analysis of histone PTMs co-purified with the human INO80 complex: PXD042210
• Analysis of the effect of native chemical ligation on protein binding: PXD042390
• Mass spectrometric analysis of ligated and recombinant human histones H3 and H4: PXD020773
• Analysis of the stability of nucleosomal modifications during affinity purification in nuclear extract: PXD042823

Additionally, the SILAC nucleosome affinity purification data is available in interactive format at https://marcs.helmholtz-munich.de.

Public databases that were used for data analysis in this study are:
• BioGRID - https://thebiogrid.org/
• CORUM - https://mips.helmholtz-muenchen.de/corum/
• Complex portal - https://www.ebi.ac.uk/complexportal/home
• ENCODE - https://www.encodeproject.org/
• EpiFactors - http://epifactors.autosome.ru/
• Mygene.info - https://mygene.info/
• UniProt/Swiss-Prot - https://www.uniprot.org/

Further information about databases used in this study is provided in the Key Resources Table in Supplementary Table 10 and the Supplementary Information. A detailed list of ENCODE datasets used for the integration of MARCS with ChIP-seq data, including ENCODE accession numbers, is provided in Supplementary Table 4.

Research involving human participants, their data, or biological material

Policy information about studies with human participants or human data. See also policy information about sex, gender (identity/presentation), and sexual orientation and race, ethnicity and racism.

Reporting on sex and gender	This study did not involve human participants, their data or biological material.
Reporting on race, ethnicity, or other socially relevant groupings	This study did not involve human participants, their data or biological material.
Population characteristics	This study did not involve human participants, their data or biological material.
Recruitment	This study did not involve human participants
Ethics oversight	This study did not require ethical approval

Note that full information on the approval of the study protocol must also be provided in the manuscript.

Field-specific reporting

Please select the one below that is the best fit for your research. If you are not sure, read the appropriate sections before making your selection.

☒ Life sciences ☐ Behavioural & social sciences ☐ Ecological, evolutionary & environmental sciences

For a reference copy of the document with all sections, see nature.com/documents/nr-reporting-summary-flat.pdf

Life sciences study design

All studies must disclose on these points even when the disclosure is negative.

Sample size	The nucleosomes tested in SILAC nucleosome affinity purification experiments were selected based on the prior reports of biologically relevant modification signatures of chromatin states. It was attempted to achieve a good coverage of modification signatures representing enhancer, promoter, and different heterochromatin states, while balancing this with the requirements for single modification controls and the limited availability of nuclear extracts. Studies that were used as information sources for the general design of the library of modified nucleosomes include: Young et al., 2009, Mol Cell Proteomics 8: 2266–2284; Sidoli et al., 2014, Proteomics 14: 2200–11; Kundaje et al., 2015, Nature 518: 317–330. In total 55 di-nucleosomes were assembled. Repressive nucleosomes were designed to contain di- and tri-methylation of lysines 9 and 27 of histone H3 (H3K9me2/3 and H3K27me2/3), that mark constitutive and facultative heterochromatin, and tri-methylation of lysine 20 of histone H4 (H4K20me3) that marks pericentric and telomeric heterochromatin (Trojer & Reinberg, 2007, Mol Cell 28: 1–13; Saksouk et al., 2015, Epigenet Chromatin 8: 3). Combinations of these tri-methyl marks and CpG-methylated DNA were used to test for cross-talk between silencing pathways, and between heterochromatic histone modifications and DNA methylation. Euchromatic nucleosomes were decorated with mono- and tri-methylation of lysine 4 of histone H3 in order to profile enhancer- (H3K4me1) and promoter- (H3K4me3) like chromatin states. Both were combined with varying degrees of acetylation of lysines (Kac) in the H3 and H4 N-terminal tails, and the histone variant H2A.Z due to its emergent role at enhancers and promoters (Giaimo et al., 2019, Epigenet Chromatin 12: 37). Di-methylation of lysine 20 of histone H4 (H4K20me2) was added to a subset of both repressive and activating nucleosomes as it is a pervasive modification present throughout the genome (Saredi et al., 2016, Nature 534: 714–718). In addition to repressive and activating modification signatures H4K5acK12ac that marks newly deposited histones (Sobel et al., 1995, Proc National Acad Sci 92: 1237–1241; Loyola et al., 2006, Mol Cell 24: 309–316), H2A.Z that also has a function as a regulator of heterochromatin boundaries (Meneghini et al., 2003, Cell 112: 725–736), and three nucleosomes combining H3K4me1 with H3K27me3 and low levels of H3 acetylation were included, as such species were detected by mass spectrometry (Young et al., 2009, Mol Cell Proteomics 8: 2266–2284; Sidoli et al., 2014, Proteomics 14: 2200–11). Detailed information about the design of the library of modified nucleosomes is provided in the Supplementary Information.<br><br>Label-free nucleosome affinity purifications using di-nucleosomes incorporating different DNA linkers followed two design strategies: (1) to test the effect of DNA linker length on protein binding to heterochromatin-like chromatin states (Trojer & Reinberg, 2007, Mol Cell 28: 1–13; Saksouk et al., 2015, Epigenet Chromatin 8: 3) di-nucleosomes incorporating H3K9me3 or H3K27me3 modifications (markers of constitutive and facultative heterochromatin, respectively) were assembled with a series of short DNA linkers increasing in length from 35-55 bp in 5bp increments, since these are the most frequently found linker lengths in mammalian cells (Voong et al., 2016, Cell 167: 1555-1570.e15); (2) to test the effects of the presence of a nucleosome-depleted region on protein binding to enhancer- and promoter-like chromatin states, di-nucleosomes incorporating either H3K4me1 or H3K4me1K27ac modifications and a 200bp DNA linker containing the functional elements of the SV40 early enhancer (enhancer chromatin state), or H3K4me3K9acK14acK18acK23acK27ac and H4K5acK8acK12acK16acK20me2 modifications and the histone variant H2A.Z together with a 200bp DNA linker containing the SV40 early core promoter (active promoter chromatin state) were assembled and compared to the respective unmodified di-nucleosomes and a library of di-nucleosomes containing 100 million different 200 bp DNA linkers with random sequences. The viral SV40 promoter and enhancer sequences were chosen since they constitute very well characterised enhancer and promoter sequences (Banerji et al., 1981, Cell 27: 299-308; Schirm et al., 1987, Genes Dev 1: 65-74; Keiser et al., 2015, J Gen Virol 96: 601-606), and both are around 200 bp in length, enabling the assembly of di-nucleosomes with 200 bp nucleosome-depleted regions resembling enhancer- and promoter-like chromatin states (Haberle & Lenhard, 2016, Semin Cell Dev Biol 57: 11-23; Haberle & Stark, 2019, Nat Rev Mol Cell Biol 19: 621-637). Detailed information about the design of modified di-nucleosomes incorporating various different DNA linkers is provided in the Supplementary Information.
Data exclusions	• One di-nucleosome in the SILAC nucleosome affinity purification experiments in which we profiled H3K4me3-5ac/H4-4ac/H2A.Z in combination with methylated DNA failed our quality checks for the nucleosome assembly described in the supplementary document. This nucleosome was therefore not included in our experiments and analyses.<br>• One measurement in the label-free di-nucleosome affinity purification experiments in which we profiled H3K27me3 in combination with 35 bp linker DNA failed our mass spectrometry data quality checks. This measurement was therefore excluded from our statistical analyses.<br>• A series of label-free di-nucleosome affinity purifications in which we tested combinations of H3K27ac with 50bp, 200bp scrambled, and 200bp SV40 enhancer linkers was carried out and analysed together with the affinity purifications testing combinations of H3K4me1 and H3K4me1K27ac with 50bp, 200bp scrambled, and 200bp SV40 enhancer linkers. Since the H3K27ac affinity purifications did not add any additional valuable information, they were not included in the final figures in order to reduce the complexity of the displayed data.
Replication	• For the SNAP experiments the two isotopically labelled batches of nuclear extracts allow each of the experiments to be performed as a biological replicate (in forward and reverse settings, see methods). The extracts were mixtures of three independently prepared extracts to level out differences in individual extracts. Multiple nucleosomes with similar modification patterns serve as internal controls.<br>• Label-free nucleosome affinity purifications, IP-MS, and X-ChIP-MS experiments were carried out in triplicates to allow statistical analyses.<br>• Native ChIP-MS of INO80-bound nucleosomes was performed in two independent replicates with similar results in each replicate.<br>• Nucleosome affinity purifications followed by western blot detection to validate the nucleosome binding characteristics of INO80 (Figure 5e) were carried out in three independent experimental replicates with similar results in each replicate.<br>• Nucleosome affinity purifications followed by western blot detection to validate the nucleosome binding characteristics of CBX4 and CBX8 (Extended Data Figure 2b) were carried out in two experimental replicates with similar results in both replicates.
Randomization	Samples were not randomised. However, being a high-throughput proteomics study, many samples were handled at any given time and for

Randomization	each set of experiments with no particular preference or bias towards specific samples. During the experiments care was taken to treat batches of samples evenly by rotating the order of the samples during repeated processing steps. During the mass spectrometric measurements the liquid chromatography columns were cleaned in regular intervals between runs, and replicate samples belonging to the same set of experiments were injected in random order to avoid any measurement biases. Batch effects and experimental biases were also minimised during the downstream computational analyses by normalisation and cross-validation of all measurements of a given dataset.
Blinding	Investigators were not blinded. Blinding was not required since the outcome of the high-throughput proteomics measurements and western blot readouts are unknown to the experimenter at the time of performing the experiment and carrying out the data acquisition. The results can therefore not be affected by personal bias or knowledge of the identity of the sample at the time of acquiring the data.

Reporting for specific materials, systems and methods

We require information from authors about some types of materials, experimental systems and methods used in many studies. Here, indicate whether each material, system or method listed is relevant to your study. If you are not sure if a list item applies to your research, read the appropriate section before selecting a response.

Materials & experimental systems

n/a	Involved in the study	
☐	☒	Antibodies
☐	☒	Eukaryotic cell lines
☒	☐	Palaeontology and archaeology
☒	☐	Animals and other organisms
☒	☐	Clinical data
☒	☐	Dual use research of concern
☒	☐	Plants

Methods

n/a	Involved in the study	
☒	☐	ChIP-seq
☒	☐	Flow cytometry
☒	☐	MRI-based neuroimaging

Antibodies

Antibodies used

Anti-V5 tag (eBioscience, TCM5 #14-6796-82)
Anti-V5 tag (Abcam, ab15828)
Anti-INO80 (Abcam, ab118787)
Anti-INO80B (Santa Cruz (E-3), sc-390009)
Anti-ACTR5 (GeneTex, GTX80453)
Anti-TBRG1 (Santa Cruz (D-9), sc-515620)
Anti-H3 (Active motif, 39163)
Anti-H3K4me1 (Abcam, ab8895)
Anti-H3K4me3 (Millipore, 17-614)
Anti-H4 (Abcam, ab31830)
Anti-H4ac (pan-acetyl) (Active Motif, 39967)
Anti-CBX4 (Cell Signaling Technology, E6L7X #30559)
Anti-CBX8 (Santa Cruz (C-3), sc-374332)
Anti-H2B (Abcam, ab1790)
Anti-H2A.Z (Abcam, ab4174)
Alexa Fluor® 488 Anti-Mouse IgG (Jackson ImmunoResearch Laboratories, 715-545-150)

Validation

All antibodies are commercially available and were validated for the specified applications by the suppliers (supplier information):
• Anti-V5 tag (eBioscience, TCM5 #14-6796-82) - species reactivity: tag; validated applications: Western Blot (dil. 1:1000), Immunocytochemistry (dil. 1:100)
• Anti-V5 tag (Abcam, ab15828) - species reactivity: tag; validated applications: Western Blot (0.5 µg/ml), Chromatin Immunoprecipitation (assay-dependent dilution)
• Anti-INO80 (Abcam, ab118787) - species reactivity: human; validated applications: Western Blot (dil. 1:2.000 - 1:10.000), Immunoprecipitation (use at 2-10 µg/mg of lysate)
• Anti-INO80B (Santa Cruz (E-3), sc-390009) - species reactivity: human, mouse, rat; validated applications: Western Blot (dil. 1:100 - 1:1.000), Immunoprecipitation (1-2 µg per 100-500 µg of total protein (1 ml of cell lysate)), Immunofluorescence (dil. 1:50 - 1:500), ELISA (dil. 1:30 - 1:3.000)
• Anti-ACTR5 (GeneTex, GTX80453) - species reactivity: human, hamster; validated applications: Western Blot (dil. 1:1000), Immunohistochemistry (dil. 1:50 - 1:100)
• Anti-TBRG1 (Santa Cruz (D-9), sc-515620) - species reactivity: human, mouse, rat; validated applications: Western Blot (dil. 1:100 - 1:1.000), Immunoprecipitation (1-2 µg per 100-500 µg of total protein (1 ml of cell lysate)), Immunocytochemistry/Immunofluorescence (dil. 1:50 - 1:500), ELISA (dil. 1:30 - 1:3.000)
• Anti-H3 (Active motif, 39163) - species reactivity: human, budding yeast, other (wide range); validated applications: Western Blot (dil. 1:5.000 - 1:15.000), Chromatin Immunoprecipitation (ChIP) and ChIP-seq (5 - 15 µl per ChIP)
• Anti-H3K4me1 (Abcam, ab8895) - species reactivity: human, mouse, rat, cow; validated applications: Western Blot (dil. 1:500), Chromatin Immunoprecipitation (2 µg for 25 µg of chromatin), Immunocytochemistry/Immunofluorescence (1 - 5 µg/ml), Immunohistochemistry (0.5 µg/ml)
• Anti-H3K4me3 (Millipore, 17-614) - species reactivity: human, mouse, mammals; validated applications: Western Blot (dil. 1:2.000), Chromatin Immunoprecipitation (ChIP) and ChIP-seq (3 µl per ChIP - chromatin from 1-3 X 10^6 cell equivalents)
• Anti-H4 (Abcam, ab31830) - species reactivity: human, cow; validated applications: Western Blot (1 µg/ml), Chromatin Immunoprecipitation (5 µg for 25 µg of chromatin), Immunocytochemistry/Immunofluorescence (5 µg/ml), Immunohistochemistry

(0.05 - 1 µg/ml), Flow Cytometry (1 µg for 10^6 cells)
• Anti-H4ac (pan-acetyl) (Active Motif, 39967) - species reactivity: human, wide range predicted; validated applications: Western Blot (dil. 1:1000), Immunocytochemistry, Immunofluorescence, Immunohistochemistry
• Anti-CBX4 (Cell Signaling Technology, E6L7X #30559) - species reactivity: human, mouse, monkey; validated applications: Western Blot (dil. 1:1.000), Immunoprecipitation (dil. 1.100), Chromatin Immunoprecipitation (ChIP) and ChIP-seq (dil. 1:50; 10 µl per 10 µg of chromatin), CUT&RUN (dil. 1:50), Immunocytochemistry/Immunofluorescence (dil. 1:200)
• Anti-CBX8 (Santa Cruz (C-3), sc-374332) - species reactivity: human, mouse, rat; validated applications: Western Blot (dil. 1:100 - 1:1.000), Immunoprecipitation (1-2 µg per 100-500 µg of total protein (1 ml of cell lysate)), Immunocytochemistry/ Immunofluorescence (dil. 1:50 - 1:500), ELISA (dil. 1:30 - 1:3.000)
• Anti-H2B (Abcam, ab1790) - species reactivity: human, cow, Saccharomyces cerevisiae, Xenopus laevis, Arabidopsis thaliana; validated applications: Western Blot (0.1 µg/ml), Immunoprecipitation (use at a concentration of 5 µg/µl), Chromatin Immunoprecipitation (use at a concentration of 2 - 3 µg/µl), Immunocytochemistry/Immunofluorescence (0.5 µg/ml), Immunohistochemistry (1 µg/ml, perform heat-mediated antigen retrieval befor IHC staining)
• Anti-H2A.Z (Abcam, ab4174) - species reactivity: human, mouse, rat, cow; validated applications: Western Blot (1:1000), Chromatin Immunoprecipitation (assay-dependent dilution), Immunocytochemistry/Immunofluorescence (dil. 1:1000)

Eukaryotic cell lines

Policy information about cell lines and Sex and Gender in Research

Cell line source(s)

• HeLa S3 (ATCC #CCL-2.2) cells were obtained from Cancer Research UK - Clare Hall Laboratories Cell Services Facility (South Mimms, UK).
• HeLa Kyoto BAC cell line expressing the C-terminally LAP (localisation and affinity purification)–tagged INO80 subunit ACTR5 was a gift from Matthias Mann (Reference: Hein, M. Y. et al., 2015, A Human Interactome in Three Quantitative Dimensions Organized by Stoichiometries and Abundances. Cell 163: 712–723 - PMID: 26496610; Cell line ID: MCP_ky_0007413). This cell line was originally generated in the lab of Anthony A. Hyman (Max Planck Institute for Molecular Cell Biology and Genetics, Dresden, Germany) by insertion of an engineered C-terminally LAP-tagged ACTR5 BAC transgene into the HeLa Kyoto cell line (RRID:CVCL_1922), an isolate of the original HeLa cell line (RRID:CVCL_0030; ATCC #CCL-2) derived by S. Narumiya (Kyoto University, Kyoto, Japan).
• MCF-7 cells (ATCC #HTB-22) were) were obtained from the Cell Services Facility of the IGBMC (Illkirch, France).
• IMR90 human fibroblast cells (ATCC #CCL-186) were purchased from ATCC.

Authentication

• The HeLa S3 cell line was authenticated by morphology by their ability to growth both in suspension culture and as round spherical cells in adhesion culture.
• The HeLa Kyoto BAC cell line expressing the C terminally LAP (localisation and affinity purification)–tagged INO80 subunit ACTR5 was validated by IP and western blot against the tagged ACTR5.
• The MCF-7 cell line was authenticated by morphology and by regularly testing the induction of estrogen-responsive genes by qPCR with gene-specific primers or global RNA-seq after 17beta-estradiol treatment.
• The INO80B-V5 knock-in MCF-7 cell line was authenticated by western blot against the V5 knock-in tag and by V5-IP followed by western blot against INO80B and Mass Spectrometry.
• IMR90 cells were purchased directly from ATCC and only maintained for a limited number of passages.

Mycoplasma contamination

All cell lines were tested and mycoplasma-free.

Commonly misidentified lines
(See ICLAC register)

No commonly misidentified cell lines were used in this study.
