# Supplementary material for: Decoding chromatin states by proteomic profiling of nucleosome readers
Source: Nature. 2024 Mar 6;627(8004):671–9. doi: 10.1038/s41586-024-07141-5 (PMC10954555; doi:10.1038/s41586-024-07141-5)
Supplement: Supplementary file 3 — Gel raw data and graph source data. [file 41586_2024_7141_MOESM3_ESM.pdf]

Fig. 5e

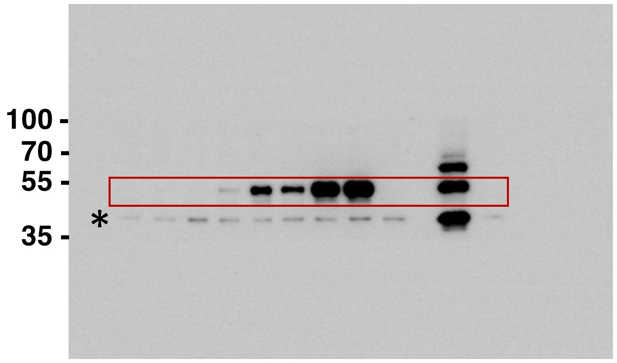

anti-INO80B

* Non specific signal

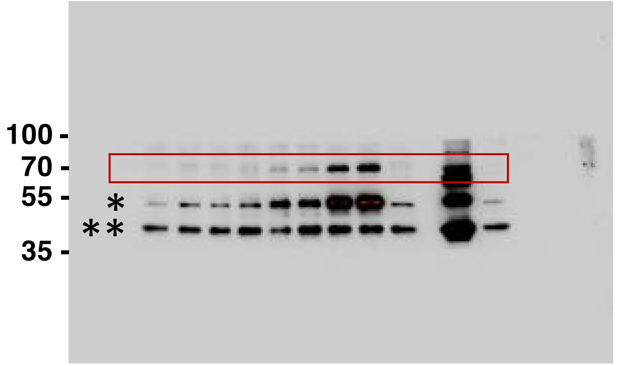

anti-ACTR5

* anti-INO80B; ** Non specific signal

Note: INO80B and ACTR5 WBs were performed sequentially using the same membrane

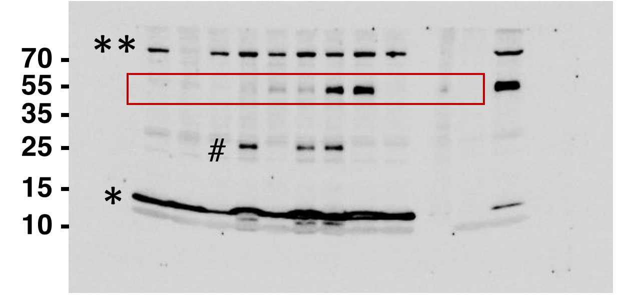

anti-TBRG1

* anti-H4

\#, ** Non specific signal

Note: TBRG1 and H4 were probed on a separate membrane.

Extended Data Fig. 2b

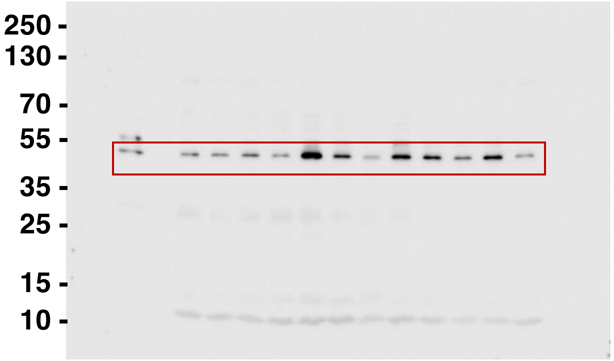

anti-CBX8

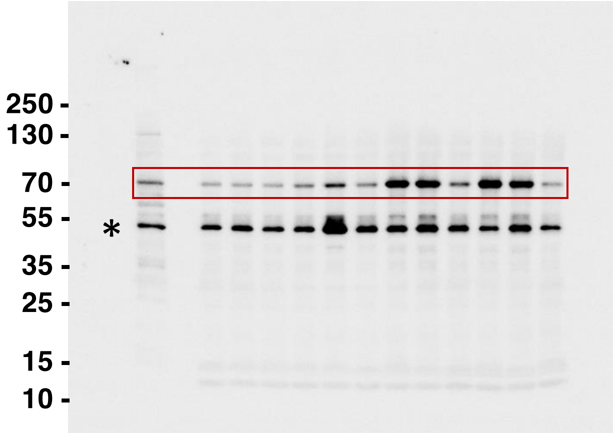

anti-CBX4

* anti-CBX8

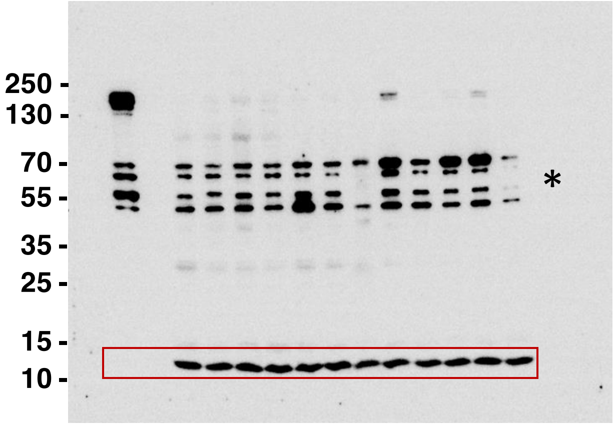

anti-H4

* anti-CBX4 & CBX8

Note: CBX4, CBX8 and H4 were probed sequentially using the same membrane.

Extended Data Fig. 5h

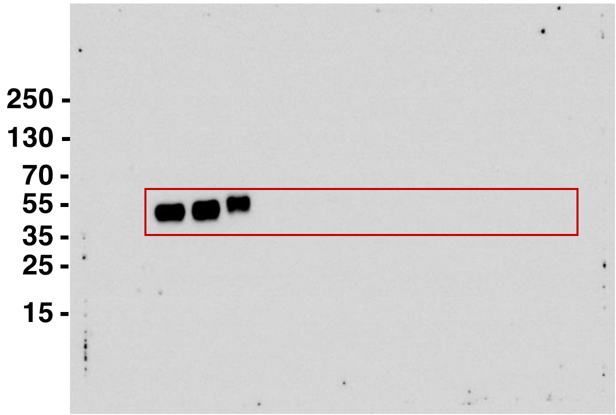

anti-V5 (INO80B)

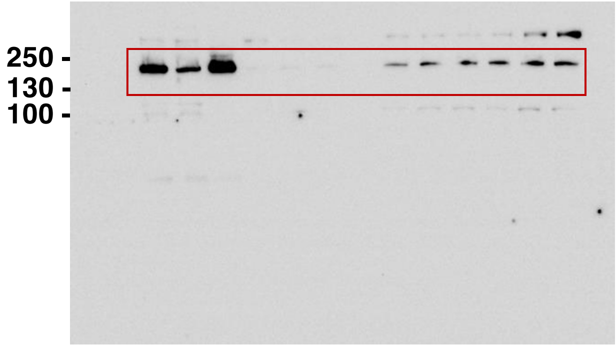

anti-INO80

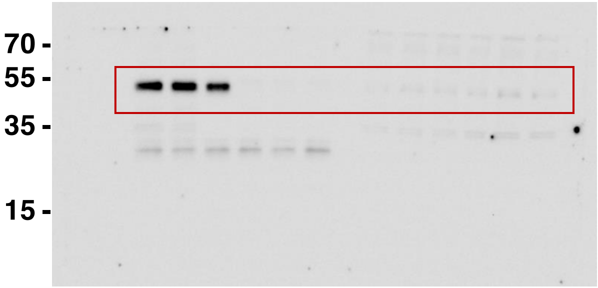

anti-TBRG1

Extended Data Fig. 5j

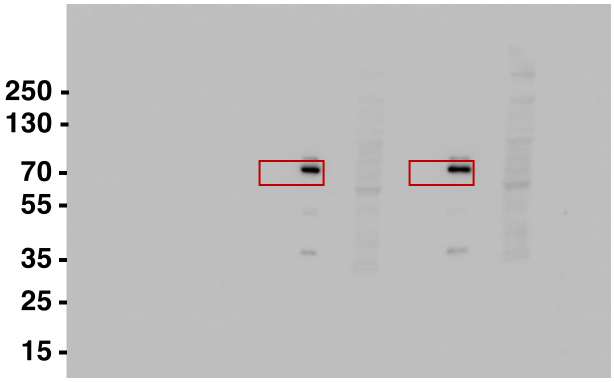

anti-ACTR5

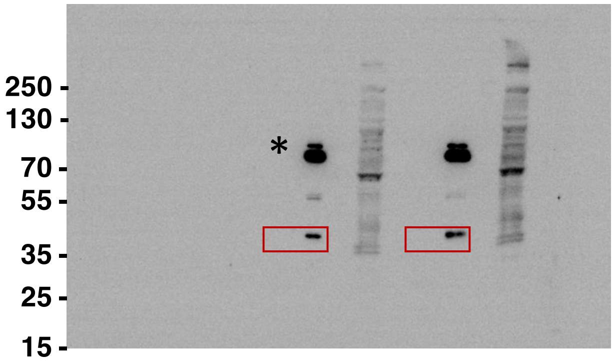

anti-INO80B * anti-ACTR5

Note: INO80B and ACTR5 were probed simultaneously using the same membrane

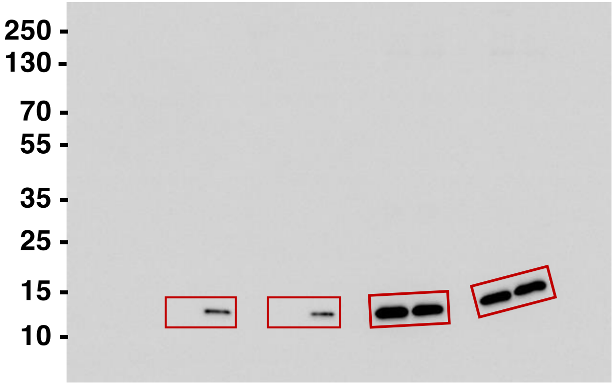

anti-H2B

Note: H2B was probed on a different membrane

Extended Data Fig. 5g

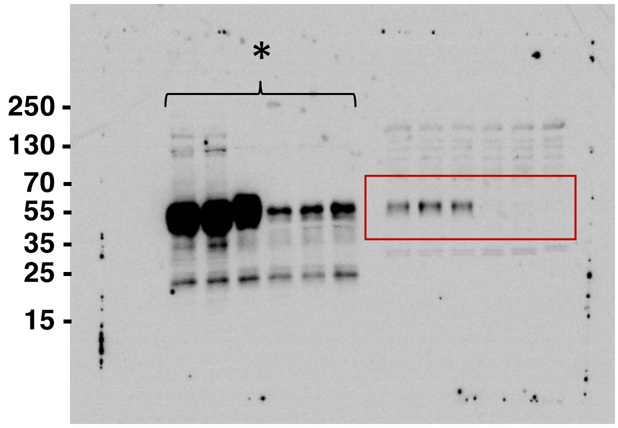

anti-V5 (INO80B)

Note: WB obtained by a longer exposure of the same membrane as shown in Extended Data Fig. 5h

* The first six lanes are IP samples corresponding to the experiment shown in Extended Data Fig. 5h

Sample size values for Fig. 3d

	Complex																										
**predictor**	**PBAF**	**BAF**	**EBAFA/B**	**GBAF**	**ATAC (exclusive subunits)**	**MLL3/4 (exclusive subunits)**	**ATAC**	**MLL3/4**	**NuRF**	**NuRD (exclusive subunits)**	**SAGA**	**NuRD**	**PCAF**	**TFTC**	**NuA4**	**NuA4 (exclusive subunits)**	**TFIID**	**SRCAP**	**MOZ/MORF**	**PRC1 (exclusive subunits)**	**PRC1**	**Integrator**	**Integrator (exclusive subunits)**	**INO80**	**Mediator**	**INO80 (exclusive subunits)**	**CHRAC**
**H3ac**	13	10	9	12	6	6	11	10	4	9	20	14	9	20	20	6	14	9	5	5	7	14	13	15	15	11	4
**H4ac**	16	13	12	12	6	6	11	10	4	9	20	14	9	20	20	6	13	9	5	5	7	13	12	15	15	11	4

Note: all other relevant information can be found in Supplementary Table 8.
